# High-Throughput Toxicity and Phenotypic Screening of 3D Human Neural Progenitor Cell Cultures on a Microarray Chip Platform

**DOI:** 10.1016/j.stemcr.2016.10.001

**Published:** 2016-10-27

**Authors:** Gregory J. Nierode, Brian C. Perea, Sean K. McFarland, Jorge F. Pascoal, Douglas S. Clark, David V. Schaffer, Jonathan S. Dordick

**Affiliations:** 1 Chemical and Biological Engineering and Center for Biotechnology & Interdisciplinary Studies, Rensselaer Polytechnic Institute, Troy, NY 12180, USA; 2 Chemical and Biomolecular Engineering, University of California, Berkeley, CA 94720, USA; 3 Bioengineering, University of California, Berkeley, CA 94720, USA; 4 Department of Bioengineering and Institute for Bioengineering and Biosciences, Instituto Superior Técnico, Universidade de Lisboa, Lisbon 1049-001, Portugal

**Keywords:** high-content screening, human neural stem cells, high-throughput microarray, screening, neurotoxicity, three-dimensional cell culture

## Abstract

A 3D cell culture chip was used for high-throughput screening of a human neural progenitor cell line. The differential toxicity of 24 compounds was determined on undifferentiated and differentiating NPCs. Five compounds led to significant differences in IC_50_ values between undifferentiated and differentiating cultures. This platform has potential use in phenotypic screening to elucidate molecular toxicology on human stem cells.

## Highlights

• Demonstrated chip platform for HTS of protein expression and toxicity of 3D cultures
• Dose-response viability and proliferation of a 24-compound library on human NPC lines
• Assessed differential toxicity between progenitors and differentiating progeny
• Identified five compounds more toxic to undifferentiated progenitors

## Graphical Abstract

**Figure:**
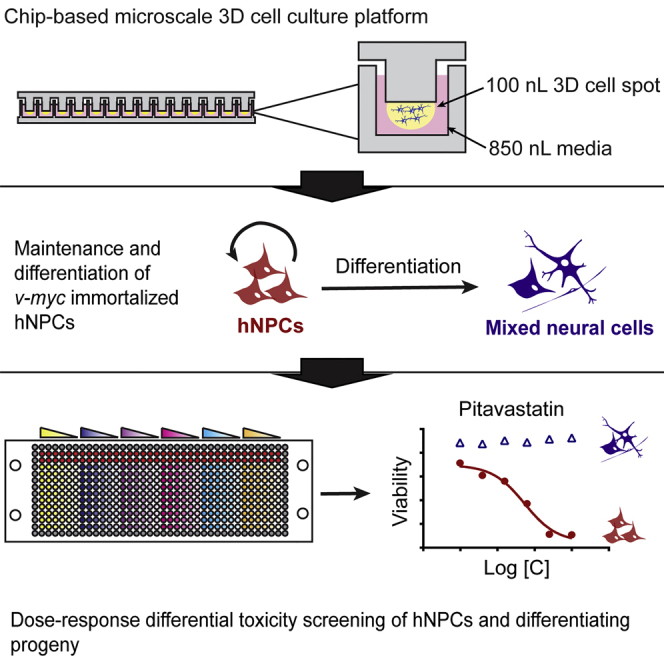

## Teaser

Dordick and colleagues demonstrate a 3D cell culture chip for high-throughput screening of immortalized human neural progenitor cells. The dose-response toxicity of 24 compounds was determined on undifferentiated and differentiating hNPCs. Five compounds led to significant differences in IC_50_ values between undifferentiated and differentiating cultures. This platform has potential use in phenotypic screening to elucidate molecular toxicology on human stem cells.

## Introduction

Human embryonic, induced pluripotent, and adult stem cells are invaluable tools for drug discovery, human toxicology, and studies on human development. Through controlled stem cell differentiation, large quantities of cell types from various tissues have been generated, including lung, liver, intestine, and neural to name a few (Lancaster et al., 2013, Spence et al., 2011, Takebe et al., 2013, Wetsel et al., 2011). In all cases, expansion in a stem or progenitor cell state is required to achieve large cell numbers, followed by differentiation into terminally differentiated fates. Such expansion and differentiation processes depend on myriad soluble factors, and cellular interactions with other cells and the extracellular matrix (ECM). This complexity has motivated the development of high-throughput screening tools to explore the large combinatorial signaling “space” associated with stem cell differentiation. The development of high-throughput screening platforms that emulate the complexity of natural stem cell microenvironments has provided basic insights into stem cell regulation, as well as enabling numerous applications (Lutolf et al., 2009, Soen et al., 2006). For example, toxicity screening systems that use human stem/progenitor cells and their terminally differentiated derivatives may help improve preclinical characterization of drug candidates and thereby reduce the extremely high attrition rates that plague drug development, often due to unforeseen toxicity (Hay et al., 2014, Ledford, 2011).

Cell-based microarrays have been used to screen for the effects of arrayed ECM proteins on neural stem cell proliferation and differentiation (Soen et al., 2006). The experimental platforms for these cell-based studies, however, have been almost uniformly focused on two-dimensional (2D) environments, despite the fact that three-dimensional (3D) approaches have gained increasing interest (Ranga et al., 2014). In particular, 3D culture models may better mimic the in vivo cellular microenvironment, which can be critical in phenotypic screens (Barcellos-Hoff et al., 1989, Yarmush and King, 2009). However, challenges remain in developing and implementing microarray-based 3D systems for screening purposes, including developing stable natural or synthetic matrices that enable rapid diffusion of soluble factors and reagents for 3D-based studies (Li et al., 2014, Ranga et al., 2014, Yarmush and King, 2009). Furthermore, samples arrayed on a surface often share the same culture medium, whereas it would be desirable to screen many liquid media compositions using a 3D culture platform. To address these limitations, we developed a microfabricated plastic “chip” system capable of 3D cell culture at the nanoliter scale. This platform, which has been described previously for rapid toxicity screening of compounds with human hepatocellular carcinoma cells, consists of two complementary chips that “stamp” together to generate up to 532 independent microscale cultures per chip (Kwon et al., 2014).

In the current work, we use this chip-based microarray platform to culture and perform high-content screening of a human neural progenitor cell (NPC) line, ReNcell VM, in 3D microscale cultures encapsulated within Matrigel. The NPCs were maintained in their multipotent state or induced to differentiate, and the resultant cultures were used to quantify expression of key cellular proteins, screen for acute toxicity and anti-proliferative effects of a diverse range of biologically active compounds, and investigate whether variable toxic sensitivities exist between undifferentiated and differentiating cells. Such information provides new insight into how miniaturization of 3D cell culture impacts NPC proliferation and differentiation for use in high-content, high-throughput screening of toxicity and cell phenotype.

## Results

### Characterization of 3D Microarray NPC Cultures

**Figure 1:**
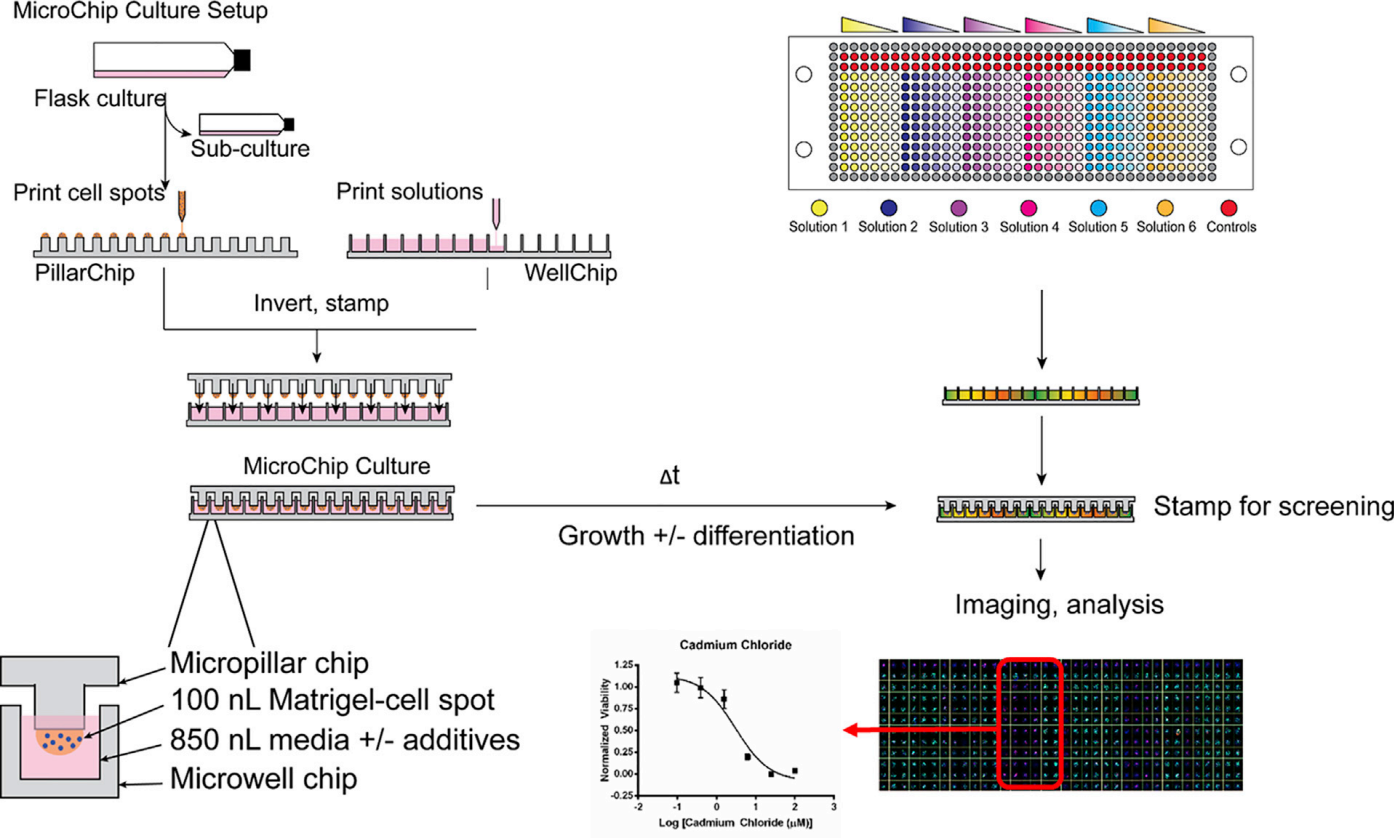
Microarray Chip Platform The platform consists of two complementary polystyrene microchips measuring 25 × 75 mm, each containing 532 micropillars (PillarChip) or microwells (WellChip) arranged in a 14 × 38 array. The feature-to-feature distance of pillars and wells is 1.5 mm with diameters of 750 μm and 1.2 mm, respectively. Cell spots consist of Matrigel-encapsulated cells spotted as 100 nL cultures atop micropillars and the microwells contain 850 nL of medium. Medium change is straightforward and consists of lifting a PillarChip from one WellChip and stamping into another containing fresh medium. Fluorescence-based endpoint assays are used to measure viability, proliferation, and protein expression of on-chip cultures.

The microarray chip platform consists of two complementary polystyrene microchips with 532 micropillars (PillarChip) or microwells (WellChip). Cells are encapsulated in a 3D matrix atop a PillarChip, which is submerged into a WellChip for on-chip culture (Figure 1). In general, 3D cultures require cumbersome imaging and analytical techniques (e.g., confocal imaging and stack reconstruction) (Li et al., 2015). To simplify this process, on-chip 3D cultures were rapidly dried for image processing and analysis; however, for samples labeled with fluorescent live-cell stains, this resulted in cell lysis. It was reasoned this cell lysis could be prevented with a lyoprotectant, and addition of 50 mM trehalose to the Dulbecco's phosphate-buffered saline (DPBS) wash solution prior to drying indeed preserved cytoplasmic staining of calcein, enabling the simplified imaging approach (Figure S1A).

**Figure 2:**
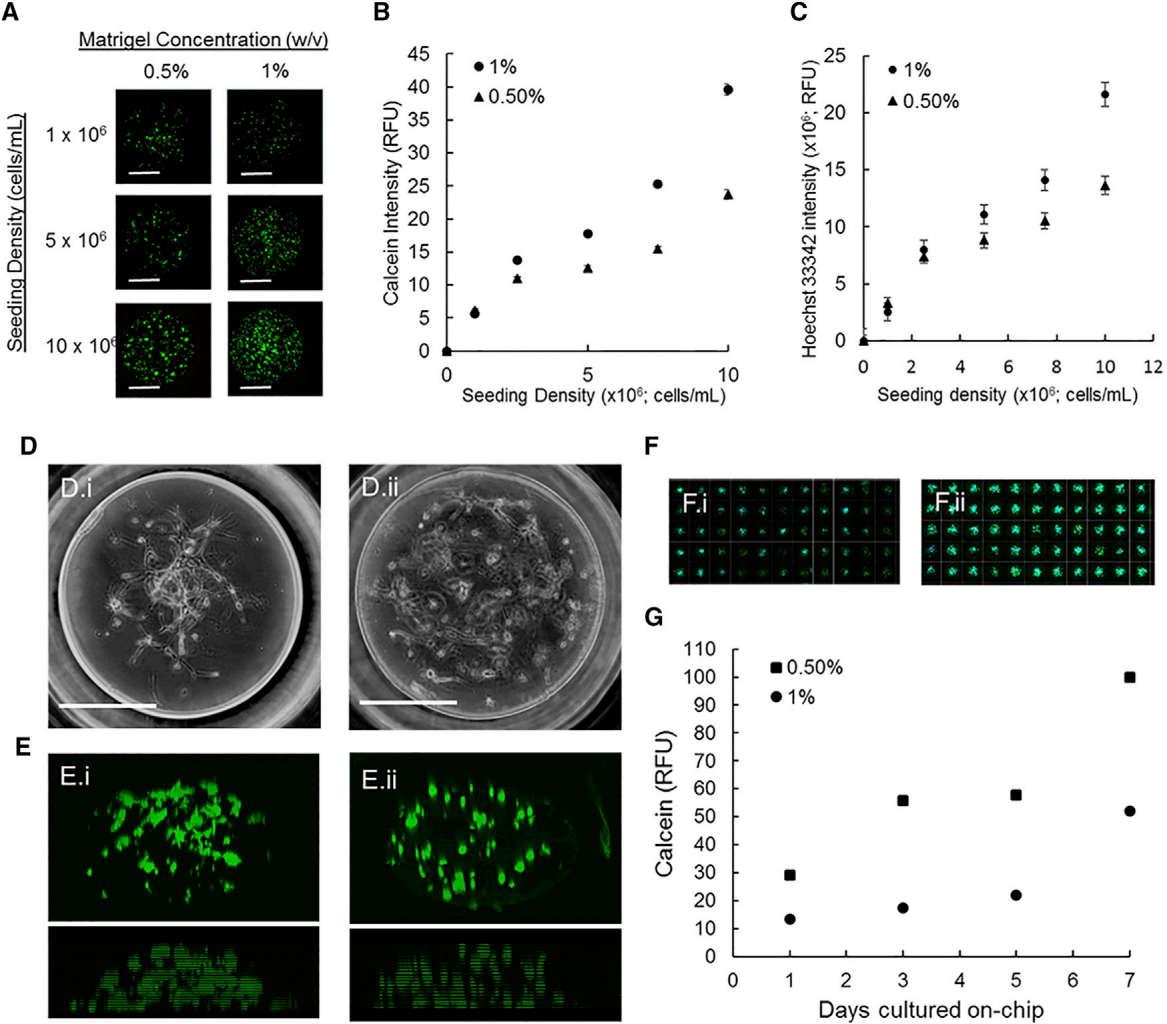
On-Chip Viability Assay Sensitivity and On-Chip NPC Culture Characterization (A–C) Representative fluorescence images of assayed cell spots, seeded with up to 1 × 10^7^ cells/spot in 0.5% or 1% Matrigel (A). The background adjusted mean fluorescence intensity ± SEM (n = 72 biological replicates) is plotted against seeding density in both (●) 0.5% and (▴) 1% (w/v) Matrigel for calcein (B) and Hoechst 33342 (C). (D and E) Phase contrast (D) and z stack (E) reconstructed confocal images of NPCs cultured on-chip in 0.5% (i) and 1% (ii) Matrigel for 3 days. (F) Growth assessed by calcein staining intensity is qualitatively apparent in fluorescent image montages (compiled with Cellomics software) when comparing staining between on-chip cultures after 1 (i) and 7 (ii) days of culture. (G) Quantified calcein intensity of ReNcell VM NPCs cultured on-chip in (■) 0.5% and (●) 1% Matrigel over time (one time-lapse experiment, where each point represents the mean ± SEM of 396 biological replicates). Scale bars, 300 μm.

The sensitivity of fluorescent live-cell stains to assess quantitatively cell spots was determined using on-chip cultures prepared with a range of cell densities (up to 1 × 10^7^ cells/mL). As anticipated, we observed a near-linear relationship between fluorescence intensity and seeding density for calcein and Hoechst 33342, which demonstrated that the fluorescence intensities are representative of living and total cells, respectively, within the 3D on-chip cultures (Figures 2A–2C). Single-cell analysis revealed on-chip post-printing viability of NPC cultures to be 84% ± 5% and 80% ± 6% (mean ± SD, n = 396 biological replicates) in 0.5% and 1% Matrigel, respectively, at 300 cells per spot (Figure S1B). ReNcell VM, the human NPC line used in this study, is a commercially available, *v-myc* immortalized region-specific human neural progenitor cell line derived from ventral mesencephalon bulk tissue dissections of a 10-week fetus (Donato et al., 2007). Arrayed, undifferentiated NPCs encapsulated in Matrigel (0.5% and 1% [w/v]) had a compact cylindrical/spherical shape (Figure 2D), notably different from their spread and flattened appearance in 2D monolayer cultures (Figure S1C). Calcein staining indicated that the cells were uniformly distributed throughout the cell spots, and the estimated average spot height (n = 3 biological replicates) was 250 ± 17 μm and 204 ± 5 μm for 0.5% and 1% Matrigel, respectively (Figure 2E).

The effects of encapsulating and soluble Matrigel concentration, fibroblast growth factor 2 (FGF2) and epidermal growth factor (EGF) concentrations, seeding density, and frequency of medium change were screened in a 2^5^ factorial design experiment, which revealed daily medium change had a significant impact on growth and viability on-chip, and was thus employed in subsequent experiments (Figure S2). The concentration of EGF and FGF2, and soluble or encapsulating Matrigel, had statistically insignificant effects on cell viability and growth. In addition, cultures seeded at 500 cells/spot had a significantly higher calcein fluorescence than those seeded at 300 cells/spot, which demonstrated that the cultures remained viable at higher cell densities.

The effect of culture time on NPC proliferation when cultured within Matrigel on-chip was measured in a time-lapse experiment. Four on-chip cultures were prepared with either 0.5% or 1% Matrigel, and viability across an entire chip was measured after 1, 3, 5, and 7 days of culture. As anticipated, calcein fluorescence intensity per spot increased over time (Figures 2F and 2G). NPCs cultured on-chip experienced a lag phase (∼1–2 days) followed by growth with calculated cell doubling times of ∼67 and ∼70 hr for 0.5% and 1% Matrigel, respectively. Ultimately, 1% Matrigel encapsulation resulted in increased physical stability of cell spots and was used for subsequent screening.

### Protein Expression of NPCs in 3D Microscale Cultures On-Chip

Several proteins associated with the maintenance and/or function of various cell states were used as markers to characterize undifferentiated and differentiated NPC phenotypes. Undifferentiated NPCs express the intermediate filament Nestin (NES) and transcription factor SOX2 (Komitova and Eriksson, 2004, Park et al., 2010), and can express additional markers such as glial fibrillary acidic protein (GFAP), an intermediate filament also expressed in terminally differentiated astrocytes (Goldman, 2003). Differentiating NPCs begin to express proteins associated with specific terminal lineages, e.g., astrocyte differentiation can be characterized by increased expression of GFAP and S100β, a regulatory calcium-binding protein (Bignami et al., 1972, Markiewicz and Lukomska, 2006, Raponi et al., 2007). Analogously, progenitor cells differentiating into neurons transiently express doublecortin (DCX), a microtubule-associated protein, before terminal differentiation and expression of βIII tubulin (TUBB3), a microtubule protein (Couillard-Despres et al., 2006, Roskams et al., 1998). Cells differentiating into oligodendrocytes express CNPase (CNP), an enzyme involved in myelination (Sprinkle, 1989).

Withdrawal of EGF and FGF2 from culture medium is expected to induce differentiation of ReNcell VM, during which time the stem/progenitor cells experience significant changes in morphology, protein expression, and function to develop into terminally differentiated phenotypes (Donato et al., 2007, Sun et al., 2008). Immunofluorescence characterization of protein markers associated with undifferentiated and differentiated cell states before and after induction of differentiation has, to our knowledge, not been done with this cell line. Thus, we proceeded to assess differentiation induced by EGF and FGF2 withdrawal using both immunofluorescence and western blot analysis. To address antibody quality, primary antibodies were validated using human cell lines to verify specificity for immunofluorescence (Figures S3A–S3D).

**Figure 3:**
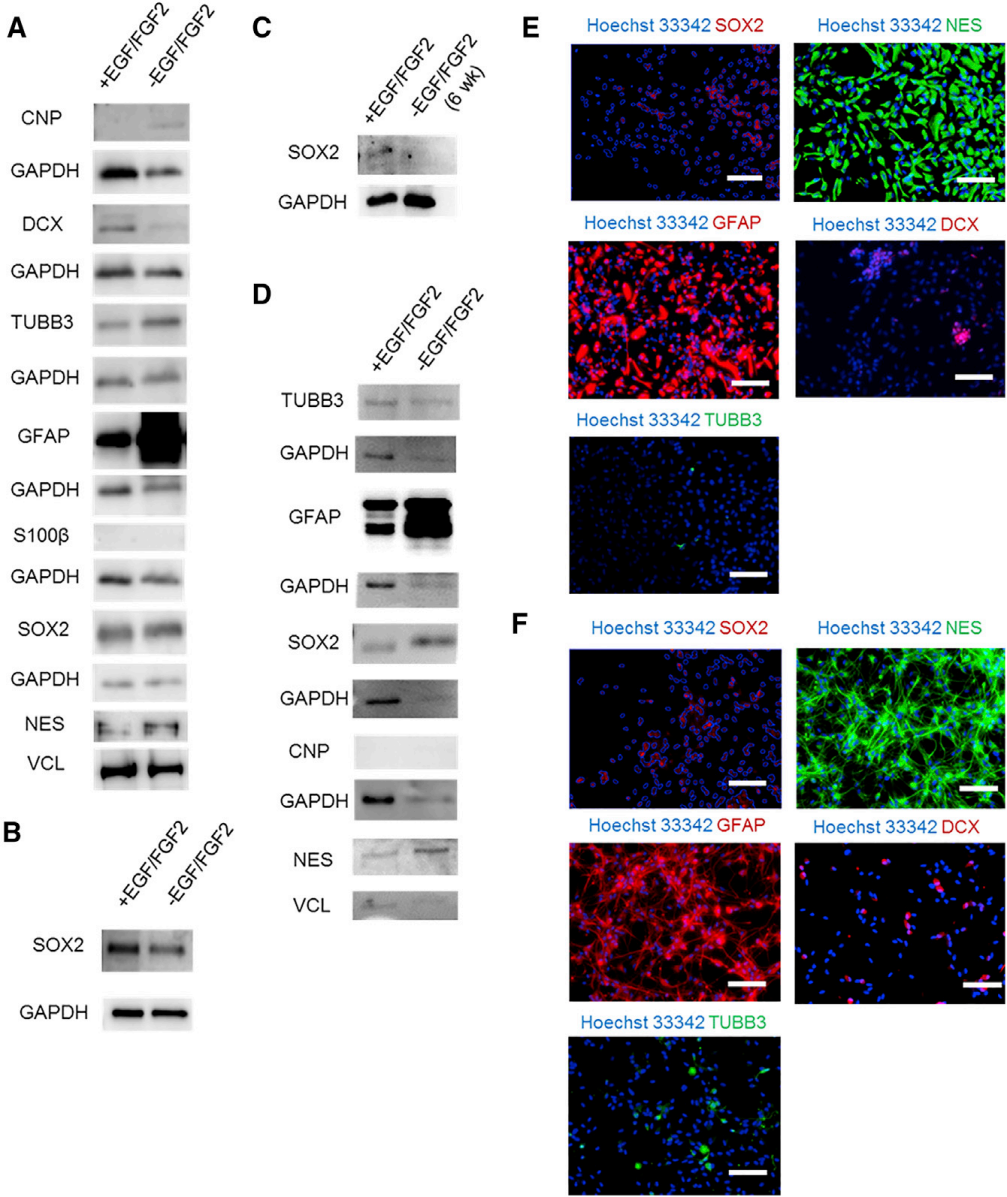
Off-Chip Analysis of ReNcell VM NPC Protein Expression in 2D Monolayer and 3D Matrigel Cultures (A) Western blot analysis of fate-specific protein markers in ReNcell VM 2D monolayer cultures before and after a 10-day culture without EGF and FGF2 to induce differentiation. (B) SOX2 western blot analysis in a 2D monolayer culture with an additional antibody specific for SOX2 (Cell Signaling Technologies). (C) SOX2 western blot analysis in 2D monolayer culture following a 6-week differentiation. (D–F) Western blot analysis of ReNcell VM cultured off-chip in 3D 1% Matrigel before and after growth factor withdrawal (D). Immunofluorescence analysis of undifferentiated (+EGF/FGF2) (E) and differentiating (−EGF/FGF2). (F) ReNcell VM cultured in 2D monolayer. Merged fluorescence images of the indicated protein and Hoechst 33342 (used as a counterstain) are depicted with the exception of SOX2 (nuclear staining), where the Hoechst 33342 nuclei were used to create an overlay mask. Brightness and contrast of single-channel images were adjusted with ImageJ to eliminate background using samples stained with fluorescent secondary antibody only prior to merging. GAPDH and vinculin were used as loading controls. Scale bars, 100 μm.

ReNcell VM cultured as monolayers (2D) or embedded within 1% Matrigel (3D) were cultured with and without EGF and FGF2 to assess protein expression. For undifferentiated 2D cultures (+EGF/FGF2), expression of DCX, TUBB3, GFAP, SOX2, and NES was detected by both western analysis (Figure 3A) and immunofluorescence (Figure 3E). Differentiation induced through removal of EGF and FGF2 for 10 days resulted in drastic morphological changes (Figure S1C). Western analysis revealed that the loading control-relative expression of TUBB3, GFAP, and CNP increased while, unexpectedly, the expression of NES and SOX2 persisted (Figure 3A). A second SOX2 antibody was used in a repeat experiment to account for the possibility of a non-specific signal, yielding a similar result (Figure 3B). Notably, 2D differentiation for 6 weeks resulted in substantial SOX2 downregulation (Figure 3C). Immunofluorescence analysis revealed that differentiating ReNcell VM continued to express SOX2, NES, DCX, and GFAP while the number of cells expressing TUBB3 increased (Figure 3F) after 10 days. S100β and CNP expression was undetected in 2D cultures by immunofluorescence (data not shown). Western analysis of 3D slab cultures revealed an increase in relative expression of TUBB3 and GFAP after culture without EGF and FGF2 (Figure 3D), similar to 2D. However, there were also notable differences between monolayer and 3D slab differentiation. Specifically, in the 3D slab cultures CNP was undetected, and there was an increase in relative expression of SOX2 and NES after 10 days without EGF and FGF2.

**Figure 4:**
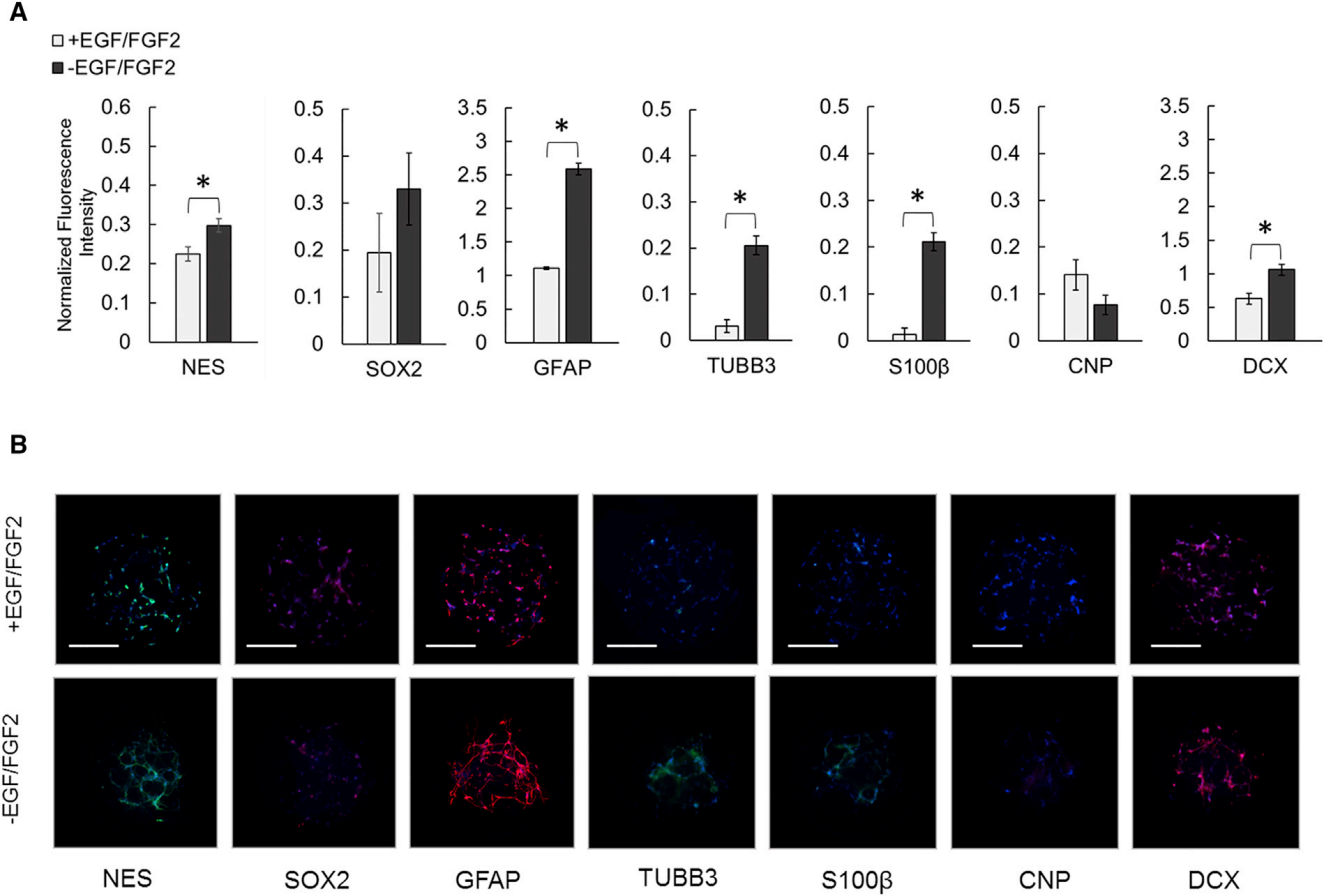
On-Chip Analysis of ReNcell VM NPC Protein Expression in Microscale 3D Matrigel Cultures (A) Hoechst 33342-normalized immunofluorescence detection of cell fate-specific protein markers within on-chip NPC cultures before and after a 10-day culture without EGF and FGF2. Mean ± SEM plotted for 36 independent biological replicates per marker for each condition. ^∗^p < 0.05 (two-tailed Student's t test) indicates the difference between undifferentiated and differentiating cultures. (B) Representative images of immunofluorescence stained on-chip NPC cultures before and after a 10-day culture without EGF and FGF2 (green or red); Hoechst 33342-counterstained nuclei (blue). The Cellomics analysis suite was used for single-channel brightness and contrast adjustment to eliminate background signals as determined from samples stained only with fluorescently labeled secondary antibody. Scale bars, 300 μm.

Analysis of protein expression on-chip was performed using a modified in-cell, on-chip immunofluorescence assay. Sensitivity of the assay was first determined by spotting undifferentiated NPCs at varied cell densities (1, 2.5, and 5 × 10^6^ cells/mL) in 1% Matrigel followed by immunofluorescence-based detection of NES and GFAP. As anticipated, the total fluorescence intensity of each marker increased with spotting cell density (Figure S3E). Dividing immunofluorescence intensity by Hoechst 33342 intensity within each spot resulted in similar normalized values for all seeding densities (Figure S3F), suggesting that Hoechst 33342 intensity normalization accounts for differences in cell number when analyzing protein expression on-chip. Expression of protein markers indicative of cell fate was then quantified on-chip after a 5-day culture with EGF and FGF2 and again after 10 subsequent days without EGF and FGF2 to induce differentiation. Undifferentiated cultures (+EGF/FGF2) expressed SOX2, NES, GFAP, and DCX after culture on-chip (Figure 4A). Differentiation (−EGF/FGF2) resulted in growth of cell extensions, depicted in the immunofluorescence images of GFAP and NES (Figure 4B). Expression of SOX2 persisted while NES, TUBB3, GFAP, S100β, and DCX expression increased (p < 0.05, two-tailed t test; Figure 4A) in differentiating cultures.

Taken together, these results suggest that while EGF and FGF2 withdrawal is sufficient for inducing increased expression of terminal markers, the extent of differentiation is moderate after only 10 days. Nonetheless, the changes in protein expression support the conclusion the NPCs cultured without EGF and FGF2 are in the *process* of differentiating into primarily pan-astrocytic and neuronal lineages.

### Acute Toxicity Screening of NPCs in 3D Microarray Cultures

To demonstrate the high-throughput screening capability of the chip platform, we evaluated the dose-response effects on cell viability of a 24-compound library with diverse structural and biological properties, consisting of approved drugs, heavy metals, and pesticides, some of which are regarded as acutely neurotoxic (Table S2). Cell proliferation was then tested for a subset of this library. For both viability and proliferation, the effect of the compounds was tested on both undifferentiated and differentiating NPC cultures, which would uncover cell-state specific (self-renewal versus differentiation) differences in toxicity.

**Figure 5:**
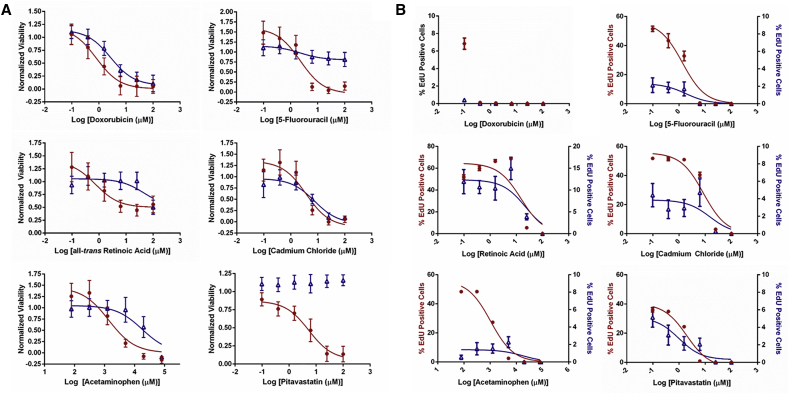
Dose-Response Viability and Proliferation Effects of Select Compounds on Undifferentiated and Differentiating ReNcell VM NPCs (A) Vehicle control-normalized dose-response viability of undifferentiated (●) and differentiating (**Δ**) NPCs plotted against log(concentration [μM]). Mean viability ± SEM is plotted for each concentration as determined from 30 biological replicates per dose. Sigmoidal fits to the data are plotted as solid lines. (B) Dose-response EdU incorporation of undifferentiated (●, left axis) and differentiating (**Δ**, right axis except DOX) NPCs plotted against log(concentration [μM]). Mean % EdU positive nuclei ± SEM is plotted for each concentration as determined from ten biological replicates. Dose-response viability and proliferation results for compounds not presented herein are available in Figures S4 and S5.

On-chip cultures of undifferentiated and 10-day differentiated NPCs were stamped with WellChips filled with compound-containing solutions and incubated for 72 hr prior to viability assessment. Vehicle-control normalized viabilities were plotted against compound concentration to generate dose-response plots (Figures 5A and S4). The acute viability screen identified 16 compounds that substantially decreased viability (≥50% killing) relative to controls (Table S3). The dose-response data for nine of these compounds were suitable for sigmoidal curve fitting and calculation of a median inhibitory concentration (IC_50_) value (Table 1). The sigmoidal fits for undifferentiated and differentiating NPCs were compared to assess whether differential sensitivities (i.e., different IC_50_ values) existed for the screened compounds. Statistically significant (p < 0.05, two-tailed t test) IC_50_ values were observed between undifferentiated and differentiating cultures for retinoic acid, doxorubicin (DOX), 5-fluorouracil (5-FU), pitavastatin, and acetaminophen. In addition, the IC_50_ values in response to cytosine arabinoside (CA) were notably different, although the statistical difference warranted further investigation (p < 0.10, two-tailed t test). The resulting differences in viability between undifferentiated and differentiating cultures in response to pitavastatin and 5-FU (Figures 5A and S4) was particularly interesting, with substantially greater killing of undifferentiated cultures for these compounds.

Differential toxic effects could be explained, in part, as a consequence of the different proliferative states between the undifferentiated and differentiating NPCs. To this end, the thymidine analog, 5-ethynyl-2′-deoxyuridine (EdU), was used to detect actively proliferating cells on-chip following exposure to compounds or vehicle control to determine dose-response effects on proliferation (Figures 5B and S5), and sigmoidal curves were fit, when appropriate, to obtain IC_50_ values (Table 1). We found ∼60% of undifferentiated NPCs incorporated EdU over a 24-hr period as opposed to ∼3% of differentiating cultures. Following exposure to 5-FU, a known anti-proliferative agent, we observed a sharp decrease to no EdU incorporation in both undifferentiated and differentiating NPC cultures. In consideration with the differential cytotoxicity induced by 5-FU, this result confirms the different proliferation rates of undifferentiated and differentiating culture contributes to their differential sensitivities.

## Discussion

There is considerable interest in developing high-throughput technologies for microscale analysis and screening of both undifferentiated and differentiated human stem/progenitor populations to aid drug screening and toxicity analysis (Bhatia and Ingber, 2014, El-Ali et al., 2006). Animal models are often used to assess toxicity, including neurotoxicity, but they are associated with high costs and results that often have poor translation to humans (DiMasi et al., 2003, Hay et al., 2014). Human cell-based microarray screening systems are attractive because of automated systems for handling liquids that can enable screening with minimal reagent consumption. This is of particular interest in elucidating the influence of ECM factors and/or signaling pathways on stem/progenitor function and differentiation and to investigate the large combinatorial space associated with their respective microenvironments.

In the present work, we have demonstrated a chip-based microarray platform that allows for simple medium exchange on microscale 3D cultures. Importantly, on-chip growth and differentiation of a human progenitor cell line was possible for more than 2 weeks. In addition, sensitive fluorescence-based assays were compatible to screen for protein expression, cytotoxicity, and proliferation. The system presented herein is advantageous for several reasons. Miniaturization of 3D cultures, which may better emulate in vivo conditions, permits investigation under conditions where diffusional limitations are negligible (Ranga et al., 2014, Meli et al., 2014). Moreover, the modularity of a system with two complementary chips (one for cells and one for medium components) that stamp together to generate the high-throughput cell culture is potentially suitable for 3D parallel screening. This is fundamentally distinct from other high-throughput 3D microscale culture platforms, e.g., in 1536-well plates, where medium exchange is cumbersome and specialized automated systems are often optimized for 2D cultures, which restricts the duration of culture and/or screening (Ranga et al., 2014).

The growth rate of ReNcell VM cultured on-chip in 3D Matrigel was roughly three times slower than that reported for 2D cultures (Donato et al., 2007, Meli et al., 2014). The on-chip 3D cultures are prepared at a medium-to-cell ratio that is about half that typically used with larger-scale (i.e., flask) 2D cultures. Thus, the relative quantity of available nutrients as a result of scale-down could contribute to delayed growth. In agreement with this, our factorial screen identified that more frequent medium change led to higher numbers of viable cells. We reasoned it was unlikely that diffusional limitations were contributing to slower cellular growth with our ∼200 μm cell spots because Ranga et al. (2014) demonstrated that soluble nutrients rapidly diffused throughout PEG-based hydrogels of ∼500 μm in thickness, while Meli et al. (2012) reported that oxygen diffusional limitation in 3D microscale cultures was negligible. Nonetheless, crosslinked Matrigel has been reported to have pore sizes <2 μm, which could result in physical constraints on cell growth in the encapsulated environment (Zaman et al., 2006). Thus, the slower growth observed on-chip could be a result of the need for NPCs to remodel and/or displace the encapsulating matrix to accommodate cell growth and cell-cell interactions. Regardless, conditions that mediate slow growth can be compatible with stem cell differentiation.

Our observation that the expression of multipotency markers (SOX2 and NES) persisted in 2D cultures, and even increased relative to undifferentiated cultures in 3D after 10 days of differentiation, was of note. Nevertheless, it has been demonstrated that NES expression decreases slowly; human midbrain neural progenitors have been shown to retain NES expression beyond 4 weeks of differentiation both in vitro and in vivo (Sun et al., 2008, Zhang et al., 2001). However, SOX2 expression is expected to decrease following differentiation into an oligopotent cell state (Bani-Yaghoub et al., 2006, Graham et al., 2003). ReNcell VM was immortalized by *v-myc* gene transduction of primary fetal neural progenitors. Recently Pino-Barrio et al. (2015) reported that inducing *v-myc* expression in NPCs increases SOX2 expression (Donato et al., 2007). Therefore, it is possible that overexpression of *v-myc* impacts the downregulation of SOX2 during differentiation of ReNcell VM, which could prolong or interfere with terminal differentiation. However, the downregulation of SOX2 after a 6-week differentiation suggests that *v-myc* immortalization does not inhibit the eventual downregulation of this multipotency marker.

Removal of growth factor-mediated self-renewal signaling in NPC cultures should result in increased expression of terminal differentiation markers (Graham et al., 2003, Sun et al., 2008). Indeed, in 2D monolayer cultures, ReNcell VM increased expression of proteins associated with terminal differentiation into astrocytes, neurons, and oligodendrocytes. Other studies have reported similar results with ReNcell VM after differentiation induced by growth factor withdrawal in monolayer culture (Donato et al., 2007, Hoffrogge et al., 2006, Lange et al., 2011), and terminal neuronal differentiation has been limited without employing methods aimed at directing neuronal differentiation. For example, Lange et al. (2011) reported that <3% of cells expressed TUBB3 after 7 days of differentiation induced by growth factor withdrawal. In 3D Matrigel, observed changes in protein marker expression upon differentiation were notably different compared with 2D cultures, which suggests that Matrigel impacts the spontaneous differentiation of ReNcell VM. Matrigel is a complex mixture of diverse ECM components, including proteoglycans, which may also impact differentiation. In addition, self-renewal growth factors (EGF and/or FGF2) may remain in the 3D matrix even after their removal from medium due to cytokine-matrix interactions, which could influence the progression of NPC differentiation. Indeed, Akashi et al. (2005) observed reduced diffusion of FGF2 through Matrigel matrices, suggesting that its elimination from the microenvironment could be delayed.

Moderate increased expression of terminal markers with retained expression of multipotency markers after 10 days suggests that spontaneous terminal ReNcell VM differentiation progresses slowly, and this may not be unique. Shi et al. (2012) reported that terminal differentiation of iPSC-derived forebrain neural progenitors entailed a 20-day culture. Furthermore, Choi et al. (2014) reported differentiating ReNcell VM for 6 weeks or longer to derive high proportions of terminally differentiated cells in 3D Matrigel. Thus, long-term differentiation may be required to obtain a high degree of terminally differentiated neural cells with ReNcell VM. Nonetheless, while not substantially terminally differentiated, ReNcell VM cultured without growth factors for 10 days were distinct from those grown in the presence of EGF and FGF2 and are likely a mixture of NPCs at various stages of differentiation.

Of the 16 compounds that substantially decreased viability, seven had IC_50_ concentrations <100 μM. This included acutely toxic compounds (e.g., paraquat, CdCl_2_) with IC_50_ values consistent with literature values (Breier et al., 2008). Compounds without potent neurotoxicity (e.g., acetaminophen, caffeine, diphenhydramine) were found to have no cytotoxicity at functionally relevant concentrations, and only at concentrations associated with systemic human toxicity did decreases in viability occur (Brent et al., 2011, Clemedson et al., 2007, Radovanovic et al., 2000). PbCl_2_ and MnCl_2_ did not reduce cell viability for either undifferentiated or differentiating cultures. Their neurotoxicity is associated with complex mechanisms that often do not manifest as acute cytotoxicity (Neal and Guilarte, 2013). Taken together, these results begin to define the utility of the immortalized ReNcell VM for assessing chemical neurotoxicity.

Five compounds elicited differential effects on undifferentiated and differentiating NPCs. All-*trans* retinoic acid is an important endogenous neurodevelopmental signaling small molecule that can also act as a developmental teratogen when dysregulated (Lammer et al., 1985, McCaffery et al., 2003), and undifferentiated NPCs exhibited a lower IC_50_ in response to retinoic acid versus differentiating NPCs. In addition, retinoic acid increased the average number of proliferating cells within differentiated cultures by greater than 3-fold at lower concentrations. Notably, the neurodevelopmental effects and sensitivity of rats to retinoic acid has previously been found to be dependent on the concentration and onset of exposure (McCaffery et al., 2003). As highlighted above, the acetaminophen concentrations needed to observe toxicity were above those that would be pharmacologically effective, although a large difference in sensitivity was observed between undifferentiated and differentiating cultures.

Pitavastatin, a 3-hydroxy-3-methyl-glutaryl-coenzyme A (HmG-CoA) reductase inhibitor, was cytotoxic only to undifferentiated NPCs, whereas another HmG-CoA reductase inhibitor, cilastatin, had no effect. Similarly, 5-FU was substantially more toxic to undifferentiated cultures and resulted in nearly complete killing while minimally impacting differentiating cultures. Both drugs were also found to inhibit the proliferation of NPC cultures, which suggests that the mechanism of toxicity was predominantly anti-proliferative (in agreement with their known mechanisms of toxicity) because they did not decrease the viability of slowly proliferating differentiating NPCs (Chan et al., 2003, Diasio and Harris, 1989). The observation that pitavastatin inhibited proliferation and that cilastatin did not is consistent with the finding of Chan et al. (2003) that statins have highly variable anti-proliferative effects on various cell lines and cancers.

DOX and CA are well-known chemotherapeutics with anti-proliferative and cytotoxic activity (Gewirtz, 1999, Momparler, 1982). For undifferentiated NPCs, DOX gave lower NPC viability (lower IC_50_), and to a lesser extent so did CA (p < 0.10), in comparison with differentiating NPCs. Because ∼3% of differentiating NPCs actively proliferated, the effects of DOX and CA suggest that cytotoxicity was the predominant mechanism of killing differentiating NPCs. This result also suggests that undifferentiated NPCs may be more sensitive to these compounds as a result of both anti-proliferative and cytotoxic mechanisms being effective against the undifferentiated, highly proliferative cell state.

The results presented herein demonstrate the application of a microarray chip platform as a sensitive screening tool capable of detecting differential toxicities of biologically active compounds in human NPCs of varied levels of differentiation. Given the need to develop in vitro screening platforms that can assess the activity and toxicity of chemicals on human cells, the chip platform may serve as a 3D phenotypic screening tool to better predict toxicity of, or identify, bioactive compounds in human stem/progenitor cells. Further research on non-immortalized NPCs may provide useful insights into the influence of specific compounds on stem cells and their progeny of various fates.

## Experimental Procedures

### Human Neural Progenitor Cell Culture

ReNcell VM (Millipore) was used in this study at passage number 16 or lower. Cells were grown on laminin (Sigma)-coated T25 or T75 tissue-culture-treated flasks in complete growth medium composed of ReNcell Maintenance Medium (Millipore) containing 20 ng/mL FGF2 (Millipore), 20 ng/mL EGF (Millipore), and 1% (v/v) penicillin-streptomycin (Gibco). Cells were passaged using Accutase and re-plated at 10,000 cells/cm^2^ when 90% confluent. Medium was changed the day after passaging and every second day after that. For off-chip 3D culture, ReNcell VM were seeded at 890,000 cells/mL in 100 μL of 1% Matrigel in 8-well chamber microscope slides (Nunc) with daily medium change. All cultures were maintained at 37°C and 5% CO_2_.

### 3D Microarray Culture Preparation

Polystyrene micropillar and microwell chips (SEMCO) were exposed to UV (302 nm) light (9 cm from source) for 4 hr using a 96-W transilluminator (Syngene GVM-30), following a similar procedure to that of Kohen et al. (2009), which helped maintain the cell spots atop the micropillars without covalent attachment. An enclosed MicroSys 5100-4SQ (DigiLab) non-contact robotic microarray spotting system was used for printing. During operation, the relative humidity was maintained above 95% to reduce evaporation. The MicroSys 5100-4SQ spotting head was modified with two water-block assemblies for circulation of chilled ethylene glycol-water to maintain the printer head between 2 and 8°C during Matrigel spotting.

To prepare microarray cultures, 850 nL of complete growth medium was printed into WellChips and covered with gas-permeable sealing membranes (Diversified Biotech) and stored in an incubator (37°C and 5% CO_2_). High-concentration growth factor-reduced Matrigel (Corning Life Sciences) was used for 1% (w/v) encapsulation and growth factor-reduced phenol red-free Matrigel (Corning Life Sciences) was used for 0.5% (w/v) encapsulation. Cooled suspensions of newly passaged ReNcell VM were mixed with Matrigel in a ratio such that the final cell concentration was 3 × 10^6^ cells/mL, unless otherwise noted, with final Matrigel concentrations of either 0.5% or 1% (w/v) and stored on ice until printing. The cooled microarray system was used to print the Matrigel-cell mixtures onto PillarChips (∼300 cells within each spot). Following printing, the PillarChips were incubated 4 min at 25°C followed by 10 min at 37°C in a humid chamber to polymerize the Matrigel. After gelation, a PillarChip was stamped into a WellChip containing warmed complete growth medium and stored in a humidified chamber for culture. The medium was changed daily unless otherwise noted. Periphery wells were not analyzed to prevent potential evaporation from affecting the results.

### Live/Dead Viability Assay

Cell viability was determined in 3D on-chip cultures using a mammalian cell viability assay (LIVE/DEAD, Invitrogen). Prior to the assay, dead cell controls were prepared with 30 min incubation in a WellChip containing either complete medium or 0.5% (w/v) saponin-DMEM/F12 medium in its microwells. PillarChips were rinsed twice with warm DPBS and incubated for 25 min in DPBS with 1 g/L glucose, 4 μM calcein-AM, 2 μM ethidium homodimer-I, and 5 mg/mL Hoechst 33342 at 5% CO_2_ and 37°C. After staining, PillarChips were rinsed twice with warm DPBS and incubated in DPBS containing 50 mM trehalose (Sigma) for 10 min before rapid drying with nitrogen and storage in the dark. Dried PillarChips were imaged with a Thermo Scientific Cellomics ArrayScan VTI high-content system.

### In-Cell Immunofluorescence Assays

Undifferentiated NPCs were plated on laminin-coated 96-well plates and cultured for 3 days in complete growth medium prior to fixation. Differentiated NPCs were prepared by expanding undifferentiated NPCs for 3 days, followed by differentiation in medium without EGF and FGF2 for 10 days with daily medium change to emulate on-chip conditions. Cells were rinsed with warm DPBS and fixed with 4% (w/v) formaldehyde and 0.25% (w/v) glutaraldehyde in DPBS for 20 min at room temperature. Fixed cells were permeated with 0.25% (v/v) Triton X-100 in DPBS for 10 min, rinsed with DPBS, and quenched for 30 min in DPBS with 2 mg/mL sodium borohydride (Sigma). Cells were blocked overnight at 4°C in DPBS with 5% (w/v) BSA and 1% (v/v) goat serum and incubated in primary antibody solutions diluted into DPBS containing 1% (w/v) BSA and 10 ppm anti-foam C (Sigma) overnight at 4°C. Samples were washed three times with DPBS for 2 hr before repeating the antibody incubation step for secondary antibodies. A complete list of primary and secondary antibodies, vendors, and dilutions used is presented in Table S1. After incubation with secondary antibodies, samples were washed three times with DPBS for 2 hr before 10 min incubation in DPBS with 5 μg/mL Hoechst 33342 and a final rinse with DPBS. An inverted fluorescent microscope was used for imaging and ImageJ was used to process images.

On-chip undifferentiated NPCs were analyzed after 5 days and differentiated cells after a 10-day differentiation following a 5-day expansion. Antibody incubations were performed in 750 nL of antibody containing solutions on-chip. After the final washes, PillarChips were rinsed with DPBS containing 50 mM trehalose for 10 min before being completely dried with nitrogen and stored in the dark. Dried PillarChips were imaged using a Thermo Scientific Cellomics ArrayScan VTI high-content system. Control cell spots incubated solely with fluorescently labeled secondary antibodies yielded low, non-specific background signals that were subtracted during analysis.

### Western Blotting

Protein concentration of lysates was estimated using a BCA assay (Sigma). Equal amounts of protein (∼10 μg) were loaded into either 8% or 12% cast SDS-PAGE gels. Gels were resolved at 120 V in a Tris-glycine buffer and electrotransferred to a nitrocellulose membrane for either 60 min at 100 V (12% gel) or overnight at 30 V (8% gel) at 4°C. Membranes were rinsed with Tris-buffered saline (TBS), blocked in 5% (w/v) BSA TBS for 1 hr at room temperature, and incubated overnight at 4°C in primary antibody solutions diluted in blocking buffer with 0.05% (v/v) Tween 20. Membranes were rinsed with TBS containing 0.05% Tween 20 (TBST) and incubated with an appropriate secondary antibody diluted in TBS with 3% BSA for 90 min at room temperature. Membranes were washed with TBST and then TBS before incubation with chemiluminescence solution (SuperSignal Pico, Pierce) for 10 min. Membranes were imaged with a Bio-Rad ChemiDoc and subsequently stripped using a stripping buffer (200 mM glycine, 1% [v/v] Tween 20, 0.1% [w/v] SDS [pH 2]) and evaluated for efficient stripping with chemiluminescence before being reused with the above procedure for detection of loading control proteins (GAPDH for 12% gels, vinculin for 8% gels).

### On-Chip Cytotoxicity Assay

Undifferentiated and differentiated microchip cultures were prepared as described above. Chemicals were purchased from Sigma or the NIH Small Molecule Repository (Table S2). Concentrated stocks were prepared and diluted into ReNcell Maintenance Medium to prepare the highest screened concentration solution for each compound. Four-fold serial dilutions were made in ReNcell Maintenance Medium, with all solutions having a final concentration of 0.5% or 1% DMSO (v/v) and 20 μg/mL Matrigel. For undifferentiated cultures, the solutions also contained 20 ng/mL of EGF and FGF2. These solutions were printed into WellChips, covered with a gas-permeable membrane, and warmed at 37°C for at least 5 min before stamping with cells. Each WellChip contained six compounds each at six concentrations (n = 10 biological replicates for each concentration) and the appropriate medium containing 0.5% or 1% DMSO for vehicle and dead controls (n = 32 biological replicates per control). PillarChip cultures were incubated with compounds for 72 hr with daily medium exchange to freshly prepared WellChips containing compounds at the appropriate concentrations. After 72 hr, PillarChips were assayed with the viability assay and when appropriate data were fit to a sigmoidal dose-response model using GraphPad Prism for IC_50_ calculation (see Supplemental Information). On-chip toxicity screens were repeated three times for undifferentiated and differentiated cultures.

### On-Chip Proliferation Assay

Undifferentiated and differentiating cultures were screened for toxicity as described previously, except that after the first 48 hr of exposure 10 μM EdU (Click-iT EdU, Invitrogen) was added to the compound-containing medium for the final 24 hr. After 72 hr, on-chip cultures were fixed and processed as described previously for on-chip immunofluorescence; however, instead of antibody staining the cultures were incubated with solution containing Alexa Fluor 488-azide for 30 min as specified in the product manual. Cultures were then rinsed with DPBS for 30 min, incubated in DPBS with Hoechst 33342 (5 μg/mL) for 30 min, and washed in DPBS for 30 min before proceeding with a trehalose wash and drying as described previously. Nuclei were selected using Cellomics software from blue channel images to create a mask, which was used for identification of fluorescently labeled EdU in the green channel for quantification of actively proliferating cells over the 24-hr EdU exposure. Data were fit to a sigmoidal dose-response model using GraphPad Prism to calculate IC_50_ values.

## Author Contributions

J.S.D. and D.V.S. conceived the project. J.S.D., D.S.C., and D.V.S. supervised the studies. G.J.N., S.K.M., B.C.P., and J.F.P. designed experiments. G.J.N. performed experiments and analysed data. G.J.N. and J.S.D. wrote the manuscript. All authors critically revised the manuscript.

## Figures and Tables

**Table 1:** Differential Acute Toxicity Effects on Viability and Proliferation of Undifferentiated and Differentiating ReNcell VM NPCs

Chemical	Viability Effects				Proliferation Effects			
	Undifferentiated		Differentiating		Undifferentiating		Differentiating	
	log(IC_50_ ± SEM)	IC_50_ (μM)	log(IC_50_ ± SEM)	IC_50_ (μM)	log(IC_50_ ± SEM)	IC_50_ (μM)	log(IC_50_ ± SEM)	IC_50_ (μM)
**Acetaminophen**^a^	3.1 ± 0.21	1,400	4.3 ± 0.24	18,000	3.017 ± 0.05	1,000	4.26 ± 0.52	18,200
**5-Fluorouracil**	0.36 ± 0.31	2.3	0.35 ± 0.22^b^	2.3	0.14 ± 0.10	1.35	0.26 ± 0.40	1.82
Cytosine Arabinoside^c^	−0.48 ± 0.17^b^	0.33	0.55 ± 0.55	3.5	< −1	–	< −1	–
**Retinoic Acid**^a^	−0.13 ± 0.21	0.74	1.7 ± 1.2	47	1.17 ± 0.11	15	1.35 ± 0.26	22
**Doxorubicin**^a^	−0.1 ± 0.11	0.8	0.44 ± 0.06	2.7	< −1	–	< −1	–
Cadmium Chloride	0.57 ± 0.3	3.7	0.88 ± 0.27	7.6	0.93	8.6	1.18	15
Paraquat	1.8 ± 0.18	57	1.61 ± 0.18	41	1.77 ± 0.09	59	1.48 ± 0.64	30
**Pitavistatin**	0.73 ± 0.16	5.4	–	–	0.29 ± 0.07	2.0	−0.01 ± 0.39	0.98
Diphenhydramine	2.41 ± 0.37	257	2.3 ± 0.24	189	–	–	–	–

Extracted IC_50_ values from sigmoidal fits of dose-response viability and proliferation screening displayed as log(IC_50_ ± SEM [μM]) and corresponding IC_50_ value (μM). Chemicals which elicited differential effects on undifferentiated and differentiating NPCs are highlighted in bold. Results are based on dose-response data from which each dose had 30 biological replicates unless indicated. Dose-response data not amenable to sigmoidal fitting are represented as a dash. Unlisted screened compounds were omitted because the dose-response screen did not capture the effective window, making them unfit for sigmoidal curve fitting.

a p < 0.05 (two-tailed Student's t test).

b Results based on data from which each dose had 20 biological replicates.

c p < 0.10 (two-tailed Student's t test) difference between IC_50_ of undifferentiated and differentiating NPCs.

## Supplemental Information

Document S1. Supplemental Experimental Procedures, Figures S1–S5, and Tables S1–S3

Document S2. Article plus Supplemental Information
