# Supplementary material for: High-Throughput Toxicity and Phenotypic Screening of 3D Human Neural Progenitor Cell Cultures on a Microarray Chip Platform
Source: Stem Cell Reports. 2016 Oct 27;7(5):970–82. doi: 10.1016/j.stemcr.2016.10.001 (PMC5106528; doi:10.1016/j.stemcr.2016.10.001)
Supplement: Document S1. Supplemental Experimental Procedures, Figures S1–S5, and Tables S1–S3 [file mmc1.pdf]

Stem Cell Reports, Volume 7

Supplemental Information

High-Throughput Toxicity and Phenotypic Screening of 3D Human Neural Progenitor Cell Cultures on a Microarray Chip Platform

Gregory J. Nierode, Brian C. Perea, Sean K. McFarland, Jorge F. Pascoal, Douglas S. Clark, David V. Schaffer, and Jonathan S. Dordick

Supplemental Information

for

High-Throughput Toxicity and Phenotypic Screening of 3D Human Neural Progenitor Cell Cultures on a Microarray Chip Platform

Supplemental Figures and Tables

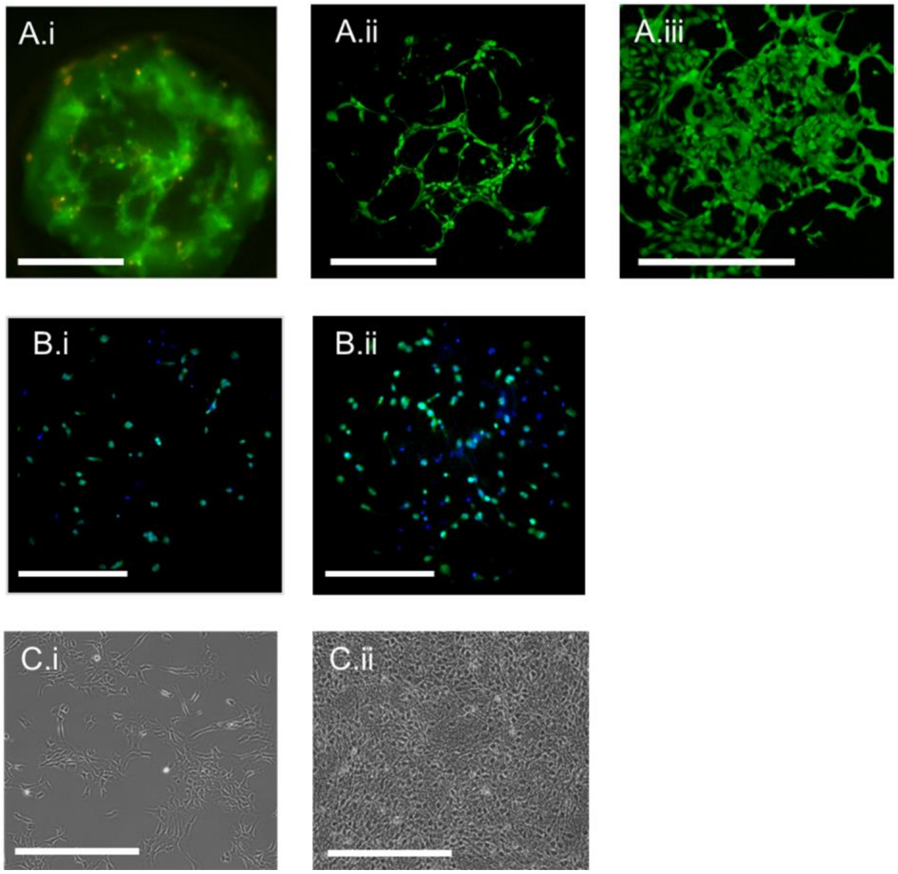

Figure S1 (related to Figure 2). ReNcell VM morphology and preservation of cytoplasmic calcein in dried 3D microarray spots. (a) Compiled fluorescent images (488 and 549 filters) of a NPC-Matrigel 3D cell spot stained with calcein and ethidium homodimer (i) when dried without trehalose, where cells lysed during dessication and entrapped calcein diffused throughout the individual cell spot during the drying process, (ii) when dried with trehalose, where cells retain cytoplasmic staining of calcein, and (iii) when imaged immediately following staining while kept in DPBS, which demonstrates the localization of calcein stain in a living, hydrated sample. (b) Post-printing viability after preparation of on-chip ReNcell VM NPC microscale cultures. After 1 day of culture, chips were stained with calcein and Hoechst 33342 stains and imaged and analyzed using Cellomics software to determine the post-printing viability in (i) 0.5% and (ii) 1% (w/v) Matrigel. Nuclei were detected and used to generate a mask of selected objects, and each object was assessed for detection of calcein staining to identify living cells. Viability was determined to be 84 ± 5% and 80 ± 6% (mean ± SD, n = 396 biological replicates) in 0.5% and 1% Matrigel, respectively. (c) Phase contrast images depicting ReNcell VM when cultured (i) with and (ii) without EGF and FGF2 in 2D monolayer cultures. Scale bars = 300 μm.

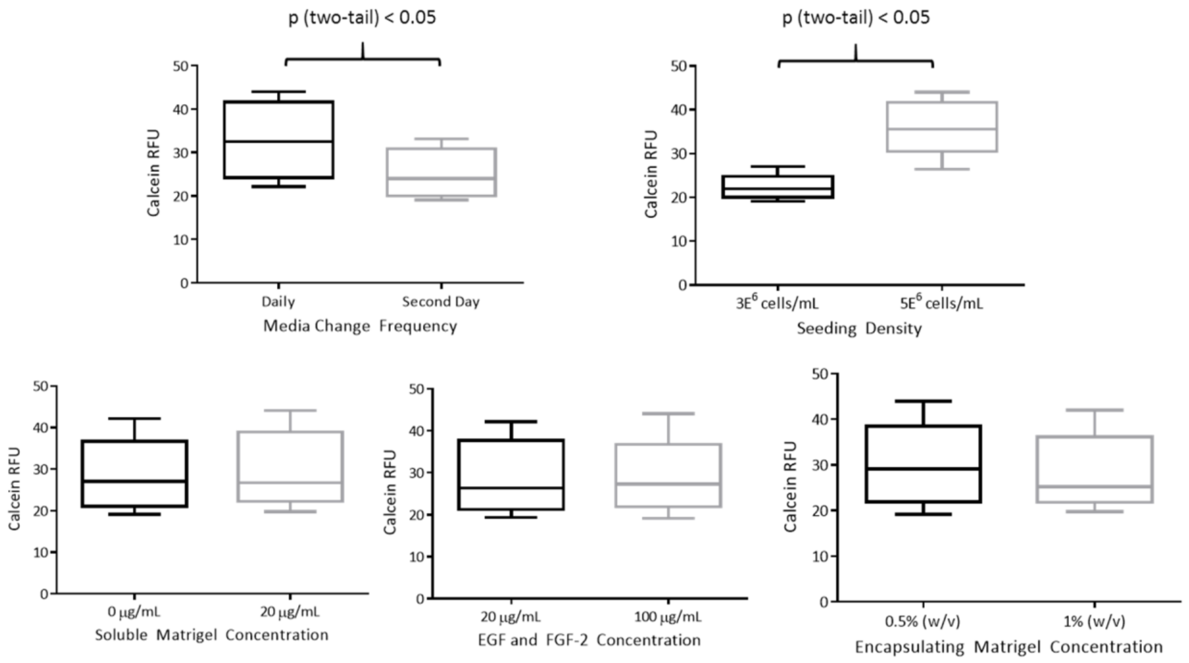

Figure S2 (related to Figure 2). Factorial design screen to identify NPC culture conditions on-chip. Five culture conditions were screened at two levels in a 2^5 factorial design experiment to identify factors influencing NPC growth on-chip. Average calcein fluorescence for each spot pertaining to each condition was entered into GraphPad Prism (24 independent biological replicates per condition) to generate Tukey box plots and perform student t-tests (two-tail). As expected, higher seeding density resulted in a higher calcein fluorescence intensity ($p < 0.05$). Media change frequency had a substantial and significant ($p < 0.05$) impact on growth and viability NPCs. Presence of Matrigel in media, growth factor concentration and encapsulating Matrigel concentration were not significantly influential on viability and growth.

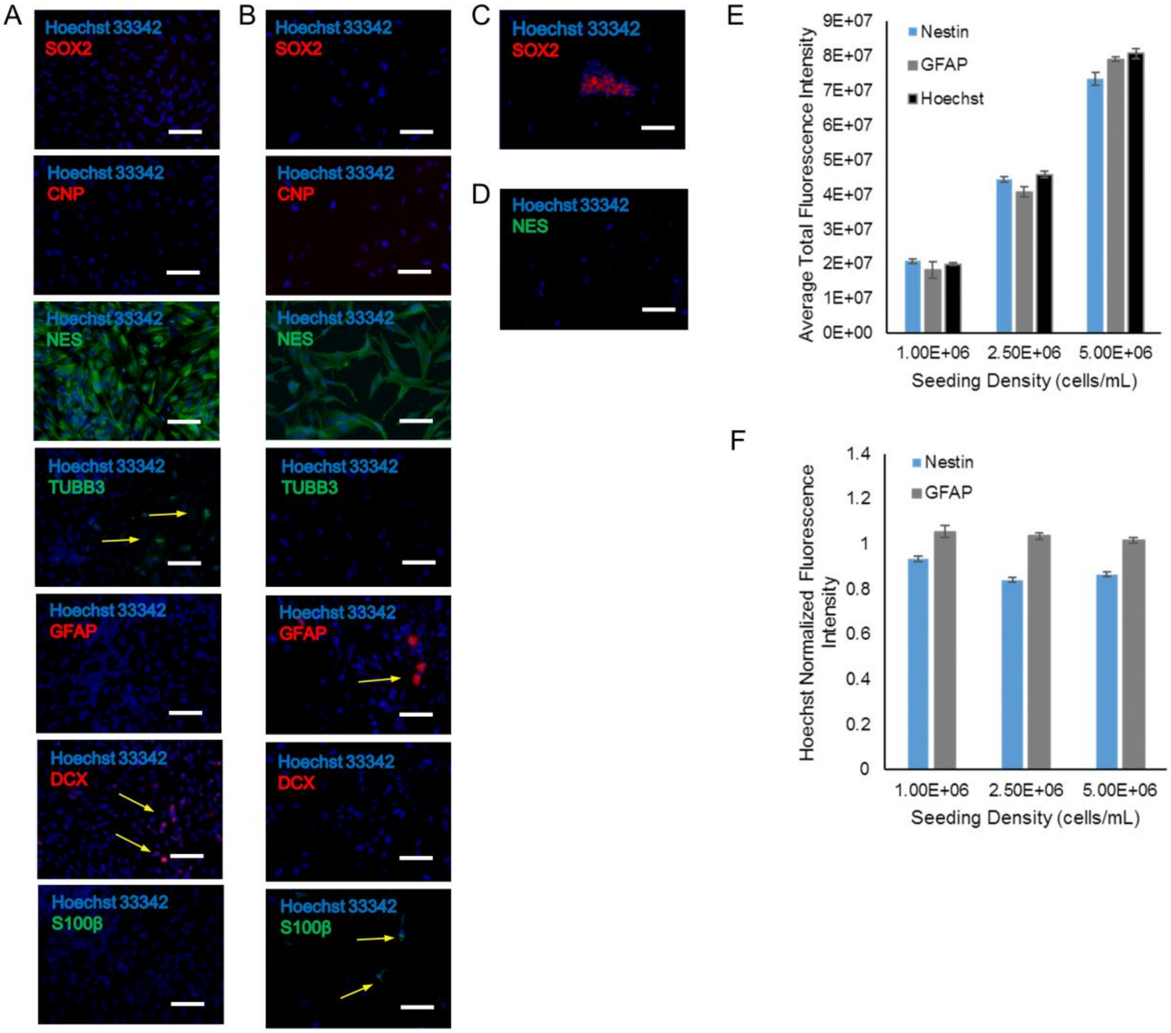

Figure S3 (related to Figure 3 and Figure 4). Immunofluorescence primary antibody validation and on-chip immunofluorescence assay sensitivity. Merged immunofluorescence images of (a) differentiated SK-N-SH neuroblastoma cells (b) CCF-STTG1 astrocytoma cells (c) human embryonic stem cells and (d) human mesenchymal stem cells incubated with antibodies specific for the indicated protein and Hoechst 33342 to counterstain nuclei. The merged images were processed in ImageJ by first adjusting brightness and contrast to eliminate background fluorescence using samples incubated only with the appropriate fluorescent secondary antibody. These single-channel images were then merged with the blue channel to merge with the stained nuclei, with the exception being the image depicted in (c). The blue channel was to generate a mask of nuclei that could be overlaid with the labelled protein channel since SOX2 and nuclei should co-localize and this makes for simplified confirmation of the proper subcellular location. As expected, the neuroblastoma cells in (a) were negative for SOX2 and glial protein markers. Also, the majority of cells were positive for NES. A small population of differentiated cells were positive for TUBB and DCX (indicated with yellow arrows), which together demonstrated the specificity of TUBB3, DCX and NES antibodies on neuronal populations. Notably, the differentiation of the neuroblastoma line yielded a heterogeneous mixture of differentiated and undifferentiated neurons, making it a good choice for validation of the neuronal specific antibodies. The astrocytoma cells in (b) were negative for SOX2 and neuronal protein markers. The majority of cells were also positive for NES. A small population of differentiated cells were positive for GFAP and S100β (indicated with yellow arrows), which together demonstrated the specificity of GFAP, S100β, and NES antibodies. The chosen cell line is a late stage glioma, which does not express high levels of astrocytic protein markers (i.e. GFAP, S100β), making it a good choice for screening specificity. Additionally, the human embryonic stem cells (c) were positive for Sox-2 and the human mesenchymal stem cells (d) were negative for NES, further demonstrating the specificity of these

antibodies. Scale bars = 100 µm. (e) To assess on-chip immunofluorescence assay sensitivity, a microarray chip was prepared with varied cell densities (1, 2.5 and 5 x 10^6 cells/mL) for detection of NES or GFAP expression with an on-chip immunofluorescence. The total fluorescence intensity was measured for an entire cell spot stained for either GFAP or NES and averaged (n = 54 biological replicates for each) to plot the mean ± SEM against seeding density. (f) Dividing the immunofluorescence intensity by the Hoechst 33342 intensity (nuclear stain) within each cell spot resulted in immunofluorescence signals normalized by the number of cells present within each spot. These normalized values were averaged (n = 54 biological replicates for each) for GFAP and NES to plot normalized intensity ± SEM against seeding density.

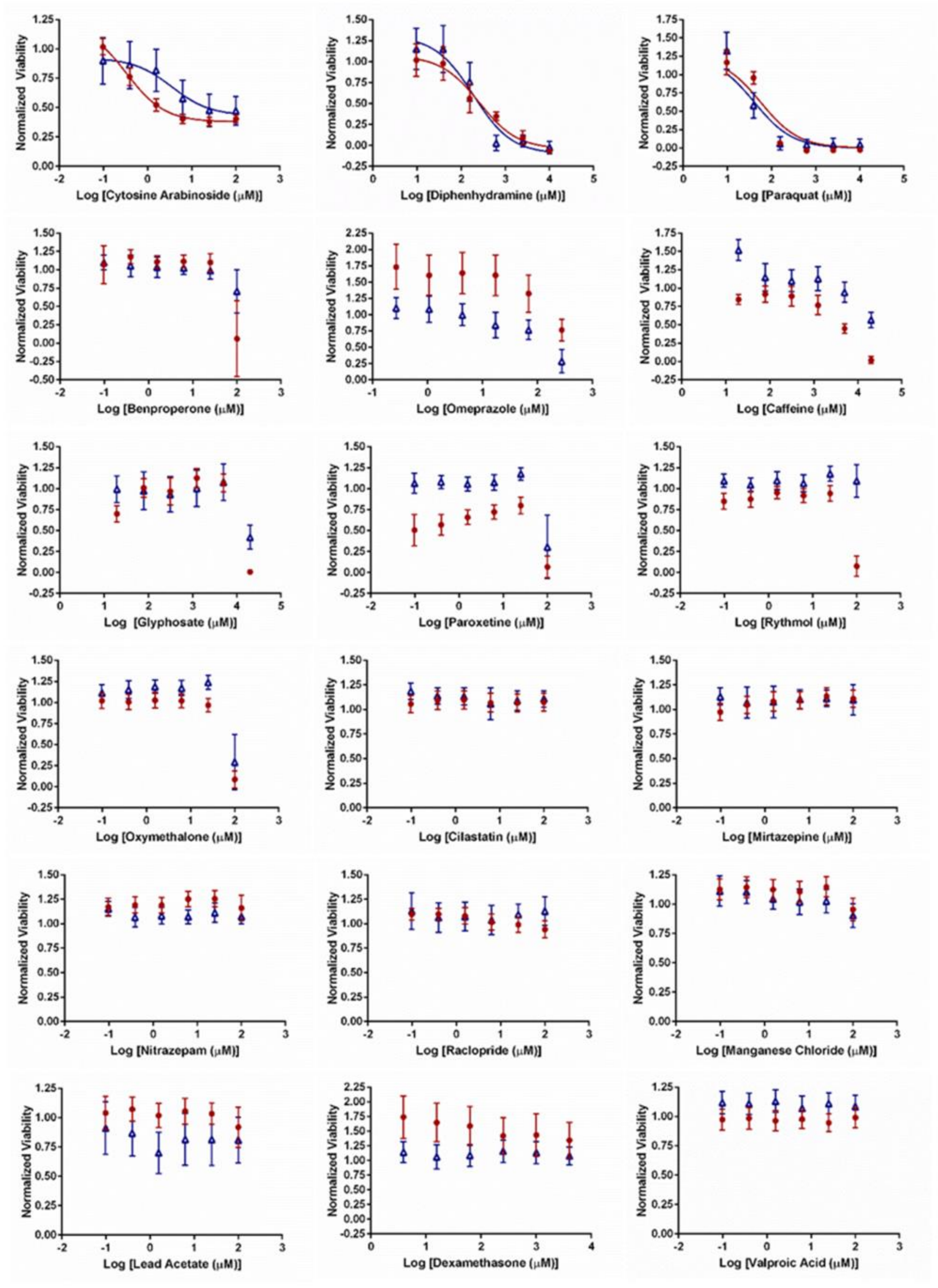

Figure S4 (related to Figure 5 and Table 1). Dose-response viability curves of undifferentiated and differentiating NPCs. Vehicle control normalized dose-response viability of undifferentiated (●) and differentiating (**Δ**) NPCs plotted against log(concentration (µM)). Mean viability ± SEM is plotted for each concentration as determined from 30 biological replicates per dose. Exceptions for this include undifferentiated raclopride (10 biological replicates), differentiated caffeine (20 biological replicates) and undifferentiated cytosine arabinoside (20 biological replicates). Sigmoidal fits to the data are plotted as solid lines when appropriate.

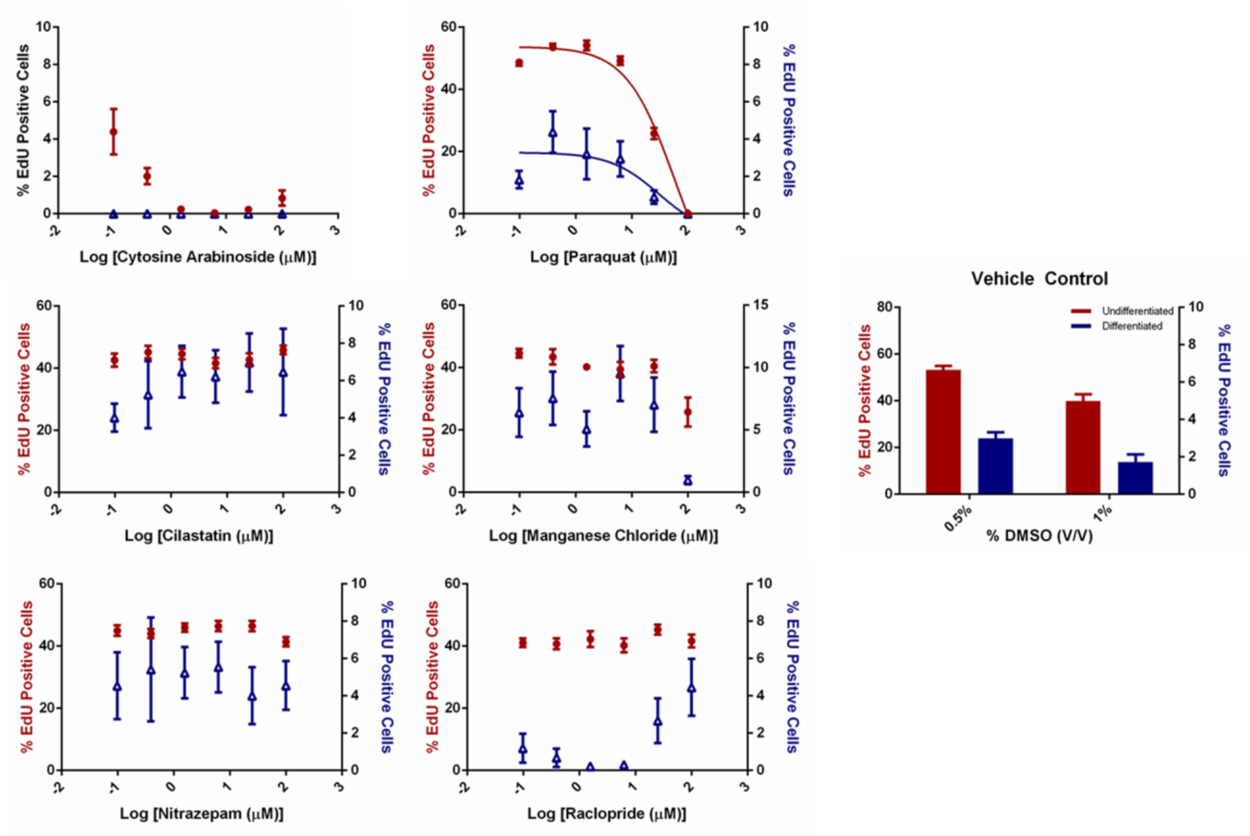

Figure S5 (related to Figure 5, Table 1). Dose-response proliferation curves of undifferentiated and differentiating NPCs. Dose-response EdU incorporation of undifferentiated (●, left axis) and differentiating (**Δ**, right axis except doxorubicin) NPCs plotted against log(concentration (μM)). Mean % EdU positive nuclei ± SEM is plotted for each concentration as determined from 10 biological replicates.

Antibody	Vendor	Application	Dilution
Chicken monoclonal anti-CNPase	Abcam	IF	1:100
Rabbit polyclonal anti-doublecortin	Abcam	IF, WB	1:250 (IF), 1:1000 (WB)
Rat monoclonal anti-GFAP	Invitrogen	IF	1:250
Mouse monoclonal anti-Nestin	Abcam	IF, WB	1:250 (IF), 1:1000 (WB)
Mouse monoclonal anti-βIII tubulin	Abcam	IF, WB	1:250 (IF), 1:1000 (WB)
Rabbit monoclonal anti-Sox2	Abcam	IF, WB	1:100 (IF), 1:1000 (WB)
Mouse monoclonal anti- S100β	Abcam	IF, WB	1:100 (IF), 1:500 (WB)
Mouse monoclonal anti-GFAP	Abcam	WB	1:250
Rabbit monoclonal anti-CNPase	Cell Signaling Tech	WB	1:1000
Mouse monoclonal anti-Vinculin	Sigma	WB	1:200
Mouse monoclonal anti-GAPDH	Abcam	WB	1:500
Rabbit monoclonal anti-Sox2	Cell Signaling Tech	WB	1:1000
Goat anti-mouse Alexa 488	Invitrogen	IF	1:100
Goat anti-rat Alexa 549	Invitrogen	IF	1:100
Goat anti-rabbit Alexa 549	Invitrogen	IF	1:100
Goat anti-chicken Alexa 549	Invitrogen	IF	1:100
Goat anti-mouse Horseradish Peroxidase	Invitrogen	WB	1:5000
Goat anti-rabbit Horseradish Peroxidase	Invitrogen	WB	1:5000

Table S1 (related to experimental methods). Compiled table of antibodies used in this work**.** WB = Western Blot; IF = immunofluorescence

Chemical	Chemical classification	Concentration range screened (µM)	Vendor	Vehicle (% DMSO)
Acetaminophen	analgesic	80 – 80,000	Sigma	0.5
Raclopride	anti-psychotic	0.1 – 100	NIH Clinical Collection	1
Nitrazepam	anti-anxyletic	0.1 – 100	NIH Clinical Collection	1
5-Fluorouracil	chemotherapeutic	0.1 – 100	Sigma	0.5
Cytosine Arabinoside	chemotherapeutic	0.1 – 100	Sigma	0.5
Retinoic Acid	chemotherapeutic	0.1 – 100	Sigma	0.5
Doxorubicin	chemotherapeutic	0.1 – 100	Sigma	0.5
Cadmium Chloride	heavy metal	0.1 – 100	Sigma	0.5
Manganese Chloride	heavy metal	0.1 – 100	Sigma	0.5
Cilastatin	statin	0.1 – 100	NIH Clinical Collection	1
Pitavistatin	statin	0.1 – 100	NIH Clinical Collection	1
Diphenhydramine	anti-histamine	10 – 10,000	Sigma	0.5
Dexamethasone	steroid	4 – 4000	Sigma	0.5
Oxymethalone	steroid	0.1 – 100	NIH Clinical Collection	1
Caffeine	stimulant	20 – 20,000	Sigma	0.5
Rythmol	anti-arrhythmic	0.1 – 100	NIH Clinical Collection	1
Paroxetine	anti-depressant	0.1 – 100	NIH Clinical Collection	1
Mirtazapine	anti-depressant	0.1 – 100	NIH Clinical Collection	1
Valproic Acid	anti-epileptic	0.1 – 100	NIH Clinical Collection	1
Benproperine	anti-tussive	0.1 – 100	NIH Clinical Collection	1
Lead Acetate	heavy metal	0.1 – 100	Sigma	0.5
Glyphosate	herbicide	20 – 20,000	Sigma	0.5
Omeprazole	proton pump inhibitor	0.1 – 100	Sigma	0.5
Paraquat	herbicide	10 – 10,000 (viability)<br>0.1 – 100 (proliferation)	Sigma	0.5

Table S2 (related to experimental methods, Figure 5 and Table 1). List of chemicals screened for viability and proliferation effects with the concentration range screened, vendor, and vehicle control used.

Chemical	Normalized viability at C_{max}	
	Undifferentiated Mean ± SEM	Differentiating Mean ± SEM
Lead Acetate	0.92 ± 0.17	0.81 ± 0.19
Glyphosate	0.01 ± 0.03	0.41 ± 0.14
Cytosine Arabinoside	0.40 ± 0.03[a]	0.47 ± 0.12
Paraquat	-0.02 ± 0.03	0.05 ± 0.02
Diphenhydramine	-0.07 ± 0.02	-0.02 ± 0.08
Caffeine	0.02 ± 0.04	0.56 ± 0.10[a]
Retinoic Acid	0.55 ± 0.16	0.50 ± 0.13
Omeprazole	0.76 ± 0.17	0.29 ± 0.18
Dexamethasone	1.35 ± 0.33	1.08 ± 0.16
Cadmium Chloride	0.06 ± 0.07	0.09 ± 0.04
5-Fluorouracil	0.15 ± 0.10	0.81 ± 0.17
Acetaminophen	-0.16 ± 0.07	-0.1 ± 0.03
Manganese Chloride	0.96 ± 0.10	0.90 ± 0.10
Cilastatin	1.07 ± 0.10	1.11 ± 0.08
Oxymethalone	0.09 ± 0.10	0.29 ± 0.33
Valproic Acid	0.99 ± 0.08	1.09 ± 0.10
Pitavastatin	0.14 ± 0.11	1.16 ± 0.07
Raclopride	0.94 ± 0.08[b]	1.13 ± 0.15
Benproperone	0.06 ± 0.52	0.71 ± 0.30
Nitrazepam	1.16 ± 0.13	1.07 ± 0.07
Mitrazepine	1.11 ± 0.09	1.10 ± 0.15
Rythmol	0.08 ± 0.12	1.09 ± 0.20
Paroxetine	0.07 ± 0.13	0.30 ± 0.38
Doxorubicin	0.05 ± 0.13	0.11 ± 0.16

Table S3 (related to Figure 5 and Table 1). Vehicle-control normalized viability of undifferentiated and differentiating human NPCs at the maximum concentration tested (see Table S2). The data represent the normalized mean and SEM of 30 biological replicates. Chemicals in bold were deemed to be toxic because they exhibited ≥50% reduction in viability of either undifferentiated or differentiating cultures. [a]denotes a condition where the value is from 20 biological replicates. [b]denotes a condition where the value is from 10 biological replicates.

Supplemental Experimental Procedures

Cell culture of cell lines used in validation of primary antibodies

The neuroblastoma line SK-N-SH (ATCC) was cultured per manufacturer's recommendations. Specifically, the cells were cultured using Eagle's Minimum Essential Media (EMEM) supplemented with 10% fetal bovine serum (Sigma Aldrich). Cells were cultured in T75 flasks at 5% CO_2 and 37 °C. Media was exchanged twice weekly. Cells were passaged by rinsing with DPBS, incubating with trypsin-EDTA (Gibco) and suspended in EMEM media before centrifugation at 300 x g and resuspension in fresh medium. Differentiation of SK-N-SH was carried out as described by Jain et al. (Jain et al., 2007). Briefly, the cells were plated and cultured in a flask with media containing all-*trans* retinoic acid (RA, 10 µM) for one week. Following, the cells were re-passaged onto PLL-coated 96-well plates and cultured with EMEM containing 5% fetal bovine serum, 10 µM RA, and 1 µM cytosine arabinoside (to reduce proliferation of undifferentiated cells) for one week, at which time the cells were fixed for immunostaining. Cells were at passage number 5 or less upon use.

The astrocytoma line CCF-STTG1 (ATCC) was cultured per manufacturer's recommendations. Briefly, the cells were cultured with RPMI media supplemented with 10% fetal bovine serum. Cells were cultured in T75 flasks at 5% CO_2 and 37 °C. Media was exchanged every second day. Cells were passaged by rinsing with DPBS, incubating with trypsin-EDTA (Gibco) and suspended in EMEM media before centrifugation at 300 x g and resuspension in fresh medium. For primary antibody validation, cells were seeded to a 96-well plate and cultured overnight prior to fixation. Cells were at passage number 5 or less upon use.

The human embryonic stem cell line (hESC; RUES2) was provided as a 96-well plate of cells at passage 32, kindly donated by Dr. Brigitte Arduini of the Rensselaer Center for Stem Cell Research (RCSCR). Before fixation, the cells were maintained in mouse embryonic fibroblast-conditioned medium (MEF-CM) produced by the RCSCR supplemented with FGF2 (20 ng/mL; Invitrogen), cultured on Matrigel-coated 35 mm and 65 mm plates at 5% CO_2 and 37 °C. The media was changed daily and cells routinely passaged in clumps using dispase (Stem Cell Technologies). Upon receipt of the 96-well plate, the cells were immediately fixed and processed for immunostaining.

The human embryonic mesenchymal stem cells (hMSC; Lonza) were cultured according to the manufacturer's instructions with slight modifications. Maintenance medium consisted of DMEM supplemented with 10% fetal bovine serum (MSC qualified; Gibco) and medium was changed every second day. The cells were passaged with TrypLE (Gibco) after rinsing with DPBS upon reaching high confluence. The cells were cultured on T25 flasks and stored at 5% CO_2 and 37 °C. Cells were at passage number 10 or less when used. All immunofluorescence protocols used were identical to those used on ReNcell VM NPCs.

Confocal imaging of NPC in Matrigel

On-chip ReNcell VM cultures in either 0.5% or 1% Matrigel (~300 cells/100 nL spot) were seeded as described above and cultured for three days with daily media change. On the third day, cultures were stained with the viability assay, but the chips were kept in DPBS containing 1 g/L glucose instead of being dried. The stained chips were imaged using a Zeiss LSM multiphoton confocal fluorescence microscope. The first imaged plane was the top of the gel, which was found by adjusting focus until no more cells were observed. Images were taken every 10 µm toward the bottom of the cell spot. The bottom of the gel was determined when no more cells were observed. Z-stacks were constructed using ImageJ.

Factorial analysis of growth conditions

A factorial design experiment was performed to assess simultaneously multiple culture conditions. Two values of five different variables were screened for a total of 32 conditions tested in parallel with n=24 replicates. The conditions tested were media change frequency (daily or every second day), growth factor concentration (20 ng/mL or 40 ng/mL), Matrigel in the media (none or 20 µg/mL), seeding density (300 cells/spot or 500 cells/spot) and the concentration of encapsulating Matrigel (0.5% (w/v) or 1% (w/v)). On-chip cultures were prepared to accordingly to the screened conditions, and after five days of culture on-chip the chips were used with the viability assay. The average calcein fluorescence of each spot was measured and grouped for each condition tested for entry into GraphPad Prism 6.0 for generation of Tukey box plots to assess the overall impact of each condition. Two-tailed student's t-tests were performed to assess statistical significance for each factor tested.

Determination of after-printing viability

The day one PillarChip from the growth experiment was used in determining viability of NPCs in 1% (w/v) Matrigel one day after printing. Cellomics software was used to detect and select all nuclei within a cell spot by gating for regions of strong fluorescence. The detected nuclei were used as a mask in the green channel to determine whether green fluorescence was detected within the same region. If green staining overlaid with blue staining (thus, calcein staining and Hoechst 33342 staining was co-localized), the object was counted as a living cell. The percent viability was determined by dividing the number of living cells (co-localized green and blue stains) by the total number of detected nuclei and multiplying by 100. The average and standard deviation were determined from n=396 replicates from a single printed chip for each 0.5% and 1% Matrigel conditions.

In-cell immunofluorescence imaging for monolayer cultures and antibody validation

Samples processed for immunofluorescence analysis of 2D monolayer cultures and primary antibody validation were prepared as described previously. The samples were imaged using an IX51 Olympus microscope with filters for DAPI, FITC and Texas Red. Images taken with the DAPI filter were exposed for 5 ms. Images taken with the FITC and Texas Red filters were exposed for 500 ms. Exceptions to this were differentiated ReNcell VM cells images for GFAP. The GFAP intensity was so high on the differentiated cultures that samples had to be imaged with a 15 ms exposure time. Images were transported to ImageJ for processing and compilation.

Cellomics high-content analysis

For viability assay samples, images were acquired at both 50X and 100X magnification with a multiple bandpass emission and excitation filters (BGRFR) in blue (386), green (485) and red (549) channels using 2.5 millisecond exposures for the blue and green channels and a 15 ms exposure for the red channel. Images from 50X magnification were used to measure the total fluorescence in the green channel, which was normalized by the total number of pixels within the image (constant for all measurements) to determine calcein staining intensity. The total fluorescence in the blue channel measured for Hoechst 33342 fluorescence. Red fluorescence from ethidium homodimer-I was not used in analysis, and was therefore not quantified. Images from 100X magnification were used only for determination of after-printing viability.

For on-chip, in-cell immunofluorescence assay samples, images were acquired at 100X magnification with a multiple bandpass emission filter and excitation filters (BGRFR). Images were collected for blue (386), green (485), red (549) and far-red (650) channels using exposure times of 2, 30, 75, and 50 ms, respectively. The red (549) channel was also captured with a 7.5 ms exposure for GFAP analysis, which had significantly higher staining intensities. Within each cell spot, the total fluorescence in each channel measured using the Cellomics analytical software. This measured intensity was normalized by the total intensity from the blue channel to account for cell number within each spot. The normalized non-specific signal from the appropriate fluorescent secondary antibody was subtracted from the normalized intensities associated with each protein marker to account for background staining. These values were then plotted for assessing the expression levels of protein markers. The background subtracted, normalized fluorescence intensities were averaged over n = 27 spots for each protein assessed, omitting detached cell spots from analysis. Student's t-tests (two-tailed) were done for statistical analysis in comparing these corrected mean intensities between undifferentiated and differentiated samples.

Growth analysis and viability assay sensitivity of on-chip 3D hNPC cultures

Four NPC PillarChip cultures were prepared as described previously in 0.5% and 1% (w/v) Matrigel and cultured in WellChips containing complete growth media with daily media change. After one, three, five, and seven days of culture on-chip, an entire PillarChip was assayed using the live/dead viability assay. The background calcein fluorescence from dead cell control spots (n=36) was averaged and subtracted from the average calcein fluorescence intensity from living cell spots (396 replicates). Detached cell spots were omitted from analysis (< 5% of the total cell spots). The adjusted average calcein fluorescence intensity for each time point (Eq. 1) was used in generating growth curves.

$$F_{calcein} = \bar{F}_{485,\ sample} - \bar{F}_{485,\ dead\ control} \quad (1)$$

For the viability sensitivity assay, a PillarChip was prepared with 100 nL spots of Matrigel-cell suspension of either 0.5% or 1% Matrigel at densities of 0, 1 x 10^6, 2.5 x 10^6, 5 x 10^6, 7.5 x 10^6, and 10 x 10^6 cells/mL and stamped into a WellChip for culture overnight. The following day, chips were stained with the viability assay. The

pixel-normalized calcein fluorescence was adjusted for background staining as described in Eq. 1, however instead of the background fluorescence from dead cells the green fluorescence intensity from the 0 cells/mL condition was used to account for background staining. This background-adjusted condition was averaged (n=72) and plotted against seeding density. The total Hoechst 33342 fluorescence was averaged over each condition (n=72) and the total fluorescence from the 0 cells/mL condition was subtracted to account for background fluorescence and plotted against seeding density.

Western blot sample preparation

For 3D Western blot analysis, ReNcell VM cells were seeded at 890,000 cells/mL in 1% (w/v) Matrigel in 8-well chamber microscope slides (Nunc). Cell suspension (100 µL) was placed into each chamber and allowed to gel for 20 min at 37 °C. After gelation, 250 µL of medium was added to each well and media was changed daily. Cell lysate was collected for undifferentiated cultures after three days of culture in complete growth media. Differentiated cell lysate was collected after five days of growth in complete growth media followed by a 10-day differentiation in differentiation media. Prior to collection of lysates, 3D cultures were rinsed once with ice cold PBS before 2 h incubation with a Cell Recovery solution (BD) at 4 °C for Matrigel depolymerization. The cell suspension was centrifuged at 300 g for 5 min at 4 °C and cells were re-suspended in cold Cell Recovery solution. The suspension was centrifuged again and re-suspended in cold PBS prior to a third centrifugation. The pellet was lysed in 250 µL RIPA buffer containing protease inhibitor cocktail (Gold Biotechnology) and stored at -20 °C. For 2D Western blot analysis, ReNcell VM cells were seeded at 100,000 cells/well in complete growth media into laminin-coated 35 mm dishes. Media was changed daily, and once 100% confluence was reached, lysate was collected for the undifferentiated sample and media was changed to differentiation media for the differentiated sample. Media was changed daily for the differentiation sample for 10 days, at which time lysate was collected. The 2D cultures were rinsed once with ice cold PBS prior to lysate collection in 250 µL RIPA buffer containing protease inhibitor cocktail, after which samples were stored at -20 °C.

On-chip toxicity assay analysis

The average adjusted calcein fluorescence for condition was determined as described above (Eq. 1). The vehicle control adjusted calcein fluorescence was determined in a similar manner (Eq. 2), and the normalized viability was determined by dividing the calcein fluorescence of a sample by the calcein fluorescence of a vehicle control (Eq. 2 and Eq. 3). Detached cell spots and cell spots with intensities larger than two standard deviations from the mean of each condition were excluded from analysis.

$$F_{vehicle\ control\ \ calcein} = \bar{F}_{485,\ \ vehicle\ control} - \bar{\bar{F}}_{485,\ \ dead\ control} \qquad (2)$$

$$Normalized\ Viability = \frac{F_{sample\ calcein}}{F_{vehicle\ control\ calcein}} \qquad (3)$$

These averaged viabilities and their associated standard errors of the mean were used with GraphPad Prism 6.0 (GraphPad Software) to fit sigmoidal dose response curves with constant slopes using Eq 4. For dose-response curves without clearly defined top or bottom plateaus, these values were set to either the maximum or minimum viability values within the sampled range (e.g. “Top” constraint for paraquat fit set to 1.25 and “Bottom” constraint for retinoic acid fit set to “0”). The log(IC_{50}) values were obtained from the dose response curves generated in GraphPad Prism 6.0. Student’s t-tests (two-tail) were performed for statistical analysis on the log(IC_{50}) values.

$$Y = Bottom + \frac{(Top - Bottom)}{(1 + 10^{\log EC50 - X})} \qquad (4)$$

Supplemental References

Jain, P., Cerone, M.A., LeBlanc, A.C., Autexier, C., 2007. Telomerase and neuronal marker status of differentiated NT2 and SK-N-SH human neuronal cells and primary human neurons. J. Neurosci. Res. 85, 83–89. doi:10.1002/jnr.21094
