# Supplementary material for: High-Throughput Toxicity and Phenotypic Screening of 3D Human Neural Progenitor Cell Cultures on a Microarray Chip Platform
Source: Stem Cell Reports. 2016 Oct 27;7(5):970–82. doi: 10.1016/j.stemcr.2016.10.001 (PMC5106528; doi:10.1016/j.stemcr.2016.10.001)
Supplement: Document S2. Article plus Supplemental Information [file mmc2.pdf]

Stem Cell Reports
Article

OPEN ACCESS

High-Throughput Toxicity and Phenotypic Screening of 3D Human Neural Progenitor Cell Cultures on a Microarray Chip Platform

Gregory J. Nierode,[1] Brian C. Perea,[2] Sean K. McFarland,[3] Jorge F. Pascoal,[4] Douglas S. Clark,[2] David V. Schaffer,[2,3] and Jonathan S. Dordick[1,*]

[1]Chemical and Biological Engineering and Center for Biotechnology & Interdisciplinary Studies, Rensselaer Polytechnic Institute, Troy, NY 12180, USA
[2]Chemical and Biomolecular Engineering, University of California, Berkeley, CA 94720, USA
[3]Bioengineering, University of California, Berkeley, CA 94720, USA
[4]Department of Bioengineering and Institute for Bioengineering and Biosciences, Instituto Superior Técnico, Universidade de Lisboa, Lisbon 1049-001, Portugal
*Correspondence: dordick@rpi.edu
http://dx.doi.org/10.1016/j.stemcr.2016.10.001

SUMMARY

A 3D cell culture chip was used for high-throughput screening of a human neural progenitor cell line. The differential toxicity of 24 compounds was determined on undifferentiated and differentiating NPCs. Five compounds led to significant differences in IC_{50} values between undifferentiated and differentiating cultures. This platform has potential use in phenotypic screening to elucidate molecular toxicology on human stem cells.

INTRODUCTION

Human embryonic, induced pluripotent, and adult stem cells are invaluable tools for drug discovery, human toxicology, and studies on human development. Through controlled stem cell differentiation, large quantities of cell types from various tissues have been generated, including lung, liver, intestine, and neural to name a few (Lancaster et al., 2013; Spence et al., 2011; Takebe et al., 2013; Wetsel et al., 2011). In all cases, expansion in a stem or progenitor cell state is required to achieve large cell numbers, followed by differentiation into terminally differentiated fates. Such expansion and differentiation processes depend on myriad soluble factors, and cellular interactions with other cells and the extracellular matrix (ECM). This complexity has motivated the development of high-throughput screening tools to explore the large combinatorial signaling "space" associated with stem cell differentiation. The development of high-throughput screening platforms that emulate the complexity of natural stem cell microenvironments has provided basic insights into stem cell regulation, as well as enabling numerous applications (Lutolf et al., 2009; Soen et al., 2006). For example, toxicity screening systems that use human stem/progenitor cells and their terminally differentiated derivatives may help improve preclinical characterization of drug candidates and thereby reduce the extremely high attrition rates that plague drug development, often due to unforeseen toxicity (Hay et al., 2014; Ledford, 2011).

Cell-based microarrays have been used to screen for the effects of arrayed ECM proteins on neural stem cell proliferation and differentiation (Soen et al., 2006). The experimental platforms for these cell-based studies, however, have been almost uniformly focused on two-dimensional (2D) environments, despite the fact that three-dimensional (3D) approaches have gained increasing interest (Ranga et al., 2014). In particular, 3D culture models may better mimic the in vivo cellular microenvironment, which can be critical in phenotypic screens (Barcellos-Hoff et al., 1989; Yarmush and King, 2009). However, challenges remain in developing and implementing microarray-based 3D systems for screening purposes, including developing stable natural or synthetic matrices that enable rapid diffusion of soluble factors and reagents for 3D-based studies (Li et al., 2014; Ranga et al., 2014; Yarmush and King, 2009). Furthermore, samples arrayed on a surface often share the same culture medium, whereas it would be desirable to screen many liquid media compositions using a 3D culture platform. To address these limitations, we developed a microfabricated plastic "chip" system capable of 3D cell culture at the nanoliter scale. This platform, which has been described previously for rapid toxicity screening of compounds with human hepatocellular carcinoma cells, consists of two complementary chips that "stamp" together to generate up to 532 independent microscale cultures per chip (Kwon et al., 2014).

In the current work, we use this chip-based microarray platform to culture and perform high-content screening of a human neural progenitor cell (NPC) line, ReNcell VM, in 3D microscale cultures encapsulated within Matrigel. The NPCs were maintained in their multipotent state or induced to differentiate, and the resultant cultures were used to quantify expression of key cellular proteins, screen for acute toxicity and anti-proliferative effects of a diverse range of biologically active compounds, and investigate whether variable toxic sensitivities exist between

This is an open access article under the CC BY license (http://creativecommons.org/licenses/by/4.0/).

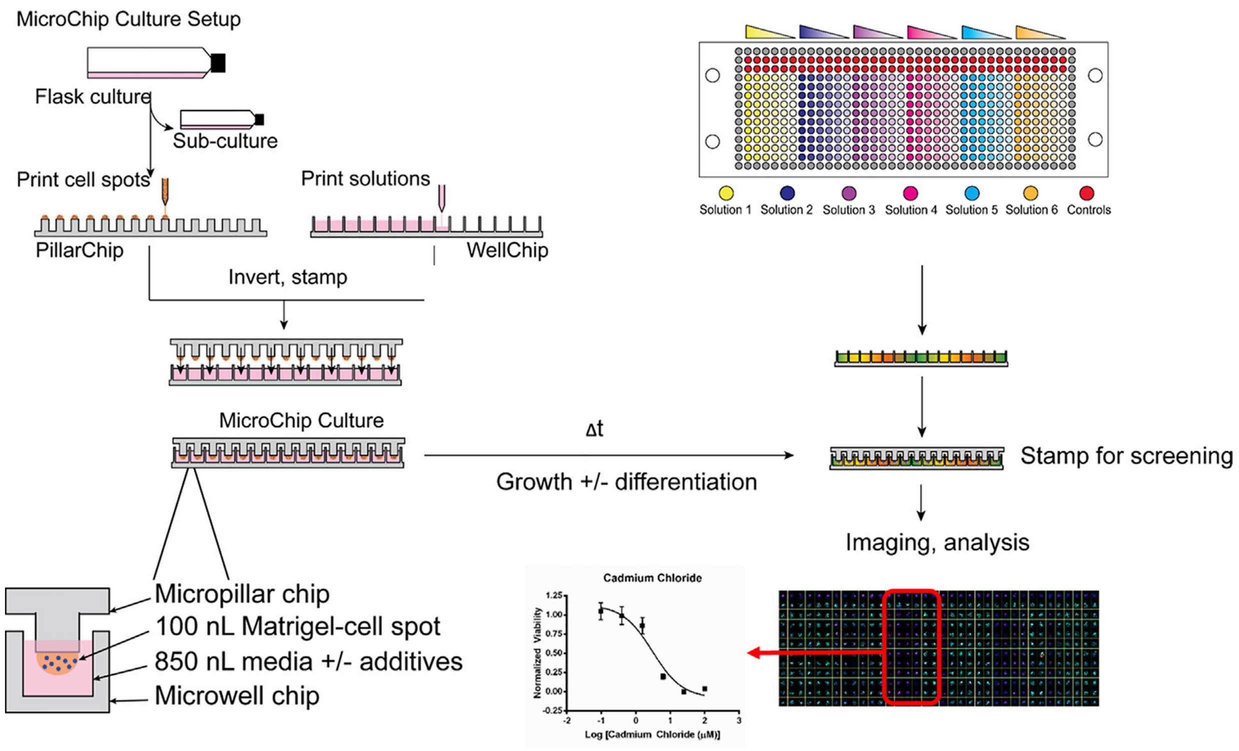

Figure 1. Microarray Chip Platform

The platform consists of two complementary polystyrene microchips measuring 25 × 75 mm, each containing 532 micropillars (PillarChip) or microwells (WellChip) arranged in a 14 × 38 array. The feature-to-feature distance of pillars and wells is 1.5 mm with diameters of 750 μm and 1.2 mm, respectively. Cell spots consist of Matrigel-encapsulated cells spotted as 100 nL cultures atop micropillars and the microwells contain 850 nL of medium. Medium change is straightforward and consists of lifting a PillarChip from one WellChip and stamping into another containing fresh medium. Fluorescence-based endpoint assays are used to measure viability, proliferation, and protein expression of on-chip cultures.

undifferentiated and differentiating cells. Such information provides new insight into how miniaturization of 3D cell culture impacts NPC proliferation and differentiation for use in high-content, high-throughput screening of toxicity and cell phenotype.

RESULTS

Characterization of 3D Microarray NPC Cultures

The microarray chip platform consists of two complementary polystyrene microchips with 532 micropillars (PillarChip) or microwells (WellChip). Cells are encapsulated in a 3D matrix atop a PillarChip, which is submerged into a WellChip for on-chip culture (Figure 1). In general, 3D cultures require cumbersome imaging and analytical techniques (e.g., confocal imaging and stack reconstruction) (Li et al., 2015). To simplify this process, on-chip 3D cultures were rapidly dried for image processing and analysis; however, for samples labeled with fluorescent live-cell stains, this resulted in cell lysis. It was reasoned this cell lysis could be prevented with a lyoprotectant, and addition of 50 mM trehalose to the Dulbecco's phosphate-buffered saline (DPBS) wash solution prior to drying indeed preserved cytoplasmic staining of calcein, enabling the simplified imaging approach (Figure S1A).

The sensitivity of fluorescent live-cell stains to assess quantitatively cell spots was determined using on-chip cultures prepared with a range of cell densities (up to 1×10^7 cells/mL). As anticipated, we observed a near-linear relationship between fluorescence intensity and seeding density for calcein and Hoechst 33342, which demonstrated that the fluorescence intensities are representative of living and total cells, respectively, within the 3D on-chip cultures (Figures 2A–2C). Single-cell analysis revealed on-chip post-printing viability of NPC cultures to be 84% ± 5% and 80% ± 6% (mean ± SD, n = 396 biological replicates) in 0.5% and 1% Matrigel, respectively, at 300 cells per spot (Figure S1B). ReNcell VM, the human NPC line used in this study, is a commercially available, *v-myc* immortalized region-specific human neural progenitor cell line derived from ventral mesencephalon bulk tissue

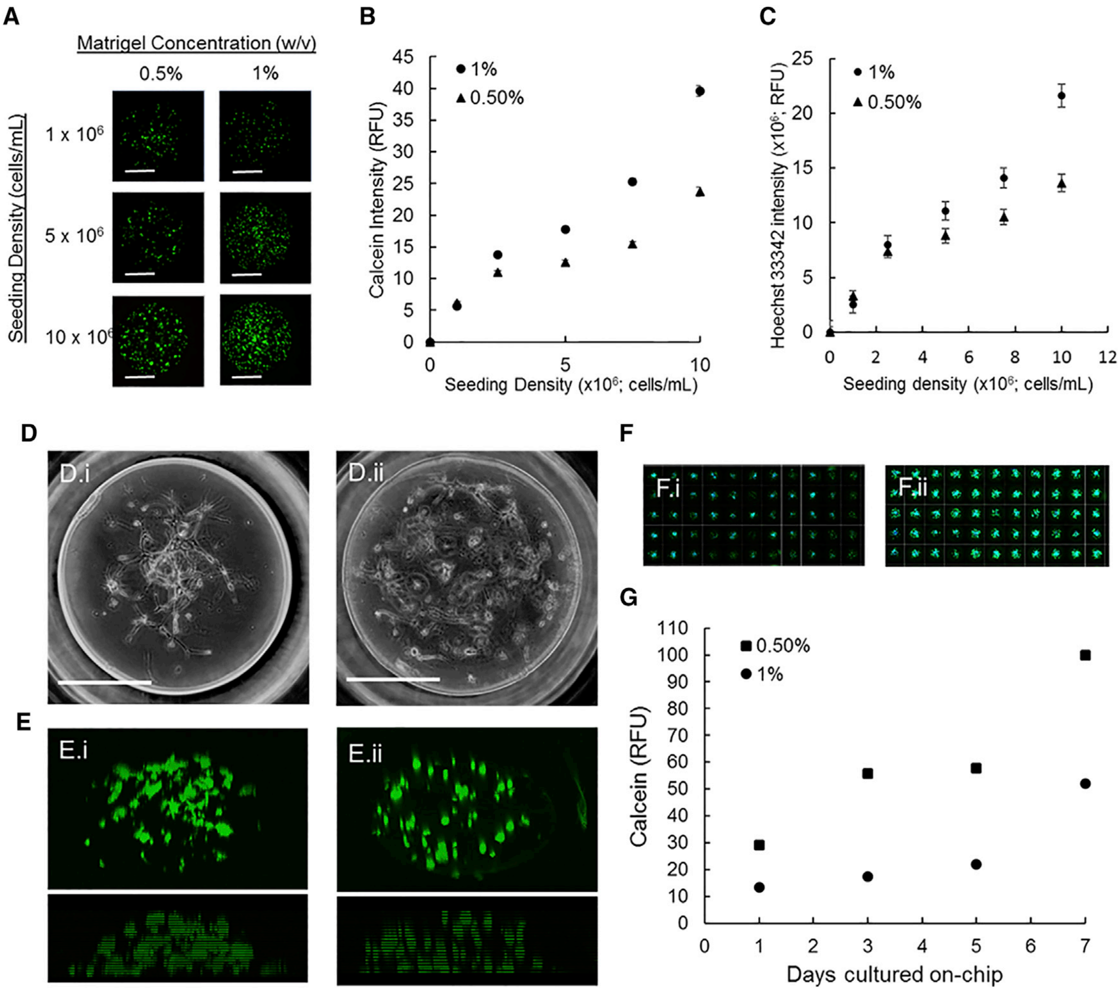

Figure 2. On-Chip Viability Assay Sensitivity and On-Chip NPC Culture Characterization

(A–C) Representative fluorescence images of assayed cell spots, seeded with up to 1 × 10^7 cells/spot in 0.5% or 1% Matrigel (A). The background adjusted mean fluorescence intensity ± SEM (n = 72 biological replicates) is plotted against seeding density in both (●) 0.5% and (▲) 1% (w/v) Matrigel for calcein (B) and Hoechst 33342 (C).

(D and E) Phase contrast (D) and z stack (E) reconstructed confocal images of NPCs cultured on-chip in 0.5% (i) and 1% (ii) Matrigel for 3 days.

(F) Growth assessed by calcein staining intensity is qualitatively apparent in fluorescent image montages (compiled with Cellomics software) when comparing staining between on-chip cultures after 1 (i) and 7 (ii) days of culture.

(G) Quantified calcein intensity of ReNcell VM NPCs cultured on-chip in (■) 0.5% and (●) 1% Matrigel over time (one time-lapse experiment, where each point represents the mean ± SEM of 396 biological replicates). Scale bars, 300 μm.

dissections of a 10-week fetus (Donato et al., 2007). Arrayed, undifferentiated NPCs encapsulated in Matrigel (0.5% and 1% [w/v]) had a compact cylindrical/spherical shape (Figure 2D), notably different from their spread and flattened appearance in 2D monolayer cultures (Figure S1C). Calcein staining indicated that the cells were uniformly distributed throughout the cell spots, and the estimated average spot height (n = 3 biological replicates) was 250 ± 17 μm and 204 ± 5 μm for 0.5% and 1% Matrigel, respectively (Figure 2E).

The effects of encapsulating and soluble Matrigel concentration, fibroblast growth factor 2 (FGF2) and epidermal growth factor (EGF) concentrations, seeding density, and frequency of medium change were screened in a 2^5 factorial design experiment, which revealed daily medium change had a significant impact on growth and viability on-chip, and was thus employed in subsequent experiments (Figure S2). The concentration of EGF and FGF2, and soluble or encapsulating Matrigel, had statistically insignificant effects on cell viability and growth. In addition, cultures

seeded at 500 cells/spot had a significantly higher calcein fluorescence than those seeded at 300 cells/spot, which demonstrated that the cultures remained viable at higher cell densities.

The effect of culture time on NPC proliferation when cultured within Matrigel on-chip was measured in a time-lapse experiment. Four on-chip cultures were prepared with either 0.5% or 1% Matrigel, and viability across an entire chip was measured after 1, 3, 5, and 7 days of culture. As anticipated, calcein fluorescence intensity per spot increased over time (Figures 2F and 2G). NPCs cultured on-chip experienced a lag phase (~1–2 days) followed by growth with calculated cell doubling times of ~67 and ~70 hr for 0.5% and 1% Matrigel, respectively. Ultimately, 1% Matrigel encapsulation resulted in increased physical stability of cell spots and was used for subsequent screening.

Protein Expression of NPCs in 3D Microscale Cultures On-Chip

Several proteins associated with the maintenance and/or function of various cell states were used as markers to characterize undifferentiated and differentiated NPC phenotypes. Undifferentiated NPCs express the intermediate filament Nestin (NES) and transcription factor SOX2 (Komitova and Eriksson, 2004; Park et al., 2010), and can express additional markers such as glial fibrillary acidic protein (GFAP), an intermediate filament also expressed in terminally differentiated astrocytes (Goldman, 2003). Differentiating NPCs begin to express proteins associated with specific terminal lineages, e.g., astrocyte differentiation can be characterized by increased expression of GFAP and S100β, a regulatory calcium-binding protein (Bignami et al., 1972; Markiewicz and Lukomska, 2006; Raponi et al., 2007). Analogously, progenitor cells differentiating into neurons transiently express doublecortin (DCX), a microtubule-associated protein, before terminal differentiation and expression of βIII tubulin (TUBB3), a microtubule protein (Couillard-Despres et al., 2006; Roskams et al., 1998). Cells differentiating into oligodendrocytes express CNPase (CNP), an enzyme involved in myelination (Sprinkle, 1989).

Withdrawal of EGF and FGF2 from culture medium is expected to induce differentiation of ReNcell VM, during which time the stem/progenitor cells experience significant changes in morphology, protein expression, and function to develop into terminally differentiated phenotypes (Donato et al., 2007; Sun et al., 2008). Immunofluorescence characterization of protein markers associated with undifferentiated and differentiated cell states before and after induction of differentiation has, to our knowledge, not been done with this cell line. Thus, we proceeded to assess differentiation induced by EGF and FGF2 withdrawal using both immunofluorescence and western blot analysis. To address antibody quality, primary antibodies were validated using human cell lines to verify specificity for immunofluorescence (Figures S3A–S3D).

ReNcell VM cultured as monolayers (2D) or embedded within 1% Matrigel (3D) were cultured with and without EGF and FGF2 to assess protein expression. For undifferentiated 2D cultures (+EGF/FGF2), expression of DCX, TUBB3, GFAP, SOX2, and NES was detected by both western analysis (Figure 3A) and immunofluorescence (Figure 3E). Differentiation induced through removal of EGF and FGF2 for 10 days resulted in drastic morphological changes (Figure S1C). Western analysis revealed that the loading control-relative expression of TUBB3, GFAP, and CNP increased while, unexpectedly, the expression of NES and SOX2 persisted (Figure 3A). A second SOX2 antibody was used in a repeat experiment to account for the possibility of a non-specific signal, yielding a similar result (Figure 3B). Notably, 2D differentiation for 6 weeks resulted in substantial SOX2 downregulation (Figure 3C). Immunofluorescence analysis revealed that differentiating ReNcell VM continued to express SOX2, NES, DCX, and GFAP while the number of cells expressing TUBB3 increased (Figure 3F) after 10 days. S100β and CNP expression was undetected in 2D cultures by immunofluorescence (data not shown). Western analysis of 3D slab cultures revealed an increase in relative expression of TUBB3 and GFAP after culture without EGF and FGF2 (Figure 3D), similar to 2D. However, there were also notable differences between monolayer and 3D slab differentiation. Specifically, in the 3D slab cultures CNP was undetected, and there was an increase in relative expression of SOX2 and NES after 10 days without EGF and FGF2.

Analysis of protein expression on-chip was performed using a modified in-cell, on-chip immunofluorescence assay. Sensitivity of the assay was first determined by spotting undifferentiated NPCs at varied cell densities (1, 2.5, and 5 × 10^6 cells/mL) in 1% Matrigel followed by immunofluorescence-based detection of NES and GFAP. As anticipated, the total fluorescence intensity of each marker increased with spotting cell density (Figure S3E). Dividing immunofluorescence intensity by Hoechst 33342 intensity within each spot resulted in similar normalized values for all seeding densities (Figure S3F), suggesting that Hoechst 33342 intensity normalization accounts for differences in cell number when analyzing protein expression on-chip. Expression of protein markers indicative of cell fate was then quantified on-chip after a 5-day culture with EGF and FGF2 and again after 10 subsequent days without EGF and FGF2 to induce differentiation. Undifferentiated cultures (+EGF/FGF2) expressed SOX2, NES, GFAP, and DCX after culture on-chip (Figure 4A). Differentiation (−EGF/FGF2) resulted in growth of cell extensions,

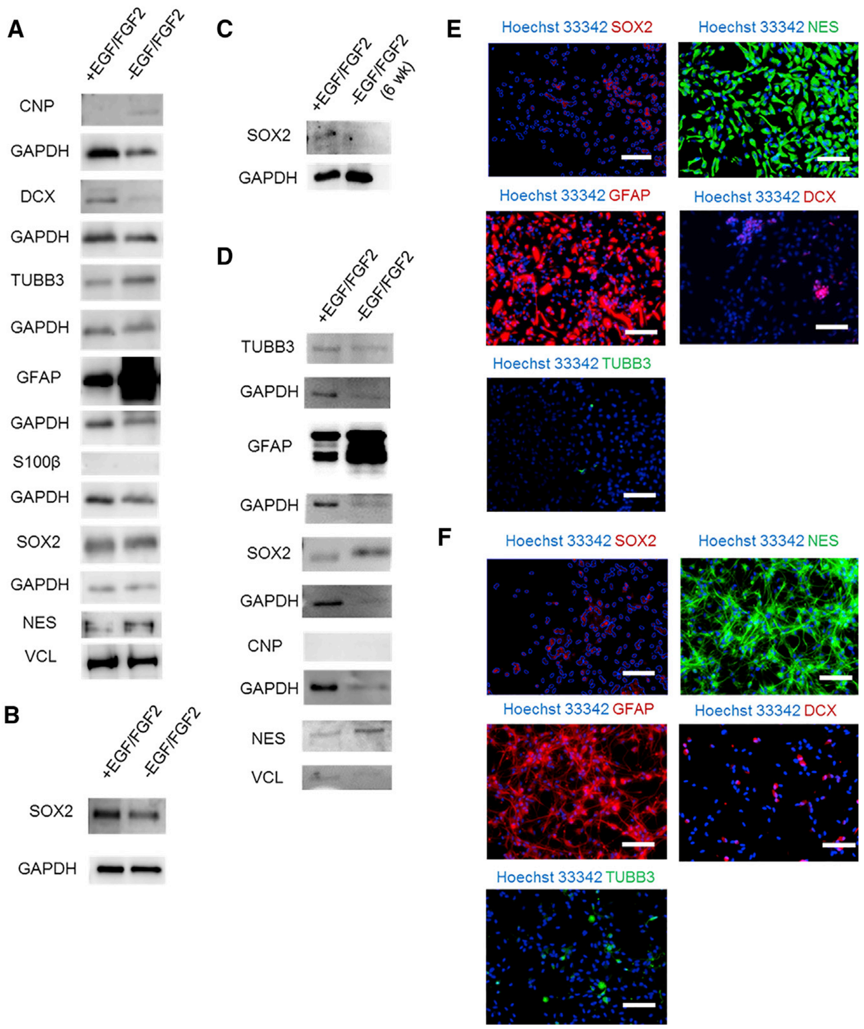

Figure 3. Off-Chip Analysis of ReNcell VM NPC Protein Expression in 2D Monolayer and 3D Matrigel Cultures

(A) Western blot analysis of fate-specific protein markers in ReNcell VM 2D monolayer cultures before and after a 10-day culture without EGF and FGF2 to induce differentiation.

(B) SOX2 western blot analysis in a 2D monolayer culture with an additional antibody specific for SOX2 (Cell Signaling Technologies).

(C) SOX2 western blot analysis in 2D monolayer culture following a 6-week differentiation.

(D–F) Western blot analysis of ReNcell VM cultured off-chip in 3D 1% Matrigel before and after growth factor withdrawal (D). Immunofluorescence analysis of undifferentiated (+EGF/FGF2) (E) and differentiating (−EGF/FGF2). (F) ReNcell VM cultured in 2D monolayer. Merged fluorescence images of the indicated protein and Hoechst 33342 (used as a counterstain) are depicted with the exception of SOX2 (nuclear staining), where the Hoechst 33342 nuclei were used to create an overlay mask. Brightness and contrast of single-channel images were adjusted with ImageJ to eliminate background using samples stained with fluorescent secondary antibody only prior to merging. GAPDH and vinculin were used as loading controls. Scale bars, 100 μm.

depicted in the immunofluorescence images of GFAP and NES (Figure 4B). Expression of SOX2 persisted while NES, TUBB3, GFAP, S100β, and DCX expression increased ($p < 0.05$, two-tailed t test; Figure 4A) in differentiating cultures.

Taken together, these results suggest that while EGF and FGF2 withdrawal is sufficient for inducing increased expression of terminal markers, the extent of differentiation is moderate after only 10 days. Nonetheless, the changes in protein expression support the conclusion the NPCs cultured without EGF and FGF2 are in the *process* of differentiating into primarily pan-astrocytic and neuronal lineages.

Acute Toxicity Screening of NPCs in 3D Microarray Cultures

To demonstrate the high-throughput screening capability of the chip platform, we evaluated the dose-response effects on cell viability of a 24-compound library with diverse structural and biological properties, consisting of approved drugs, heavy metals, and pesticides, some of which are regarded as acutely neurotoxic (Table S2). Cell proliferation was then tested for a subset of this library. For both viability and proliferation, the effect of the compounds was tested on both undifferentiated and differentiating NPC cultures, which would uncover cell-state specific (self-renewal versus differentiation) differences in toxicity.

On-chip cultures of undifferentiated and 10-day differentiated NPCs were stamped with WellChips filled with compound-containing solutions and incubated for 72 hr prior to viability assessment. Vehicle-control normalized viabilities were plotted against compound concentration to generate dose-response plots (Figures 5A and S4). The acute viability screen identified 16 compounds that substantially decreased viability (≥50% killing) relative to controls (Table S3). The dose-response data for nine of these compounds were suitable for sigmoidal curve fitting and calculation of a median inhibitory concentration (IC_{50}) value (Table 1). The sigmoidal fits for

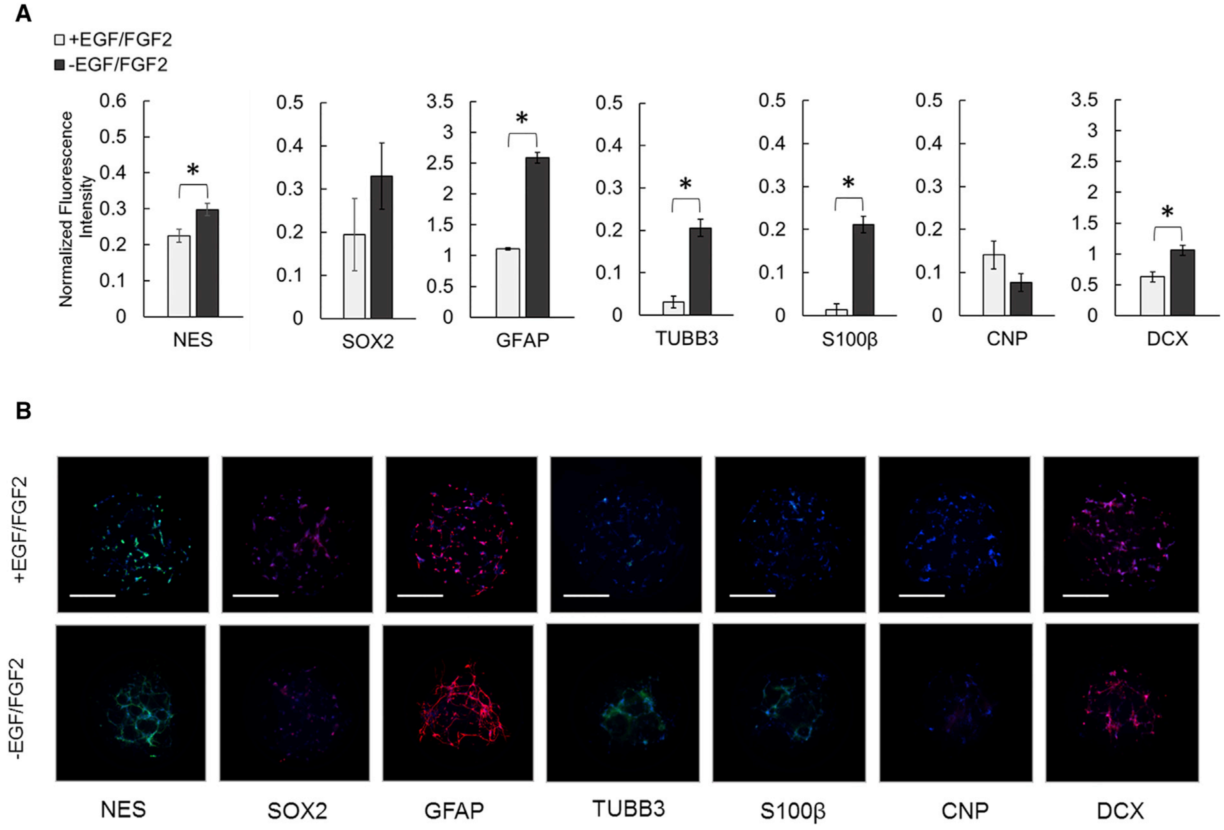

Figure 4. On-Chip Analysis of ReNcell VM NPC Protein Expression in Microscale 3D Matrigel Cultures

(A) Hoechst 33342-normalized immunofluorescence detection of cell fate-specific protein markers within on-chip NPC cultures before and after a 10-day culture without EGF and FGF2. Mean ± SEM plotted for 36 independent biological replicates per marker for each condition. *$p < 0.05$ (two-tailed Student's t test) indicates the difference between undifferentiated and differentiating cultures.

(B) Representative images of immunofluorescence stained on-chip NPC cultures before and after a 10-day culture without EGF and FGF2 (green or red); Hoechst 33342-counterstained nuclei (blue). The Cellomics analysis suite was used for single-channel brightness and contrast adjustment to eliminate background signals as determined from samples stained only with fluorescently labeled secondary antibody. Scale bars, 300 μm.

undifferentiated and differentiating NPCs were compared to assess whether differential sensitivities (i.e., different IC_{50} values) existed for the screened compounds. Statistically significant ($p < 0.05$, two-tailed t test) IC_{50} values were observed between undifferentiated and differentiating cultures for retinoic acid, doxorubicin (DOX), 5-fluorouracil (5-FU), pitavastatin, and acetaminophen. In addition, the IC_{50} values in response to cytosine arabinoside (CA) were notably different, although the statistical difference warranted further investigation ($p < 0.10$, two-tailed t test). The resulting differences in viability between undifferentiated and differentiating cultures in response to pitavastatin and 5-FU (Figures 5A and S4) was particularly interesting, with substantially greater killing of undifferentiated cultures for these compounds.

Differential toxic effects could be explained, in part, as a consequence of the different proliferative states between the undifferentiated and differentiating NPCs. To this end, the thymidine analog, 5-ethynyl-2′-deoxyuridine (EdU), was used to detect actively proliferating cells on-chip following exposure to compounds or vehicle control to determine dose-response effects on proliferation (Figures 5B and S5), and sigmoidal curves were fit, when appropriate, to obtain IC_{50} values (Table 1). We found ~60% of undifferentiated NPCs incorporated EdU over a 24-hr period as opposed to ~3% of differentiating cultures. Following exposure to 5-FU, a known antiproliferative agent, we observed a sharp decrease to no EdU incorporation in both undifferentiated and differentiating NPC cultures. In consideration with the differential cytotoxicity induced by 5-FU, this result confirms the different proliferation rates of undifferentiated and differentiating culture contributes to their differential sensitivities.

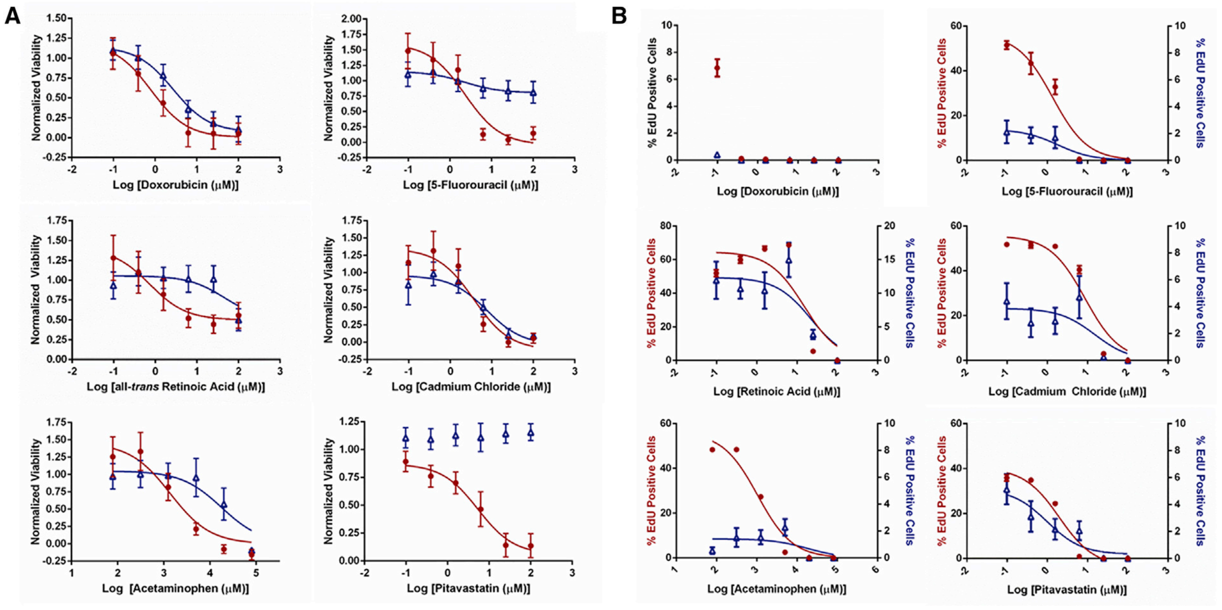

Figure 5. Dose-Response Viability and Proliferation Effects of Select Compounds on Undifferentiated and Differentiating ReNcell VM NPCs

(A) Vehicle control-normalized dose-response viability of undifferentiated (●) and differentiating (Δ) NPCs plotted against log(concentration [μM]). Mean viability ± SEM is plotted for each concentration as determined from 30 biological replicates per dose. Sigmoidal fits to the data are plotted as solid lines.

(B) Dose-response EdU incorporation of undifferentiated (●, left axis) and differentiating (Δ, right axis except DOX) NPCs plotted against log(concentration [μM]). Mean % EdU positive nuclei ± SEM is plotted for each concentration as determined from ten biological replicates. Dose-response viability and proliferation results for compounds not presented herein are available in Figures S4 and S5.

DISCUSSION

There is considerable interest in developing high-throughput technologies for microscale analysis and screening of both undifferentiated and differentiated human stem/progenitor populations to aid drug screening and toxicity analysis (Bhatia and Ingber, 2014; El-Ali et al., 2006). Animal models are often used to assess toxicity, including neurotoxicity, but they are associated with high costs and results that often have poor translation to humans (DiMasi et al., 2003; Hay et al., 2014). Human cell-based microarray screening systems are attractive because of automated systems for handling liquids that can enable screening with minimal reagent consumption. This is of particular interest in elucidating the influence of ECM factors and/or signaling pathways on stem/progenitor function and differentiation and to investigate the large combinatorial space associated with their respective microenvironments.

In the present work, we have demonstrated a chip-based microarray platform that allows for simple medium exchange on microscale 3D cultures. Importantly, on-chip growth and differentiation of a human progenitor cell line was possible for more than 2 weeks. In addition, sensitive fluorescence-based assays were compatible to screen for protein expression, cytotoxicity, and proliferation. The system presented herein is advantageous for several reasons. Miniaturization of 3D cultures, which may better emulate in vivo conditions, permits investigation under conditions where diffusional limitations are negligible (Ranga et al., 2014; Meli et al., 2014). Moreover, the modularity of a system with two complementary chips (one for cells and one for medium components) that stamp together to generate the high-throughput cell culture is potentially suitable for 3D parallel screening. This is fundamentally distinct from other high-throughput 3D microscale culture platforms, e.g., in 1536-well plates, where medium exchange is cumbersome and specialized automated systems are often optimized for 2D cultures, which restricts the duration of culture and/or screening (Ranga et al., 2014).

The growth rate of ReNcell VM cultured on-chip in 3D Matrigel was roughly three times slower than that reported for 2D cultures (Donato et al., 2007; Meli et al., 2014). The on-chip 3D cultures are prepared at a medium-to-cell ratio that is about half that typically used with larger-scale (i.e., flask) 2D cultures. Thus, the relative quantity of available nutrients as a result of scale-down could contribute to delayed growth. In agreement with this, our factorial screen

Table 1. Differential Acute Toxicity Effects on Viability and Proliferation of Undifferentiated and Differentiating ReNcell VM NPCs

	Viability Effects				Proliferation Effects			
	Undifferentiated		Differentiating		Undifferentiating		Differentiating	
Chemical	log(IC_{50} ± SEM)	IC_{50} (μM)	log(IC_{50} ± SEM)	IC_{50} (μM)	log(IC_{50} ± SEM)	IC_{50} (μM)	log(IC_{50} ± SEM)	IC_{50} (μM)
**Acetaminophen**[a]	3.1 ± 0.21	1,400	4.3 ± 0.24	18,000	3.017 ± 0.05	1,000	4.26 ± 0.52	18,200
**5-Fluorouracil**	0.36 ± 0.31	2.3	0.35 ± 0.22[b]	2.3	0.14 ± 0.10	1.35	0.26 ± 0.40	1.82
Cytosine Arabinoside[c]	−0.48 ± 0.17[b]	0.33	0.55 ± 0.55	3.5	< −1	–	< −1	–
**Retinoic Acid**[a]	−0.13 ± 0.21	0.74	1.7 ± 1.2	47	1.17 ± 0.11	15	1.35 ± 0.26	22
**Doxorubicin**[a]	−0.1 ± 0.11	0.8	0.44 ± 0.06	2.7	< −1	–	< −1	–
Cadmium Chloride	0.57 ± 0.3	3.7	0.88 ± 0.27	7.6	0.93	8.6	1.18	15
Paraquat	1.8 ± 0.18	57	1.61 ± 0.18	41	1.77 ± 0.09	59	1.48 ± 0.64	30
**Pitavistatin**	0.73 ± 0.16	5.4	–	–	0.29 ± 0.07	2.0	−0.01 ± 0.39	0.98
Diphenhydramine	2.41 ± 0.37	257	2.3 ± 0.24	189	–	–	–	–

Extracted IC_{50} values from sigmoidal fits of dose-response viability and proliferation screening displayed as log(IC_{50} ± SEM [μM]) and corresponding IC_{50} value (μM). Chemicals which elicited differential effects on undifferentiated and differentiating NPCs are highlighted in bold. Results are based on dose-response data from which each dose had 30 biological replicates unless indicated. Dose-response data not amenable to sigmoidal fitting are represented as a dash. Unlisted screened compounds were omitted because the dose-response screen did not capture the effective window, making them unfit for sigmoidal curve fitting.

[a] $p < 0.05$ (two-tailed Student's t test).

[b] Results based on data from which each dose had 20 biological replicates.

[c] $p < 0.10$ (two-tailed Student's t test) difference between IC_{50} of undifferentiated and differentiating NPCs.

identified that more frequent medium change led to higher numbers of viable cells. We reasoned it was unlikely that diffusional limitations were contributing to slower cellular growth with our ~200 μm cell spots because Ranga et al. (2014) demonstrated that soluble nutrients rapidly diffused throughout PEG-based hydrogels of ~500 μm in thickness, while Meli et al. (2012) reported that oxygen diffusional limitation in 3D microscale cultures was negligible. Nonetheless, crosslinked Matrigel has been reported to have pore sizes <2 μm, which could result in physical constraints on cell growth in the encapsulated environment (Zaman et al., 2006). Thus, the slower growth observed on-chip could be a result of the need for NPCs to remodel and/or displace the encapsulating matrix to accommodate cell growth and cell-cell interactions. Regardless, conditions that mediate slow growth can be compatible with stem cell differentiation.

Our observation that the expression of multipotency markers (SOX2 and NES) persisted in 2D cultures, and even increased relative to undifferentiated cultures in 3D after 10 days of differentiation, was of note. Nevertheless, it has been demonstrated that NES expression decreases slowly; human midbrain neural progenitors have been shown to retain NES expression beyond 4 weeks of differentiation both in vitro and in vivo (Sun et al., 2008; Zhang et al., 2001). However, SOX2 expression is expected to decrease following differentiation into an oligopotent cell state (Bani-Yaghoub et al., 2006; Graham et al., 2003). ReNcell VM was immortalized by *v-myc* gene transduction of primary fetal neural progenitors. Recently Pino-Barrio et al. (2015) reported that inducing *v-myc* expression in NPCs increases SOX2 expression (Donato et al., 2007). Therefore, it is possible that overexpression of *v-myc* impacts the downregulation of SOX2 during differentiation of ReNcell VM, which could prolong or interfere with terminal differentiation. However, the downregulation of SOX2 after a 6-week differentiation suggests that *v-myc* immortalization does not inhibit the eventual downregulation of this multipotency marker.

Removal of growth factor-mediated self-renewal signaling in NPC cultures should result in increased expression of terminal differentiation markers (Graham et al., 2003; Sun et al., 2008). Indeed, in 2D monolayer cultures, ReNcell VM increased expression of proteins associated with terminal differentiation into astrocytes, neurons, and oligodendrocytes. Other studies have reported similar results with ReNcell VM after differentiation induced by growth factor withdrawal in monolayer culture (Donato et al., 2007; Hoffrogge et al., 2006; Lange et al., 2011), and terminal neuronal differentiation has been limited without employing methods aimed at directing neuronal differentiation. For example, Lange et al. (2011) reported that <3% of cells

expressed TUBB3 after 7 days of differentiation induced by growth factor withdrawal. In 3D Matrigel, observed changes in protein marker expression upon differentiation were notably different compared with 2D cultures, which suggests that Matrigel impacts the spontaneous differentiation of ReNcell VM. Matrigel is a complex mixture of diverse ECM components, including proteoglycans, which may also impact differentiation. In addition, self-renewal growth factors (EGF and/or FGF2) may remain in the 3D matrix even after their removal from medium due to cytokine-matrix interactions, which could influence the progression of NPC differentiation. Indeed, Akashi et al. (2005) observed reduced diffusion of FGF2 through Matrigel matrices, suggesting that its elimination from the microenvironment could be delayed.

Moderate increased expression of terminal markers with retained expression of multipotency markers after 10 days suggests that spontaneous terminal ReNcell VM differentiation progresses slowly, and this may not be unique. Shi et al. (2012) reported that terminal differentiation of iPSC-derived forebrain neural progenitors entailed a 20-day culture. Furthermore, Choi et al. (2014) reported differentiating ReNcell VM for 6 weeks or longer to derive high proportions of terminally differentiated cells in 3D Matrigel. Thus, long-term differentiation may be required to obtain a high degree of terminally differentiated neural cells with ReNcell VM. Nonetheless, while not substantially terminally differentiated, ReNcell VM cultured without growth factors for 10 days were distinct from those grown in the presence of EGF and FGF2 and are likely a mixture of NPCs at various stages of differentiation.

Of the 16 compounds that substantially decreased viability, seven had IC_{50} concentrations <100 μM. This included acutely toxic compounds (e.g., paraquat, $CdCl_2$) with IC_{50} values consistent with literature values (Breier et al., 2008). Compounds without potent neurotoxicity (e.g., acetaminophen, caffeine, diphenhydramine) were found to have no cytotoxicity at functionally relevant concentrations, and only at concentrations associated with systemic human toxicity did decreases in viability occur (Brent et al., 2011; Clemedson et al., 2007; Radovanovic et al., 2000). $PbCl_2$ and $MnCl_2$ did not reduce cell viability for either undifferentiated or differentiating cultures. Their neurotoxicity is associated with complex mechanisms that often do not manifest as acute cytotoxicity (Neal and Guilarte, 2013). Taken together, these results begin to define the utility of the immortalized ReNcell VM for assessing chemical neurotoxicity.

Five compounds elicited differential effects on undifferentiated and differentiating NPCs. All-*trans* retinoic acid is an important endogenous neurodevelopmental signaling small molecule that can also act as a developmental teratogen when dysregulated (Lammer et al., 1985; McCaffery et al., 2003), and undifferentiated NPCs exhibited a lower IC_{50} in response to retinoic acid versus differentiating NPCs. In addition, retinoic acid increased the average number of proliferating cells within differentiated cultures by greater than 3-fold at lower concentrations. Notably, the neurodevelopmental effects and sensitivity of rats to retinoic acid has previously been found to be dependent on the concentration and onset of exposure (McCaffery et al., 2003). As highlighted above, the acetaminophen concentrations needed to observe toxicity were above those that would be pharmacologically effective, although a large difference in sensitivity was observed between undifferentiated and differentiating cultures.

Pitavastatin, a 3-hydroxy-3-methyl-glutaryl-coenzyme A (HmG-CoA) reductase inhibitor, was cytotoxic only to undifferentiated NPCs, whereas another HmG-CoA reductase inhibitor, cilastatin, had no effect. Similarly, 5-FU was substantially more toxic to undifferentiated cultures and resulted in nearly complete killing while minimally impacting differentiating cultures. Both drugs were also found to inhibit the proliferation of NPC cultures, which suggests that the mechanism of toxicity was predominantly anti-proliferative (in agreement with their known mechanisms of toxicity) because they did not decrease the viability of slowly proliferating differentiating NPCs (Chan et al., 2003; Diasio and Harris, 1989). The observation that pitavastatin inhibited proliferation and that cilastatin did not is consistent with the finding of Chan et al. (2003) that statins have highly variable anti-proliferative effects on various cell lines and cancers.

DOX and CA are well-known chemotherapeutics with anti-proliferative and cytotoxic activity (Gewirtz, 1999; Momparler, 1982). For undifferentiated NPCs, DOX gave lower NPC viability (lower IC_{50}), and to a lesser extent so did CA ($p < 0.10$), in comparison with differentiating NPCs. Because ~3% of differentiating NPCs actively proliferated, the effects of DOX and CA suggest that cytotoxicity was the predominant mechanism of killing differentiating NPCs. This result also suggests that undifferentiated NPCs may be more sensitive to these compounds as a result of both anti-proliferative and cytotoxic mechanisms being effective against the undifferentiated, highly proliferative cell state.

The results presented herein demonstrate the application of a microarray chip platform as a sensitive screening tool capable of detecting differential toxicities of biologically active compounds in human NPCs of varied levels of differentiation. Given the need to develop in vitro screening platforms that can assess the activity and toxicity of chemicals on human cells, the chip platform may serve as a 3D phenotypic screening tool to better predict toxicity of, or identify, bioactive compounds in human stem/progenitor cells. Further research on non-immortalized NPCs may

provide useful insights into the influence of specific compounds on stem cells and their progeny of various fates.

EXPERIMENTAL PROCEDURES

Human Neural Progenitor Cell Culture

ReNcell VM (Millipore) was used in this study at passage number 16 or lower. Cells were grown on laminin (Sigma)-coated T25 or T75 tissue-culture-treated flasks in complete growth medium composed of ReNcell Maintenance Medium (Millipore) containing 20 ng/mL FGF2 (Millipore), 20 ng/mL EGF (Millipore), and 1% (v/v) penicillin-streptomycin (Gibco). Cells were passaged using Accutase and re-plated at 10,000 cells/cm^2 when 90% confluent. Medium was changed the day after passaging and every second day after that. For off-chip 3D culture, ReNcell VM were seeded at 890,000 cells/mL in 100 μL of 1% Matrigel in 8-well chamber microscope slides (Nunc) with daily medium change. All cultures were maintained at 37°C and 5% CO_2.

3D Microarray Culture Preparation

Polystyrene micropillar and microwell chips (SEMCO) were exposed to UV (302 nm) light (9 cm from source) for 4 hr using a 96-W transilluminator (Syngene GVM-30), following a similar procedure to that of Kohen et al. (2009), which helped maintain the cell spots atop the micropillars without covalent attachment. An enclosed MicroSys 5100-4SQ (DigiLab) non-contact robotic microarray spotting system was used for printing. During operation, the relative humidity was maintained above 95% to reduce evaporation. The MicroSys 5100-4SQ spotting head was modified with two water-block assemblies for circulation of chilled ethylene glycol-water to maintain the printer head between 2 and 8°C during Matrigel spotting.

To prepare microarray cultures, 850 nL of complete growth medium was printed into WellChips and covered with gas-permeable sealing membranes (Diversified Biotech) and stored in an incubator (37°C and 5% CO_2). High-concentration growth factor-reduced Matrigel (Corning Life Sciences) was used for 1% (w/v) encapsulation and growth factor-reduced phenol red-free Matrigel (Corning Life Sciences) was used for 0.5% (w/v) encapsulation. Cooled suspensions of newly passaged ReNcell VM were mixed with Matrigel in a ratio such that the final cell concentration was 3×10^6 cells/mL, unless otherwise noted, with final Matrigel concentrations of either 0.5% or 1% (w/v) and stored on ice until printing. The cooled microarray system was used to print the Matrigel-cell mixtures onto PillarChips (~300 cells within each spot). Following printing, the PillarChips were incubated 4 min at 25°C followed by 10 min at 37°C in a humid chamber to polymerize the Matrigel. After gelation, a PillarChip was stamped into a WellChip containing warmed complete growth medium and stored in a humidified chamber for culture. The medium was changed daily unless otherwise noted. Periphery wells were not analyzed to prevent potential evaporation from affecting the results.

Live/Dead Viability Assay

Cell viability was determined in 3D on-chip cultures using a mammalian cell viability assay (LIVE/DEAD, Invitrogen). Prior to the assay, dead cell controls were prepared with 30 min incubation in a WellChip containing either complete medium or 0.5% (w/v) saponin-DMEM/F12 medium in its microwells. PillarChips were rinsed twice with warm DPBS and incubated for 25 min in DPBS with 1 g/L glucose, 4 μM calcein-AM, 2 μM ethidium homodimer-I, and 5 mg/mL Hoechst 33342 at 5% CO_2 and 37°C. After staining, PillarChips were rinsed twice with warm DPBS and incubated in DPBS containing 50 mM trehalose (Sigma) for 10 min before rapid drying with nitrogen and storage in the dark. Dried PillarChips were imaged with a Thermo Scientific Cellomics ArrayScan VTI high-content system.

In-Cell Immunofluorescence Assays

Undifferentiated NPCs were plated on laminin-coated 96-well plates and cultured for 3 days in complete growth medium prior to fixation. Differentiated NPCs were prepared by expanding undifferentiated NPCs for 3 days, followed by differentiation in medium without EGF and FGF2 for 10 days with daily medium change to emulate on-chip conditions. Cells were rinsed with warm DPBS and fixed with 4% (w/v) formaldehyde and 0.25% (w/v) glutaraldehyde in DPBS for 20 min at room temperature. Fixed cells were permeated with 0.25% (v/v) Triton X-100 in DPBS for 10 min, rinsed with DPBS, and quenched for 30 min in DPBS with 2 mg/mL sodium borohydride (Sigma). Cells were blocked overnight at 4°C in DPBS with 5% (w/v) BSA and 1% (v/v) goat serum and incubated in primary antibody solutions diluted into DPBS containing 1% (w/v) BSA and 10 ppm antifoam C (Sigma) overnight at 4°C. Samples were washed three times with DPBS for 2 hr before repeating the antibody incubation step for secondary antibodies. A complete list of primary and secondary antibodies, vendors, and dilutions used is presented in Table S1. After incubation with secondary antibodies, samples were washed three times with DPBS for 2 hr before 10 min incubation in DPBS with 5 μg/mL Hoechst 33342 and a final rinse with DPBS. An inverted fluorescent microscope was used for imaging and ImageJ was used to process images.

On-chip undifferentiated NPCs were analyzed after 5 days and differentiated cells after a 10-day differentiation following a 5-day expansion. Antibody incubations were performed in 750 nL of antibody containing solutions on-chip. After the final washes, PillarChips were rinsed with DPBS containing 50 mM trehalose for 10 min before being completely dried with nitrogen and stored in the dark. Dried PillarChips were imaged using a Thermo Scientific Cellomics ArrayScan VTI high-content system. Control cell spots incubated solely with fluorescently labeled secondary antibodies yielded low, non-specific background signals that were subtracted during analysis.

Western Blotting

Protein concentration of lysates was estimated using a BCA assay (Sigma). Equal amounts of protein (~10 μg) were loaded into either 8% or 12% cast SDS-PAGE gels. Gels were resolved at 120 V in a Tris-glycine buffer and electrotransferred to a nitrocellulose membrane for either 60 min at 100 V (12% gel) or overnight at 30 V (8% gel) at 4°C. Membranes were rinsed with Tris-buffered saline (TBS), blocked in 5% (w/v) BSA TBS for 1 hr at room temperature, and incubated overnight at 4°C in primary antibody solutions diluted

in blocking buffer with 0.05% (v/v) Tween 20. Membranes were rinsed with TBS containing 0.05% Tween 20 (TBST) and incubated with an appropriate secondary antibody diluted in TBS with 3% BSA for 90 min at room temperature. Membranes were washed with TBST and then TBS before incubation with chemiluminescence solution (SuperSignal Pico, Pierce) for 10 min. Membranes were imaged with a Bio-Rad ChemiDoc and subsequently stripped using a stripping buffer (200 mM glycine, 1% [v/v] Tween 20, 0.1% [w/v] SDS [pH 2]) and evaluated for efficient stripping with chemiluminescence before being reused with the above procedure for detection of loading control proteins (GAPDH for 12% gels, vinculin for 8% gels).

On-Chip Cytotoxicity Assay

Undifferentiated and differentiated microchip cultures were prepared as described above. Chemicals were purchased from Sigma or the NIH Small Molecule Repository (Table S2). Concentrated stocks were prepared and diluted into ReNcell Maintenance Medium to prepare the highest screened concentration solution for each compound. Four-fold serial dilutions were made in ReNcell Maintenance Medium, with all solutions having a final concentration of 0.5% or 1% DMSO (v/v) and 20 μg/mL Matrigel. For undifferentiated cultures, the solutions also contained 20 ng/mL of EGF and FGF2. These solutions were printed into WellChips, covered with a gas-permeable membrane, and warmed at 37°C for at least 5 min before stamping with cells. Each WellChip contained six compounds each at six concentrations (n = 10 biological replicates for each concentration) and the appropriate medium containing 0.5% or 1% DMSO for vehicle and dead controls (n = 32 biological replicates per control). PillarChip cultures were incubated with compounds for 72 hr with daily medium exchange to freshly prepared WellChips containing compounds at the appropriate concentrations. After 72 hr, PillarChips were assayed with the viability assay and when appropriate data were fit to a sigmoidal dose-response model using GraphPad Prism for IC_{50} calculation (see Supplemental Information). On-chip toxicity screens were repeated three times for undifferentiated and differentiated cultures.

On-Chip Proliferation Assay

Undifferentiated and differentiating cultures were screened for toxicity as described previously, except that after the first 48 hr of exposure 10 μM EdU (Click-iT EdU, Invitrogen) was added to the compound-containing medium for the final 24 hr. After 72 hr, on-chip cultures were fixed and processed as described previously for on-chip immunofluorescence; however, instead of antibody staining the cultures were incubated with solution containing Alexa Fluor 488-azide for 30 min as specified in the product manual. Cultures were then rinsed with DPBS for 30 min, incubated in DPBS with Hoechst 33342 (5 μg/mL) for 30 min, and washed in DPBS for 30 min before proceeding with a trehalose wash and drying as described previously. Nuclei were selected using Cellomics software from blue channel images to create a mask, which was used for identification of fluorescently labeled EdU in the green channel for quantification of actively proliferating cells over the 24-hr EdU exposure. Data were fit to a sigmoidal dose-response model using GraphPad Prism to calculate IC_{50} values.

SUPPLEMENTAL INFORMATION

Supplemental Information includes Supplemental Experimental Procedures, five figures, and three tables and can be found with this article online at http://dx.doi.org/10.1016/j.stemcr.2016.10.001.

AUTHOR CONTRIBUTIONS

J.S.D. and D.V.S. conceived the project. J.S.D., D.S.C., and D.V.S. supervised the studies. G.J.N., S.K.M., B.C.P., and J.F.P. designed experiments. G.J.N. performed experiments and analysed data. G.J.N. and J.S.D. wrote the manuscript. All authors critically revised the manuscript.

ACKNOWLEDGMENTS

The authors are grateful for the technical assistance of Drs Sergey Pryschep and Brigitte Arduini. This work was supported by NIH (ES020903) and NYSTEM (C026717).

Received: May 9, 2016
Revised: September 30, 2016
Accepted: October 3, 2016
Published: October 27, 2016

REFERENCES

Akashi, T., Minami, J., Ishige, Y., Eishi, Y., Takizawa, T., Koike, M., and Yanagishita, M. (2005). Basement membrane matrix modifies cytokine interactions between lung cancer cells and fibroblasts. Pathobiology *72*, 250–259.

Bani-Yaghoub, M., Tremblay, R.G., Lei, J.X., Zhang, D., Zurakowski, B., Sandhu, J.K., Smith, B., Ribecco-Lutkiewicz, M., Kennedy, J., Walker, P.R., et al. (2006). Role of Sox2 in the development of the mouse neocortex. Dev. Biol. *295*, 52–66.

Barcellos-Hoff, M.H., Aggeler, J., Ram, T.G., and Bissell, M.J. (1989). Functional differentiation and alveolar morphogenesis of primary mammary cultures on reconstituted basement membrane. Development *105*, 223–235.

Bhatia, S.N., and Ingber, D.E. (2014). Microfluidic organs-on-chips. Nat. Biotechnol. *32*, 760–772.

Bignami, A., Eng, L.F., Dahl, D., and Uyeda, C.T. (1972). Localization of the glial fibrillary acidic protein in astrocytes by immunofluorescence. Brain Res. *43*, 429–435.

Breier, J.M., Radio, N.M., Mundy, W.R., and Shafer, T.J. (2008). Development of a high-throughput screening assay for chemical effects on proliferation and viability of immortalized human neural progenitor cells. Toxicol. Sci. *105*, 119–133.

Brent, R.L., Christian, M.S., and Diener, R.M. (2011). Evaluation of the reproductive and developmental risks of caffeine. Birth Defects Res. B Dev. Reprod. Toxicol. *92*, 152–187.

Chan, K.K.W., Oza, A.M., and Siu, L.L. (2003). The statins as anticancer agents. Clin. Cancer Res. *9*, 10–19.

Choi, S.H., Kim, Y.H., Hebisch, M., Sliwinski, C., Lee, S., D'Avanzo, C., Chen, H., Hooli, B., Asselin, C., Muffat, J., et al. (2014). A three-dimensional human neural cell culture model of Alzheimer's disease. Nature *515*, 274–278.

Clemedson, C., Kolman, A., and Forsby, A. (2007). The integrated acute systemic toxicity project (ACuteTox) for the optimisation and validation of alternative in vitro tests. Altern. Lab. Anim. *35*, 33–38.

Couillard-Despres, S., Winner, B., Karl, C., Lindemann, G., Schmid, P., Aigner, R., Laemke, J., Bogdahn, U., Winkler, J., Bischofberger, J., et al. (2006). Targeted transgene expression in neuronal precursors: watching young neurons in the old brain. Eur. J. Neurosci. *24*, 1535–1545.

Diasio, R.B., and Harris, B.E. (1989). Clinical pharmacology of 5-fluorouracil. Clin. Pharmacokinet. *16*, 215–237.

DiMasi, J.A., Hansen, R.W., and Grabowski, H.G. (2003). The price of innovation: new estimates of drug development costs. J. Health Econ. *22*, 151–185.

Donato, R., Miljan, E.A., Hines, S.J., Aouabdi, S., Pollock, K., Patel, S., Edwards, F.A., and Sinden, J.D. (2007). Differential development of neuronal physiological responsiveness in two human neural stem cell lines. BMC Neurosci. *8*, 36.

El-Ali, J., Sorger, P.K., and Jensen, K.F. (2006). Cells on chips. Nature *442*, 403–411.

Gewirtz, D.A. (1999). A critical evaluation of the mechanisms of action proposed for the antitumor effects of the anthracycline antibiotics adriamycin and daunorubicin. Biochem. Pharmacol. *57*, 727–741.

Goldman, S. (2003). Glia as neural progenitor cells. Trends Neurosci. *26*, 590–596.

Graham, V., Khudyakov, J., Ellis, P., and Pevny, L. (2003). SOX2 functions to maintain neural progenitor identity. Neuron *39*, 749–765.

Hay, M., Thomas, D.W., Craighead, J.L., Economides, C., and Rosenthal, J. (2014). Clinical development success rates for investigational drugs. Nat. Biotechnol. *32*, 40–51.

Hoffrogge, R., Mikkat, S., Scharf, C., Beyer, S., Christoph, H., Pahnke, J., Mix, E., Berth, M., Uhrmacher, A., Zubrzycki, I.Z., et al. (2006). 2-DE proteome analysis of a proliferating and differentiating human neuronal stem cell line (ReNcell VM). Proteomics *6*, 1833–1847.

Kohen, N.T., Little, L.E., and Healy, K.E. (2009). Characterization of Matrigel interfaces during defined human embryonic stem cell culture. Biointerphases *4*, 69–79.

Komitova, M., and Eriksson, P.S. (2004). Sox-2 is expressed by neural progenitors and astroglia in the adult rat brain. Neurosci. Lett. *369*, 24–27.

Kwon, S.J., Lee, D.W., Shah, D.A., Ku, B., Jeon, S.Y., Solanki, K., Ryan, J.D., Clark, D.S., Dordick, J.S., and Lee, M.-Y. (2014). High-throughput and combinatorial gene expression on a chip for metabolism-induced toxicology screening. Nat. Commun. *5*, 3739.

Lammer, E.J., Chen, D.T., Hoar, R.M., Agnish, N.D., Benke, P.J., Braun, J.T., Curry, C.J., Fernhoff, P.M., Grix, A.W., and Lott, I.T. (1985). Retinoic acid embryopathy. N. Engl. J. Med. *313*, 837–841.

Lancaster, M.A., Renner, M., Martin, C., Wenzel, D., Bicknell, L.S., Hurles, M.E., Homfray, T., Penninger, J.M., Jackson, A.P., Knoblich, J.A., et al. (2013). Cerebral organoids model human brain development and microcephaly. Nature *501*, 373–379.

Lange, C., Mix, E., Frahm, J., Glass, Ä., Müller, J., Schmitt, O., Schmöle, A.C., Klemm, K., Ortinau, S., Hübner, R., et al. (2011). Small molecule GSK-3 inhibitors increase neurogenesis of human neural progenitor cells. Neurosci. Lett. *488*, 36–40.

Ledford, H. (2011). Ways to fix the clinical trial. Nature *477*, 7–9.

Li, X., Zhang, X., Zhao, S., Wang, J., Liu, G., and Du, Y. (2014). Micro-scaffold array chip for upgrading cell-based high-throughput drug testing to 3D using benchtop equipment. Lab Chip *14*, 471–481.

Li, L., Zhou, Q., Voss, T.C., Quick, K.L., and LaBarbera, D.V. (2015). High-throughput imaging: focusing in on drug discovery in 3D. Methods *96*, 97–102.

Lutolf, M.P., Gilbert, P.M., and Blau, H.M. (2009). Designing materials to direct stem-cell fate. Nature *462*, 433–441.

Markiewicz, I., and Lukomska, B. (2006). The role of astrocytes in the physiology and pathology of the central nervous system. Acta Neurobiol. Exp. (Wars) *66*, 343–358.

McCaffery, P.J., Adams, J., Maden, M., and Rosa-Molinar, E. (2003). Too much of a good thing: retinoic acid as an endogenous regulator of neural differentiation and exogenous teratogen. Eur. J. Neurosci. *18*, 457–472.

Meli, L., Jordan, E.T., Clark, D.S., Linhardt, R.J., and Dordick, J.S. (2012). Influence of a three-dimensional, microarray environment on human Cell culture in drug screening systems. Biomaterials *33*, 9087–9096.

Meli, L., Barbosa, H.S.C., Hickey, A.M., Gasimli, L., Nierode, G., Diogo, M.M., Linhardt, R.J., Cabral, J.M.S., and Dordick, J.S. (2014). Three dimensional cellular microarray platform for human neural stem cell differentiation and toxicology. Stem Cell Res. *13*, 36–47.

Momparler, R.L. (1982). Biochemical pharmacology of cytosine arabinoside. Med. Pediatr. Oncol. *10* (*Suppl 1*), 45–48.

Neal, A.P., and Guilarte, T.R. (2013). Mechanisms of lead and manganese neurotoxicity. Toxicol. Res. (Camb.) *2*, 99–114.

Park, D., Xiang, A.P., Mao, F.F., Zhang, L., Di, C.G., Liu, X.M., Shao, Y., Ma, B.F., Lee, J.H., Ha, K.S., et al. (2010). Nestin is required for the proper self-renewal of neural stem cells. Stem Cells *28*, 2162–2171.

Pino-Barrio, M.J., García-García, E., Menéndez, P., and Martínez-Serrano, A. (2015). V-Myc immortalizes human neural stem cells in the absence of pluripotency- associated traits. PLoS One *10*, e0118499.

Radovanovic, D., Meier, P.J., Guirguis, M., Lorent, J.P., and Kupferschmidt, H. (2000). Dose-dependent toxicity of diphenhydramine overdose. Hum. Exp. Toxicol. *19*, 489–495.

Ranga, A., Gobaa, S., Okawa, Y., Mosiewicz, K., Negro, A., and Lutolf, M.P. (2014). 3D niche microarrays for systems-level analyses of cell fate. Nat. Commun. *5*, 4324.

Raponi, E., Agenes, F., Delphin, C., Assard, N., Baudier, J., Legraverend, C., and Deloulme, J.C. (2007). S100B expression defines a state in which GFAP-expressing cells lose their neural stem cell potential and acquire a more mature developmental stage. Glia *55*, 165–177.

Roskams, A.J.I., Cai, X., and Ronnett, G.V. (1998). Expression of neuron-specific beta-III tubulin during olfactory neurogenesis in the embryonic and adult rat. Neuroscience *83*, 191–200.

Shi, Y., Kirwan, P., and Livesey, F.J. (2012). Directed differentiation of human pluripotent stem cells to cerebral cortex neurons and neural networks. Nat. Protoc. *7*, 1836–1846.

Soen, Y., Soen, Y., Mori, A., Mori, A., Palmer, T.D., Palmer, T.D., Brown, P.O., and Brown, P.O. (2006). Exploring the regulation of human neural precursor cell differentiation using arrays of signaling microenvironments. Mol. Syst. Biol. *2*, 37.

Spence, J.R., Mayhew, C.N., Rankin, S.A., Kuhar, M.F., Vallance, J.E., Tolle, K., Hoskins, E.E., Kalinichenko, V.V., Wells, S.I., Zorn, A.M., et al. (2011). Directed differentiation of human pluripotent stem cells into intestinal tissue in vitro. Nature *470*, 105–109.

Sprinkle, T.J. (1989). 2′,3′-Cyclic nucleotide 3′-phosphodiesterase, an oligodendrocyte-Schwann cell and myelin-associated enzyme of the nervous system. Crit. Rev. Neurobiol. *4*, 235–301.

Sun, Y., Pollard, S., Conti, L., Toselli, M., Biella, G., Parkin, G., Willatt, L., Falk, A., Cattaneo, E., and Smith, A. (2008). Long-term tripotent differentiation capacity of human neural stem (NS) cells in adherent culture. Mol. Cell. Neurosci. *38*, 245–258.

Takebe, T., Sekine, K., Enomura, M., Koike, H., Kimura, M., Ogaeri, T., Zhang, R.-R., Ueno, Y., Zheng, Y.-W., Koike, N., et al. (2013). Vascularized and functional human liver from an iPSC-derived organ bud transplant. Nature *499*, 481–484.

Wetsel, R.A., Wang, D., and Calame, D.G. (2011). Therapeutic potential of lung epithelial progenitor cells derived from embryonic and induced pluripotent stem cells. Annu. Rev. Med. *62*, 95–105.

Yarmush, M.L., and King, K.R. (2009). Living-cell microarrays. Annu. Rev. Biomed. Eng. *11*, 235–257.

Zaman, M.H., Trapani, L.M., Sieminski, A.L., Mackellar, D., Gong, H., Kamm, R.D., Wells, A., Lauffenburger, D.A., and Matsudaira, P. (2006). Migration of tumor cells in 3D matrices is governed by matrix stiffness along with cell-matrix adhesion and proteolysis. Proc. Natl. Acad. Sci. USA *103*, 10889–10894.

Zhang, S.C., Wernig, M., Duncan, I.D., Brüstle, O., and Thomson, J.A. (2001). In vitro differentiation of transplantable neural precursors from human embryonic stem cells. Nat. Biotechnol. *19*, 1129–1133.

Stem Cell Reports, Volume 7

Supplemental Information

High-Throughput Toxicity and Phenotypic Screening of 3D Human Neural Progenitor Cell Cultures on a Microarray Chip Platform

Gregory J. Nierode, Brian C. Perea, Sean K. McFarland, Jorge F. Pascoal, Douglas S. Clark, David V. Schaffer, and Jonathan S. Dordick

Supplemental Information

for

High-Throughput Toxicity and Phenotypic Screening of 3D Human Neural Progenitor Cell Cultures on a Microarray Chip Platform

Supplemental Figures and Tables

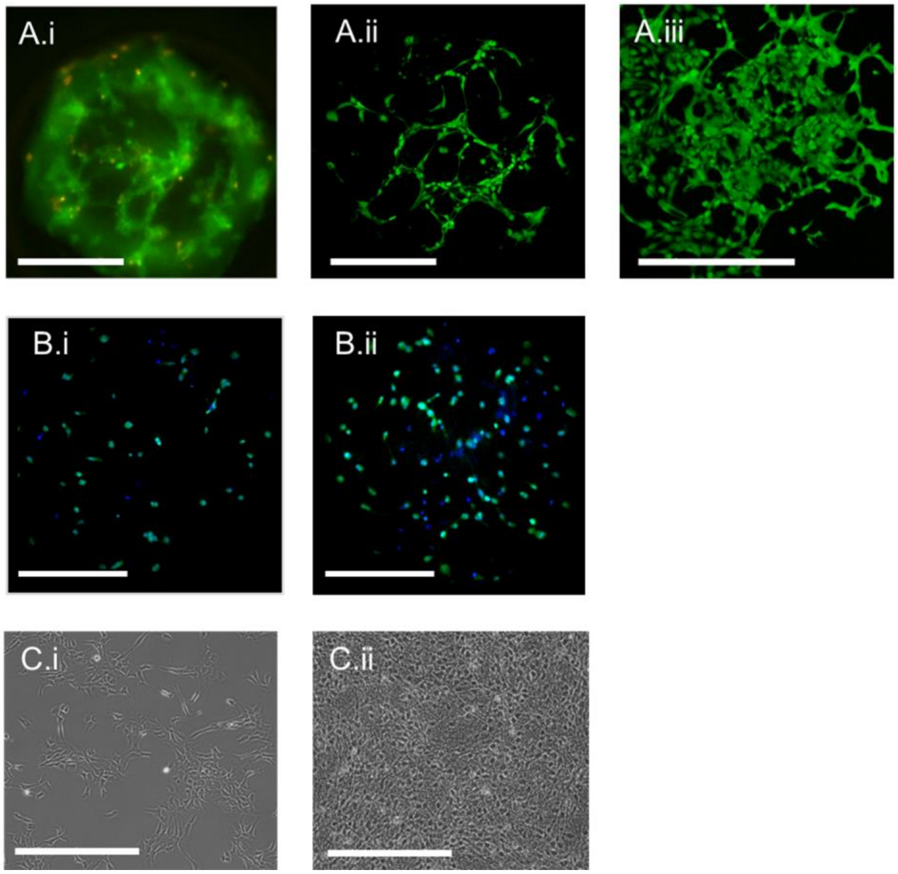

Figure S1 (related to Figure 2). ReNcell VM morphology and preservation of cytoplasmic calcein in dried 3D microarray spots. (a) Compiled fluorescent images (488 and 549 filters) of a NPC-Matrigel 3D cell spot stained with calcein and ethidium homodimer (i) when dried without trehalose, where cells lysed during dessication and entrapped calcein diffused throughout the individual cell spot during the drying process, (ii) when dried with trehalose, where cells retain cytoplasmic staining of calcein, and (iii) when imaged immediately following staining while kept in DPBS, which demonstrates the localization of calcein stain in a living, hydrated sample. (b) Post-printing viability after preparation of on-chip ReNcell VM NPC microscale cultures. After 1 day of culture, chips were stained with calcein and Hoechst 33342 stains and imaged and analyzed using Cellomics software to determine the post-printing viability in (i) 0.5% and (ii) 1% (w/v) Matrigel. Nuclei were detected and used to generate a mask of selected objects, and each object was assessed for detection of calcein staining to identify living cells. Viability was determined to be 84 ± 5% and 80 ± 6% (mean ± SD, n = 396 biological replicates) in 0.5% and 1% Matrigel, respectively. (c) Phase contrast images depicting ReNcell VM when cultured (i) with and (ii) without EGF and FGF2 in 2D monolayer cultures. Scale bars = 300 µm.

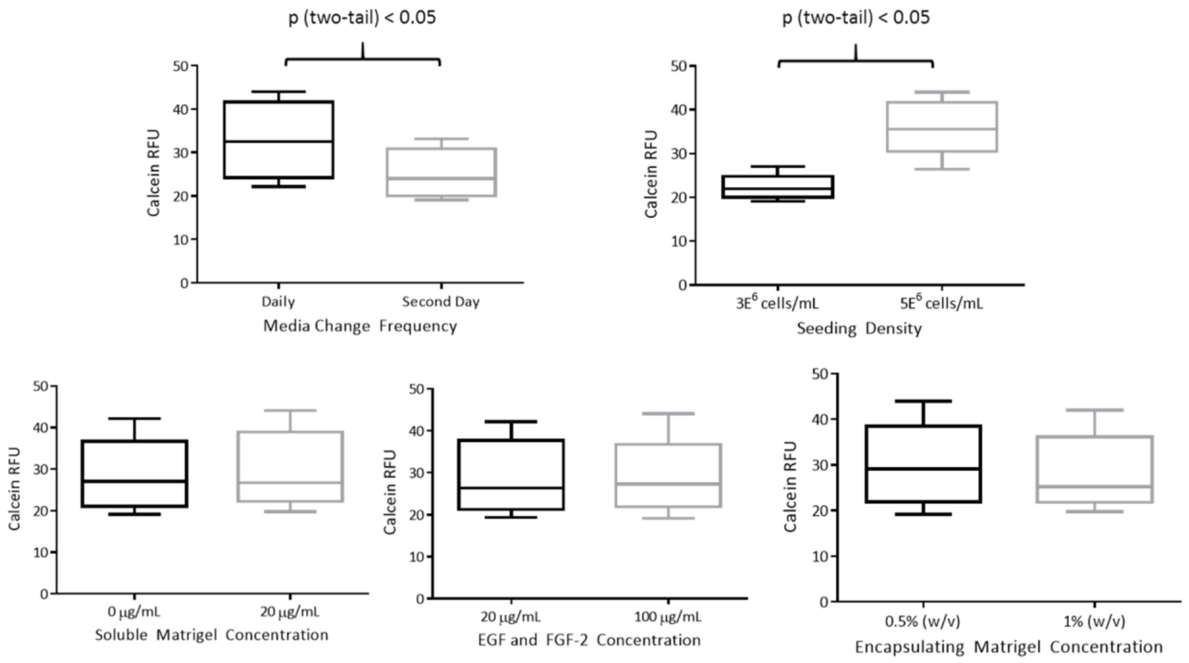

Figure S2 (related to Figure 2). Factorial design screen to identify NPC culture conditions on-chip. Five culture conditions were screened at two levels in a 2^5 factorial design experiment to identify factors influencing NPC growth on-chip. Average calcein fluorescence for each spot pertaining to each condition was entered into GraphPad Prism (24 independent biological replicates per condition) to generate Tukey box plots and perform student t-tests (two-tail). As expected, higher seeding density resulted in a higher calcein fluorescence intensity ($p < 0.05$). Media change frequency had a substantial and significant ($p < 0.05$) impact on growth and viability NPCs. Presence of Matrigel in media, growth factor concentration and encapsulating Matrigel concentration were not significantly influential on viability and growth.

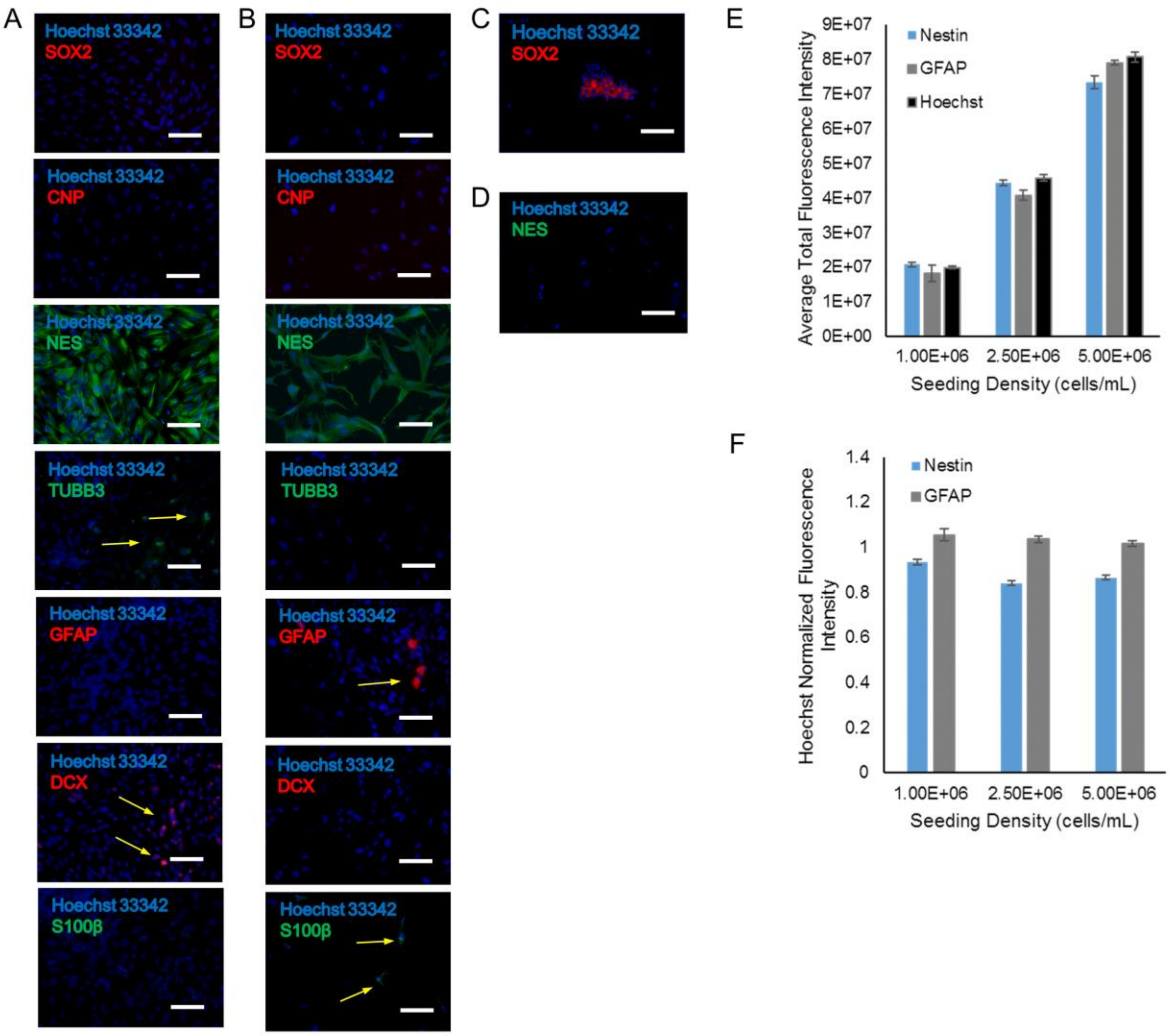

Figure S3 (related to Figure 3 and Figure 4). Immunofluorescence primary antibody validation and on-chip immunofluorescence assay sensitivity. Merged immunofluorescence images of (a) differentiated SK-N-SH neuroblastoma cells (b) CCF-STTG1 astrocytoma cells (c) human embryonic stem cells and (d) human mesenchymal stem cells incubated with antibodies specific for the indicated protein and Hoechst 33342 to counterstain nuclei. The merged images were processed in ImageJ by first adjusting brightness and contrast to eliminate background fluorescence using samples incubated only with the appropriate fluorescent secondary antibody. These single-channel images were then merged with the blue channel to merge with the stained nuclei, with the exception being the image depicted in (c). The blue channel was to generate a mask of nuclei that could be overlaid with the labelled protein channel since SOX2 and nuclei should co-localize and this makes for simplified confirmation of the proper subcellular location. As expected, the neuroblastoma cells in (a) were negative for SOX2 and glial protein markers. Also, the majority of cells were positive for NES. A small population of differentiated cells were positive for TUBB and DCX (indicated with yellow arrows), which together demonstrated the specificity of TUBB3, DCX and NES antibodies on neuronal populations. Notably, the differentiation of the neuroblastoma line yielded a heterogeneous mixture of differentiated and undifferentiated neurons, making it a good choice for validation of the neuronal specific antibodies. The astrocytoma cells in (b) were negative for SOX2 and neuronal protein markers. The majority of cells were also positive for NES. A small population of differentiated cells were positive for GFAP and S100β (indicated with yellow arrows), which together demonstrated the specificity of GFAP, S100β, and NES antibodies. The chosen cell line is a late stage glioma, which does not express high levels of astrocytic protein markers (i.e. GFAP, S100β), making it a good choice for screening specificity. Additionally, the human embryonic stem cells (c) were positive for Sox-2 and the human mesenchymal stem cells (d) were negative for NES, further demonstrating the specificity of these

antibodies. Scale bars = 100 µm. (e) To assess on-chip immunofluorescence assay sensitivity, a microarray chip was prepared with varied cell densities (1, 2.5 and 5 x 10^6 cells/mL) for detection of NES or GFAP expression with an on-chip immunofluorescence. The total fluorescence intensity was measured for an entire cell spot stained for either GFAP or NES and averaged (n = 54 biological replicates for each) to plot the mean ± SEM against seeding density. (f) Dividing the immunofluorescence intensity by the Hoechst 33342 intensity (nuclear stain) within each cell spot resulted in immunofluorescence signals normalized by the number of cells present within each spot. These normalized values were averaged (n = 54 biological replicates for each) for GFAP and NES to plot normalized intensity ± SEM against seeding density.

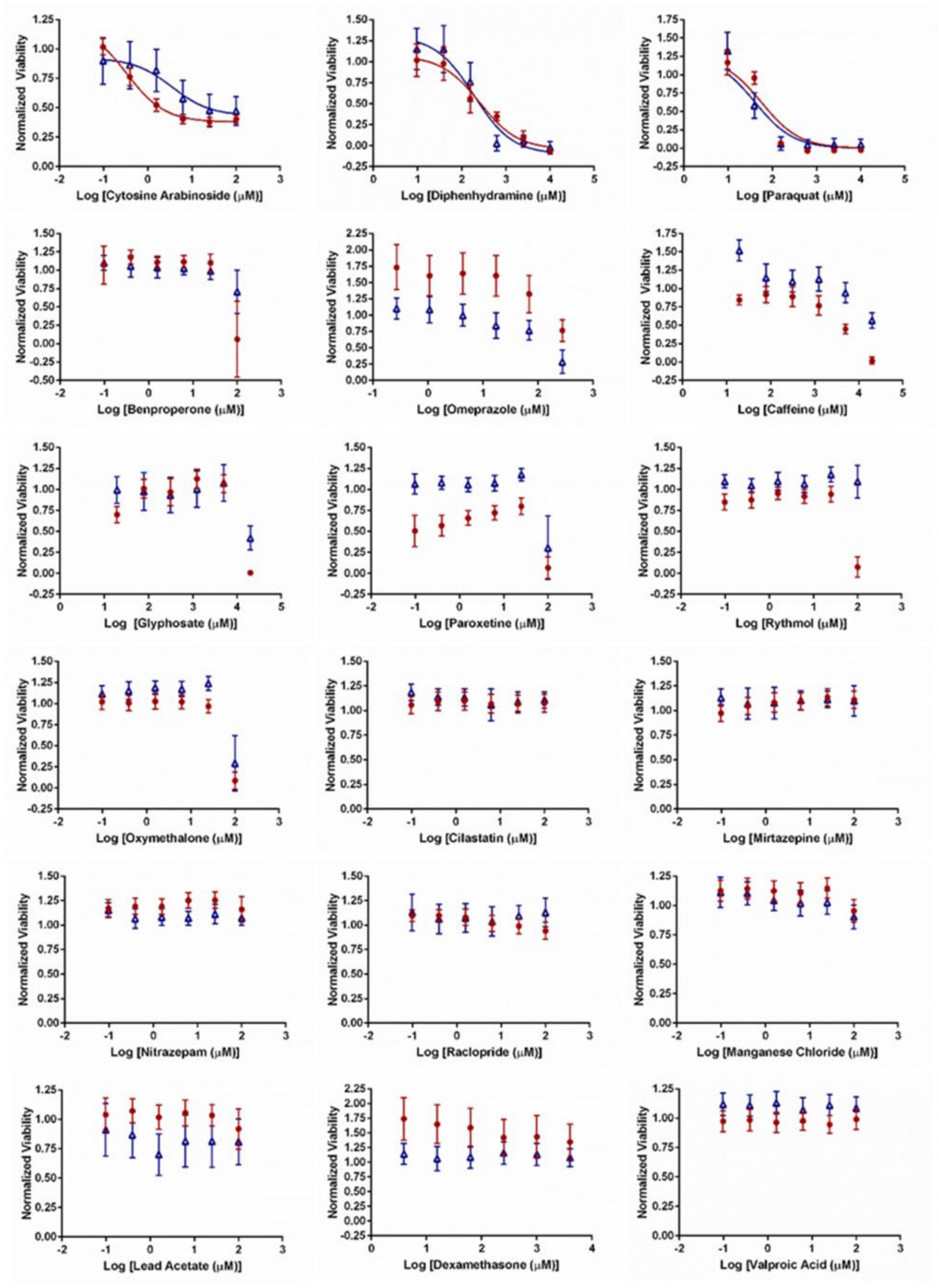

Figure S4 (related to Figure 5 and Table 1). Dose-response viability curves of undifferentiated and differentiating NPCs. Vehicle control normalized dose-response viability of undifferentiated (●) and differentiating (**Δ**) NPCs plotted against log(concentration (μM)). Mean viability ± SEM is plotted for each concentration as determined from 30 biological replicates per dose. Exceptions for this include undifferentiated raclopride (10 biological replicates), differentiated caffeine (20 biological replicates) and undifferentiated cytosine arabinoside (20 biological replicates). Sigmoidal fits to the data are plotted as solid lines when appropriate.

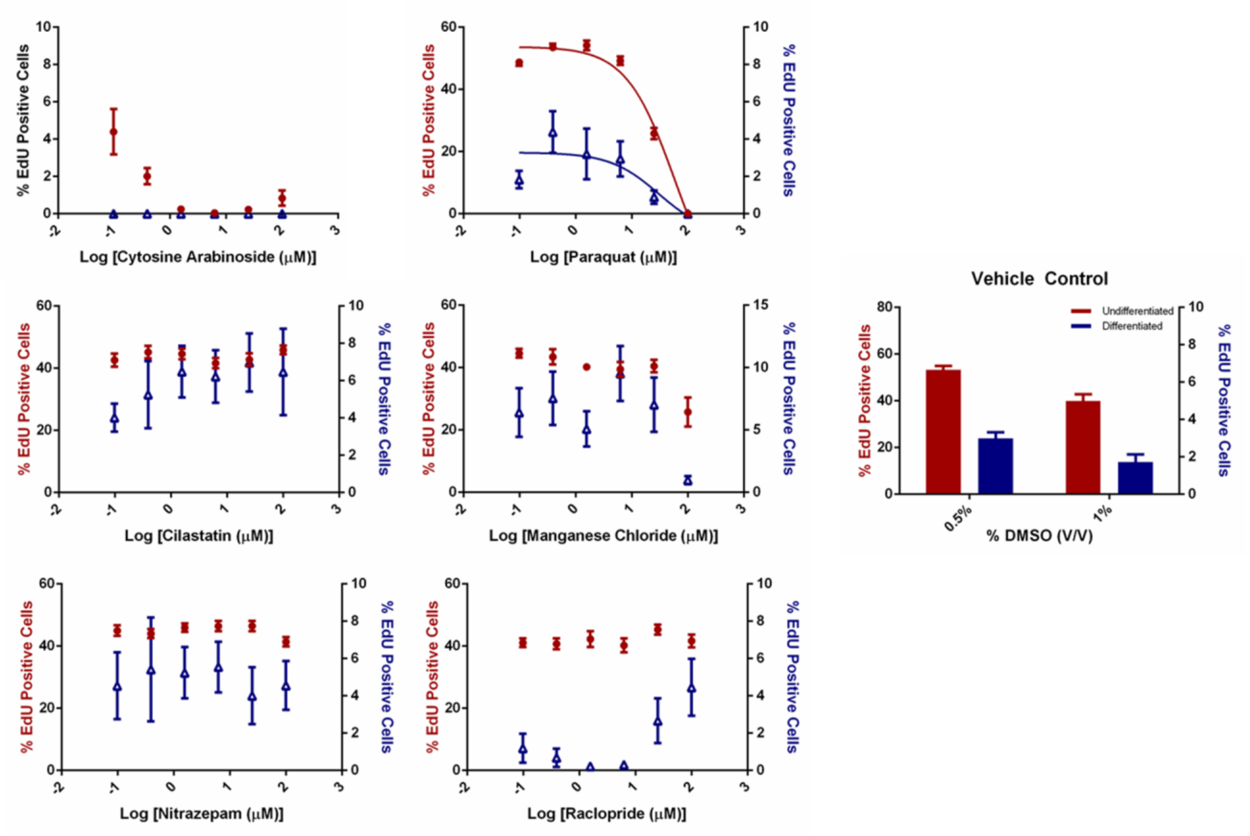

Figure S5 (related to Figure 5, Table 1). Dose-response proliferation curves of undifferentiated and differentiating NPCs. Dose-response EdU incorporation of undifferentiated (●, left axis) and differentiating (**Δ**, right axis except doxorubicin) NPCs plotted against log(concentration (μM)). Mean % EdU positive nuclei ± SEM is plotted for each concentration as determined from 10 biological replicates.

Antibody	Vendor	Application	Dilution
Chicken monoclonal anti-CNPase	Abcam	IF	1:100
Rabbit polyclonal anti-doublecortin	Abcam	IF, WB	1:250 (IF), 1:1000 (WB)
Rat monoclonal anti-GFAP	Invitrogen	IF	1:250
Mouse monoclonal anti-Nestin	Abcam	IF, WB	1:250 (IF), 1:1000 (WB)
Mouse monoclonal anti-βIII tubulin	Abcam	IF, WB	1:250 (IF), 1:1000 (WB)
Rabbit monoclonal anti-Sox2	Abcam	IF, WB	1:100 (IF), 1:1000 (WB)
Mouse monoclonal anti- S100β	Abcam	IF, WB	1:100 (IF), 1:500 (WB)
Mouse monoclonal anti-GFAP	Abcam	WB	1:250
Rabbit monoclonal anti-CNPase	Cell Signaling Tech	WB	1:1000
Mouse monoclonal anti-Vinculin	Sigma	WB	1:200
Mouse monoclonal anti-GAPDH	Abcam	WB	1:500
Rabbit monoclonal anti-Sox2	Cell Signaling Tech	WB	1:1000
Goat anti-mouse Alexa 488	Invitrogen	IF	1:100
Goat anti-rat Alexa 549	Invitrogen	IF	1:100
Goat anti-rabbit Alexa 549	Invitrogen	IF	1:100
Goat anti-chicken Alexa 549	Invitrogen	IF	1:100
Goat anti-mouse Horseradish Peroxidase	Invitrogen	WB	1:5000
Goat anti-rabbit Horseradish Peroxidase	Invitrogen	WB	1:5000

Table S1 (related to experimental methods). Compiled table of antibodies used in this work**.** WB = Western Blot; IF = immunofluorescence

Chemical	Chemical classification	Concentration range screened (μM)	Vendor	Vehicle (% DMSO)
Acetaminophen	analgesic	80 – 80,000	Sigma	0.5
Raclopride	anti-psychotic	0.1 – 100	NIH Clinical Collection	1
Nitrazepam	anti-anxyletic	0.1 – 100	NIH Clinical Collection	1
5-Fluorouracil	chemotherapeutic	0.1 – 100	Sigma	0.5
Cytosine Arabinoside	chemotherapeutic	0.1 – 100	Sigma	0.5
Retinoic Acid	chemotherapeutic	0.1 – 100	Sigma	0.5
Doxorubicin	chemotherapeutic	0.1 – 100	Sigma	0.5
Cadmium Chloride	heavy metal	0.1 – 100	Sigma	0.5
Manganese Chloride	heavy metal	0.1 – 100	Sigma	0.5
Cilastatin	statin	0.1 – 100	NIH Clinical Collection	1
Pitavistatin	statin	0.1 – 100	NIH Clinical Collection	1
Diphenhydramine	anti-histamine	10 – 10,000	Sigma	0.5
Dexamethasone	steroid	4 – 4000	Sigma	0.5
Oxymethalone	steroid	0.1 – 100	NIH Clinical Collection	1
Caffeine	stimulant	20 – 20,000	Sigma	0.5
Rythmol	anti-arrhythmic	0.1 – 100	NIH Clinical Collection	1
Paroxetine	anti-depressant	0.1 – 100	NIH Clinical Collection	1
Mirtazapine	anti-depressant	0.1 – 100	NIH Clinical Collection	1
Valproic Acid	anti-epileptic	0.1 – 100	NIH Clinical Collection	1
Benproperine	anti-tussive	0.1 – 100	NIH Clinical Collection	1
Lead Acetate	heavy metal	0.1 – 100	Sigma	0.5
Glyphosate	herbicide	20 – 20,000	Sigma	0.5
Omeprazole	proton pump inhibitor	0.1 – 100	Sigma	0.5
Paraquat	herbicide	10 – 10,000 (viability)<br>0.1 – 100 (proliferation)	Sigma	0.5

Table S2 (related to experimental methods, Figure 5 and Table 1). List of chemicals screened for viability and proliferation effects with the concentration range screened, vendor, and vehicle control used.

Chemical	Normalized viability at C_{max}	
	Undifferentiated Mean ± SEM	Differentiating Mean ± SEM
Lead Acetate	0.92 ± 0.17	0.81 ± 0.19
Glyphosate	0.01 ± 0.03	0.41 ± 0.14
Cytosine Arabinoside	0.40 ± 0.03[a]	0.47 ± 0.12
Paraquat	-0.02 ± 0.03	0.05 ± 0.02
Diphenhydramine	-0.07 ± 0.02	-0.02 ± 0.08
Caffeine	0.02 ± 0.04	0.56 ± 0.10[a]
Retinoic Acid	0.55 ± 0.16	0.50 ± 0.13
Omeprazole	0.76 ± 0.17	0.29 ± 0.18
Dexamethasone	1.35 ± 0.33	1.08 ± 0.16
Cadmium Chloride	0.06 ± 0.07	0.09 ± 0.04
5-Fluorouracil	0.15 ± 0.10	0.81 ± 0.17
Acetaminophen	-0.16 ± 0.07	-0.1 ± 0.03
Manganese Chloride	0.96 ± 0.10	0.90 ± 0.10
Cilastatin	1.07 ± 0.10	1.11 ± 0.08
Oxymethalone	0.09 ± 0.10	0.29 ± 0.33
Valproic Acid	0.99 ± 0.08	1.09 ± 0.10
Pitavastatin	0.14 ± 0.11	1.16 ± 0.07
Raclopride	0.94 ± 0.08[b]	1.13 ± 0.15
Benproperone	0.06 ± 0.52	0.71 ± 0.30
Nitrazepam	1.16 ± 0.13	1.07 ± 0.07
Mitrazepine	1.11 ± 0.09	1.10 ± 0.15
Rythmol	0.08 ± 0.12	1.09 ± 0.20
Paroxetine	0.07 ± 0.13	0.30 ± 0.38
Doxorubicin	0.05 ± 0.13	0.11 ± 0.16

Table S3 (related to Figure 5 and Table 1). Vehicle-control normalized viability of undifferentiated and differentiating human NPCs at the maximum concentration tested (see Table S2). The data represent the normalized mean and SEM of 30 biological replicates. Chemicals in bold were deemed to be toxic because they exhibited ≥50% reduction in viability of either undifferentiated or differentiating cultures. [a]denotes a condition where the value is from 20 biological replicates. [b]denotes a condition where the value is from 10 biological replicates.

Supplemental Experimental Procedures

Cell culture of cell lines used in validation of primary antibodies

The neuroblastoma line SK-N-SH (ATCC) was cultured per manufacturer's recommendations. Specifically, the cells were cultured using Eagle's Minimum Essential Media (EMEM) supplemented with 10% fetal bovine serum (Sigma Aldrich). Cells were cultured in T75 flasks at 5% CO_2 and 37 ^oC. Media was exchanged twice weekly. Cells were passaged by rinsing with DPBS, incubating with trypsin-EDTA (Gibco) and suspended in EMEM media before centrifugation at 300 x g and resuspension in fresh medium. Differentiation of SK-N-SH was carried out as described by Jain et al. (Jain et al., 2007). Briefly, the cells were plated and cultured in a flask with media containing all-*trans* retinoic acid (RA, 10 µM) for one week. Following, the cells were re-passaged onto PLL-coated 96-well plates and cultured with EMEM containing 5% fetal bovine serum, 10 µM RA, and 1 µM cytosine arabinoside (to reduce proliferation of undifferentiated cells) for one week, at which time the cells were fixed for immunostaining. Cells were at passage number 5 or less upon use.

The astrocytoma line CCF-STTG1 (ATCC) was cultured per manufacturer's recommendations. Briefly, the cells were cultured with RPMI media supplemented with 10% fetal bovine serum. Cells were cultured in T75 flasks at 5% CO_2 and 37 ^oC. Media was exchanged every second day. Cells were passaged by rinsing with DPBS, incubating with trypsin-EDTA (Gibco) and suspended in EMEM media before centrifugation at 300 x g and resuspension in fresh medium. For primary antibody validation, cells were seeded to a 96-well plate and cultured overnight prior to fixation. Cells were at passage number 5 or less upon use.

The human embryonic stem cell line (hESC; RUES2) was provided as a 96-well plate of cells at passage 32, kindly donated by Dr. Brigitte Arduini of the Rensselaer Center for Stem Cell Research (RCSCR). Before fixation, the cells were maintained in mouse embryonic fibroblast-conditioned medium (MEF-CM) produced by the RCSCR supplemented with FGF2 (20 ng/mL; Invitrogen), cultured on Matrigel-coated 35 mm and 65 mm plates at 5% CO_2 and 37 ^oC. The media was changed daily and cells routinely passaged in clumps using dispase (Stem Cell Technologies). Upon receipt of the 96-well plate, the cells were immediately fixed and processed for immunostaining.

The human embryonic mesenchymal stem cells (hMSC; Lonza) were cultured according to the manufacturer's instructions with slight modifications. Maintenance medium consisted of DMEM supplemented with 10% fetal bovine serum (MSC qualified; Gibco) and medium was changed every second day. The cells were passaged with TrypLE (Gibco) after rinsing with DPBS upon reaching high confluence. The cells were cultured on T25 flasks and stored at 5% CO_2 and 37 ^oC. Cells were at passage number 10 or less when used. All immunofluorescence protocols used were identical to those used on ReNcell VM NPCs.

Confocal imaging of NPC in Matrigel

On-chip ReNcell VM cultures in either 0.5% or 1% Matrigel (~300 cells/100 nL spot) were seeded as described above and cultured for three days with daily media change. On the third day, cultures were stained with the viability assay, but the chips were kept in DPBS containing 1 g/L glucose instead of being dried. The stained chips were imaged using a Zeiss LSM multiphoton confocal fluorescence microscope. The first imaged plane was the top of the gel, which was found by adjusting focus until no more cells were observed. Images were taken every 10 µm toward the bottom of the cell spot. The bottom of the gel was determined when no more cells were observed. Z-stacks were constructed using ImageJ.

Factorial analysis of growth conditions

A factorial design experiment was performed to assess simultaneously multiple culture conditions. Two values of five different variables were screened for a total of 32 conditions tested in parallel with n=24 replicates. The conditions tested were media change frequency (daily or every second day), growth factor concentration (20 ng/mL or 40 ng/mL), Matrigel in the media (none or 20 µg/mL), seeding density (300 cells/spot or 500 cells/spot) and the concentration of encapsulating Matrigel (0.5% (w/v) or 1% (w/v)). On-chip cultures were prepared to accordingly to the screened conditions, and after five days of culture on-chip the chips were used with the viability assay. The average calcein fluorescence of each spot was measured and grouped for each condition tested for entry into GraphPad Prism 6.0 for generation of Tukey box plots to assess the overall impact of each condition. Two-tailed student's t-tests were performed to assess statistical significance for each factor tested.

Determination of after-printing viability

The day one PillarChip from the growth experiment was used in determining viability of NPCs in 1% (w/v) Matrigel one day after printing. Cellomics software was used to detect and select all nuclei within a cell spot by gating for regions of strong fluorescence. The detected nuclei were used as a mask in the green channel to determine whether green fluorescence was detected within the same region. If green staining overlaid with blue staining (thus, calcein staining and Hoechst 33342 staining was co-localized), the object was counted as a living cell. The percent viability was determined by dividing the number of living cells (co-localized green and blue stains) by the total number of detected nuclei and multiplying by 100. The average and standard deviation were determined from n=396 replicates from a single printed chip for each 0.5% and 1% Matrigel conditions.

In-cell immunofluorescence imaging for monolayer cultures and antibody validation

Samples processed for immunofluorescence analysis of 2D monolayer cultures and primary antibody validation were prepared as described previously. The samples were imaged using an IX51 Olympus microscope with filters for DAPI, FITC and Texas Red. Images taken with the DAPI filter were exposed for 5 ms. Images taken with the FITC and Texas Red filters were exposed for 500 ms. Exceptions to this were differentiated ReNcell VM cells images for GFAP. The GFAP intensity was so high on the differentiated cultures that samples had to be imaged with a 15 ms exposure time. Images were transported to ImageJ for processing and compilation.

Cellomics high-content analysis

For viability assay samples, images were acquired at both 50X and 100X magnification with a multiple bandpass emission and excitation filters (BGRFR) in blue (386), green (485) and red (549) channels using 2.5 millisecond exposures for the blue and green channels and a 15 ms exposure for the red channel. Images from 50X magnification were used to measure the total fluorescence in the green channel, which was normalized by the total number of pixels within the image (constant for all measurements) to determine calcein staining intensity. The total fluorescence in the blue channel measured for Hoechst 33342 fluorescence. Red fluorescence from ethidium homodimer-I was not used in analysis, and was therefore not quantified. Images from 100X magnification were used only for determination of after-printing viability.

For on-chip, in-cell immunofluorescence assay samples, images were acquired at 100X magnification with a multiple bandpass emission filter and excitation filters (BGRFR). Images were collected for blue (386), green (485), red (549) and far-red (650) channels using exposure times of 2, 30, 75, and 50 ms, respectively. The red (549) channel was also captured with a 7.5 ms exposure for GFAP analysis, which had significantly higher staining intensities. Within each cell spot, the total fluorescence in each channel measured using the Cellomics analytical software. This measured intensity was normalized by the total intensity from the blue channel to account for cell number within each spot. The normalized non-specific signal from the appropriate fluorescent secondary antibody was subtracted from the normalized intensities associated with each protein marker to account for background staining. These values were then plotted for assessing the expression levels of protein markers. The background subtracted, normalized fluorescence intensities were averaged over n = 27 spots for each protein assessed, omitting detached cell spots from analysis. Student's t-tests (two-tailed) were done for statistical analysis in comparing these corrected mean intensities between undifferentiated and differentiated samples.

Growth analysis and viability assay sensitivity of on-chip 3D hNPC cultures

Four NPC PillarChip cultures were prepared as described previously in 0.5% and 1% (w/v) Matrigel and cultured in WellChips containing complete growth media with daily media change. After one, three, five, and seven days of culture on-chip, an entire PillarChip was assayed using the live/dead viability assay. The background calcein fluorescence from dead cell control spots (n=36) was averaged and subtracted from the average calcein fluorescence intensity from living cell spots (396 replicates). Detached cell spots were omitted from analysis (< 5% of the total cell spots). The adjusted average calcein fluorescence intensity for each time point (Eq. 1) was used in generating growth curves.

$$F_{calcein} = \bar{F}_{485,\ sample} - \bar{F}_{485,\ dead\ control} \qquad (1)$$

For the viability sensitivity assay, a PillarChip was prepared with 100 nL spots of Matrigel-cell suspension of either 0.5% or 1% Matrigel at densities of 0, 1 x 10^6, 2.5 x 10^6, 5 x 10^6, 7.5 x 10^6, and 10 x 10^6 cells/mL and stamped into a WellChip for culture overnight. The following day, chips were stained with the viability assay. The

pixel-normalized calcein fluorescence was adjusted for background staining as described in Eq. 1, however instead of the background fluorescence from dead cells the green fluorescence intensity from the 0 cells/mL condition was used to account for background staining. This background-adjusted condition was averaged (n=72) and plotted against seeding density. The total Hoechst 33342 fluorescence was averaged over each condition (n=72) and the total fluorescence from the 0 cells/mL condition was subtracted to account for background fluorescence and plotted against seeding density.

Western blot sample preparation

For 3D Western blot analysis, ReNcell VM cells were seeded at 890,000 cells/mL in 1% (w/v) Matrigel in 8-well chamber microscope slides (Nunc). Cell suspension (100 µL) was placed into each chamber and allowed to gel for 20 min at 37 ^{o}C. After gelation, 250 µL of medium was added to each well and media was changed daily. Cell lysate was collected for undifferentiated cultures after three days of culture in complete growth media. Differentiated cell lysate was collected after five days of growth in complete growth media followed by a 10-day differentiation in differentiation media. Prior to collection of lysates, 3D cultures were rinsed once with ice cold PBS before 2 h incubation with a Cell Recovery solution (BD) at 4 ^{o}C for Matrigel depolymerization. The cell suspension was centrifuged at 300 g for 5 min at 4 ^{o}C and cells were re-suspended in cold Cell Recovery solution. The suspension was centrifuged again and re-suspended in cold PBS prior to a third centrifugation. The pellet was lysed in 250 µL RIPA buffer containing protease inhibitor cocktail (Gold Biotechnology) and stored at -20 ^{o}C. For 2D Western blot analysis, ReNcell VM cells were seeded at 100,000 cells/well in complete growth media into laminin-coated 35 mm dishes. Media was changed daily, and once 100% confluence was reached, lysate was collected for the undifferentiated sample and media was changed to differentiation media for the differentiated sample. Media was changed daily for the differentiation sample for 10 days, at which time lysate was collected. The 2D cultures were rinsed once with ice cold PBS prior to lysate collection in 250 µL RIPA buffer containing protease inhibitor cocktail, after which samples were stored at -20 ^{o}C.

On-chip toxicity assay analysis

The average adjusted calcein fluorescence for condition was determined as described above (Eq. 1). The vehicle control adjusted calcein fluorescence was determined in a similar manner (Eq. 2), and the normalized viability was determined by dividing the calcein fluorescence of a sample by the calcein fluorescence of a vehicle control (Eq. 2 and Eq. 3). Detached cell spots and cell spots with intensities larger than two standard deviations from the mean of each condition were excluded from analysis.

$$F_{vehicle\ control\ \ calcein} = \bar{F}_{485,\ \ vehicle\ control} - \bar{\bar{F}}_{485,\ \ dead\ control} \qquad (2)$$

$$Normalized\ Viability = \frac{F_{sample\ calcein}}{F_{vehicle\ control\ calcein}} \qquad (3)$$

These averaged viabilities and their associated standard errors of the mean were used with GraphPad Prism 6.0 (GraphPad Software) to fit sigmoidal dose response curves with constant slopes using Eq 4. For dose-response curves without clearly defined top or bottom plateaus, these values were set to either the maximum or minimum viability values within the sampled range (e.g. "Top" constraint for paraquat fit set to 1.25 and "Bottom" constraint for retinoic acid fit set to "0"). The log(IC_{50}) values were obtained from the dose response curves generated in GraphPad Prism 6.0. Student's t-tests (two-tail) were performed for statistical analysis on the log(IC_{50}) values.

$$Y = Bottom + \frac{(Top - Bottom)}{(1 + 10^{\log EC50 - X})} \qquad (4)$$

Supplemental References

Jain, P., Cerone, M.A., LeBlanc, A.C., Autexier, C., 2007. Telomerase and neuronal marker status of differentiated NT2 and SK-N-SH human neuronal cells and primary human neurons. J. Neurosci. Res. 85, 83–89. doi:10.1002/jnr.21094
